# Global, Regional, and National Burden of Breast Cancer, 1990–2021, and Projections to 2050: A Systematic Analysis of the Global Burden of Disease Study 2021

**DOI:** 10.1111/1759-7714.70052

**Published:** 2025-05-04

**Authors:** Tong Deng, Hao Zi, Xing‐Pei Guo, Li‐Sha Luo, Ya‐Long Yang, Jin‐Xuan Hou, Rui Zhou, Qian‐Qian Yuan, Qing Liu, Qiao Huang, Gao‐Song Wu

**Affiliations:** ^1^ Department of Thyroid and Breast Surgery Zhongnan Hospital of Wuhan University Wuhan China; ^2^ Center for Evidence‐Based and Translational Medicine Zhongnan Hospital of Wuhan University Wuhan China; ^3^ Evidence‐Based Medicine Center Xiangyang No. 1 People's Hospital, Hubei University of Medicine Xiangyang China; ^4^ Department of General Surgery Zhengzhou Central Hospital Affiliated to Zhengzhou University Zhengzhou China; ^5^ Department of Breast Surgery Hubei Cancer Hospital, Tongji Medical College, Huazhong University of Science and Technology, Hubei Provincial Clinical Research Center for Breast Cancer, Wuhan Clinical Research Center for Breast Cancer Wuhan China; ^6^ Department of Physical Examination Zhengzhou Central Hospital Affiliated to Zhengzhou University Zhengzhou China

**Keywords:** breast cancer, cancer epidemiology, global burden of disease, prediction, risk factors

## Abstract

**Background:**

This study analyzes the global burden of breast cancer (BC) over the past 30 years, identifies key risk factors, and projects future incidence and mortality through 2050.

**Methods:**

Data were sourced from the 2021 Global Burden of Disease (GBD) database. The estimated annual percentage change (EAPC) was used to assess trends, and country development was measured using the Socio‐Demographic Index (SDI). Projections were conducted using Bayesian age‐period‐cohort and autoregressive integrated moving average models.

**Results:**

In 2021, approximately 2.12 million new breast cancer cases and 674 199 deaths were recorded globally. From 1990 to 2021, incidence and prevalence increased, while mortality and disability‐adjusted life years (DALYs) declined overall. Regional and national variations were observed, alongside age and gender differences in the disease burden. A diet high in red meat and a high body mass index were the leading global risk factors for breast cancer deaths. The BC burden was positively correlated with SDI across 21 GBD regions. Decomposition analysis highlighted demographic factors as the main drivers of increased disease burden over the past three decades. Projections indicate that BC incidence will continue to rise through 2050.

**Conclusions:**

While global BC mortality has decreased over the past 30 years, incidence continues to rise. Low‐SDI regions face increasing challenges, as incidence, mortality, and DALYs persistently climb. These findings underscore the need for targeted public health strategies and equitable resource distribution to mitigate the rising burden of breast cancer.

## Graphical abstract

Breast cancer is one of the most prevalent cancers worldwide and represents a significant global health burden. This study utilizes the latest GBD 2021 data and advanced methodologies to examine the global burden of breast cancer from 1990 to 2021, with projections extending to 2050. The summary chart presents the global incidence statistics of breast cancer for the years 1990 and 2021.

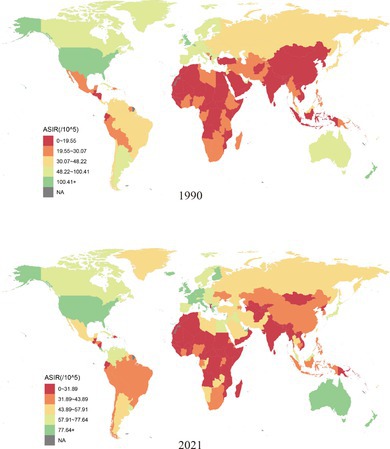

## 1 Introduction

Cancer remains a leading cause of death worldwide and poses a significant barrier to increasing life expectancy [1]. Breast cancer (BC) is the most commonly diagnosed cancer among women globally and a major cause of cancer‐related mortality. According to the 2022 Global Cancer Statistics, BC accounted for 2.3 million new cases, representing 11.6% of all cancers, and caused 666 000 deaths, making it the fourth leading cause of cancer deaths worldwide [2]. Although male BC comprises approximately 1% of all BC cases globally, its incidence appears to be rising [3]. With the aging and growth of the global population, increasing life expectancy, socioeconomic development, and the emergence of the COVID‐19 pandemic, the incidence and mortality rates of BC have undergone significant changes [4, 5, 6]. Consequently, periodic assessments of the BC burden and analyses of epidemiological trends are crucial for shaping future health policies.

The Global Burden of Disease (GBD) study is an ongoing effort aimed at quantifying health outcomes worldwide [4]. GBD 2021 employed additional data sources and refined methodologies to generate updated annual estimates for global, regional, national, and subnational levels, covering mortality, life expectancy, and population sizes from 1950 to 2021 [4]. Previous studies have assessed the burden of BC‐related risks [7, 8, 9, 10, 11]. This study leverages the latest GBD 2021 data and refined methods to examine the global breast cancer burden from 1990 to 2021 and predict trends up to 2050. It also provides an in‐depth analysis of regional variations, particularly the impact of the Social Development Index (SDI), and highlights challenges in low‐ and middle‐income regions. Additionally, this study explores the influence of sociodemographic factors on the disease burden, offering a more nuanced understanding compared to previous research.

## 2 Materials and Methods

### 2.1 Data Sources and Collection

Information on breast cancer was gathered using the Global Health Data Exchange online tool (http://ghdx.healthdata.org/gbd‐results‐tool). The methods applied in this study have been previously described in detail [12, 13]. The SDI quantifies regional socio‐demographic progress based on income, education, and fertility rates. GBD 2021 categorizes causes into four levels; breast cancer, a Level 3 cause, falls under Level 2 tumors within Level 1 non‐communicable diseases. Breast cancer is defined by the International Classification of Diseases, 10th Revision (ICD‐10), codes C50‐C50.629, C50.8‐C50.929.7.

### 2.2 Attributable Risk Factors

The GBD classifies Level 1 risk factors into behavioral, metabolic, and environmental/occupational risks. Attributable risk factors for breast cancer include seven risks: alcohol consumption, diets high in red meat, smoking, secondhand smoke, and low physical activity as behavioral risks, and high body mass index (BMI) and high fasting plasma glucose (FPG) as metabolic risks [4]. This study analyzed the burden of breast cancer and trends in these behavioral and metabolic risks.

### 2.3 Prediction Model

The Bayesian Age‐Period‐Cohort (BAPC) model was utilized to predict the future burden of breast cancer, focusing on correlations between morbidity or mortality and age structure and population size. This model employs a second‐order random walk to smooth priors for age, period, and cohort effects, yielding more accurate predictions than other methods [14]. The autoregressive integrated moving average (ARIMA) model consists of the autoregressive (AR) model and the moving average (MA) model. The underlying assumption is that data series are time‐dependent random variables, whose autocorrelation can be characterized by the ARIMA model, and future values can be predicted based on past values. The equation is expressed as *Yt* = *ϕ*1*Yt* − 1 + *ϕ*2*Yt* − 2 + … + *ϕpYt* −*p* + *et* − *θ*1*et* − 1 − … − θ*qet*−*q*, where (*ϕ*1*Yt* − 1 + *ϕ*2*Yt* − 2 + … + *ϕpYt* −*p* + *et*) is the AR model part, *et* − *θ*1*et* − 1 − … − θ*qet*−*q* is the MA model part, *Yt* − *p* is the observed value at the period of (*t* − *p*), *p* and *q* represent the model order of AR and MA, and et is the random error at the period of *t* [15]. The time series in the ARIMA model should be a stationary and stochastic sequence with zero mean. The time series in the ARIMA model should be a stationary random series with zero mean. We used a BAPC model incorporating a nested Laplace approximation to forecast the disease burden from 2022 to 2050. We applied an ARIMA model for sensitivity analysis to verify the robustness of the forecasts.

### 2.4 Decomposition Analysis

Decomposition analysis was conducted to determine specific factors influencing changes in incidence, deaths, prevalence, and disability‐adjusted life years (DALYs) over time at global, regional, and national levels. This approach evaluated the effects of aging, demographic changes, and epidemiological transitions.

### 2.5 Statistical Analysis

This study employed age‐standardized rates (ASR) and their 95% uncertainty intervals (UI) to quantify the incidence, mortality, prevalence, and DALYs of BC over time, by sex, region, country, and SDI. ASR refers to the weighted average of age‐specific rates, where the weights are derived from a standard age distribution. This method adjusts for differences in age distributions between populations, allowing for fair comparisons across regions and time. DALYs represent the total burden of a disease, combining years of life lost (YLL) due to premature mortality and years lived with disability (YLD). This comprehensive metric allows for the comparison of the impact of diseases across populations. Then, using changes in cancer cases from 1990 to 2021, the EAPC and its 95% confidence intervals (CI) were used to assess the trends in BC incidence and mortality. The 95% UI were reported for all estimates. The ASR (per 100 000 population) was calculated as the sum of the products of age‐specific rates(*ai*, where *i* denotes the *ith* age) and the population number (or weight *wi*) in the same age group of the selected reference standard population, divided by the total of the standard population weights: $\mathrm{ASR}=\mathrm{ASR}=\frac{\sum_{i=1}^{A}a_{i}w_{i}}{\sum_{i=1}^{A}w_{i}}\times 100\;000$. The EAPC was used to describe the trend of ASR within a specified time interval. The EAPC was estimated by a linear regression model: *y* = *α* + *βx* + *ε*, where *y* is ln (ASR), *x* is the calendar year, and *ε* is the error term. The EAPC was calculated as 100 × (exp(*β*)−1), and its 95% CI can be obtained from the linear regression model [16, 17]. When the estimated EAPC value and its lower 95% CI were both > 0, the ASR is considered as having an upward trend. Conversely, if the estimated EAPC value and its upper 95% CI were both < 0, the ASR is considered a downward trend. R software (version 4.2.2 and version 4.3.3), Microsoft Excel (Version 2019), GraphPad Prism (version 8.0.2) and Adobe Illustrator 2021(version 25.0) were used for statistics and visualization. A two‐sided *p* value < 0.05 was considered as statistical significance.

## 3 Results

### 3.1 Global Level

In 2021, the global incidence of BC was approximately 2.12 million cases, with an age‐standardized incidence rate (ASIR) of 24.56/100000 (95% UI: 22.93–26.26). Between 1990 and 2021, the global ASIR showed an upward trend, with an EAPC of 0.38 (95% CI: 0.33–0.42) (Table 1). BC mortality reached around 670 000 cases, with an age‐standardized mortality rate (ASMR) of 7.90/100000 (95% UI: 7.27–8.44), exhibiting a downward trend with an EAPC of −0.59 (95% CI: −0.64 to −0.54) (Table 1). The prevalence was 2.06 million cases, with an age‐standardized prevalence rate (ASPR) of 238.86/100 000 (95% UI: 226.17–252.24), increasing annually by 0.33% from 1990 to 2021. The DALYs were 2.06 million, with an age‐standardized disability‐adjusted life year rate (ASDR) of 239.03/100000 and an EAPC of −0.46 (Table S1).

**TABLE 1:** Incidence and mortality cases for breast cancer in 2021 for both sexes and its EAPC.

Characteristics	Counts (2021)	Age‐standardized incidence rate (per 100 000) (95% UI)		EAPC (95% CI) 1990–2021	Counts (2021)	Age‐standardized mortality rate (per 100 000) (95% UI)		EAPC (95% CI) 1990–2021
		1990	2021			1990	2021	
Global	2 121 564 (1982143–2 268 723)	21.38 (20.27–22.22)	24.56 (22.93–26.26)	0.38 (0.33–0.42)	674 199 (623372–720 823)	9.16 (8.57–9.61)	7.90 (7.27–8.44)	−0.59 (−0.64–0.54)
Female	2 082 737 (1940351–2 225 083)	39.99 (38.01–41.60)	46.40 (43.26–49.56)	0.40 (0.35–0.45)	660 925 (609171–707 182)	16.60 (15.60–17.45)	14.55 (13.45–15.56)	−0.55 (−0.60–0.50)
Male	38 827 (24650–47 846)	0.52 (0.46–0.60)	0.94 (0.60–1.15)	2.21 (2.05–2.37)	13 274 (9074–16 240)	0.28 (0.24–0.34)	0.34 (0.23–0.41)	0.66 (0.57–0.76)
Low SDI	78 125 (68896–87 591)	8.23 (7.08–9.47)	12.84 (11.35–14.30)	1.39 (1.25–1.54)	47 441 (41887–53 335)	6.53 (5.64–7.50)	8.66 (7.66–9.66)	0.88 (0.76–1.00)
Low‐middle SDI	239 540 (216755–261 136)	7.39 (6.64–8.31)	14.78 (13.36–16.10)	2.27 (2.23–2.31)	118 456 (106615–129 671)	5.11 (4.55–5.76)	7.75 (6.96–8.50)	1.37 (1.32–1.41)
Middle SDI	552 881 (499159–612 199)	10.52 (9.70–11.49)	19.54 (17.67–21.62)	1.94 (1.90–1.99)	185 771 (168486–206 081)	5.99 (5.53–6.53)	6.79 (6.16–7.54)	0.28 (0.23–0.33)
High‐middle SDI	517 073 (468473–576 713)	21.19 (20.14–22.16)	27.24 (24.69–30.42)	0.76 (0.69–0.83)	148 442 (134918–161 651)	9.70 (9.14–10.17)	7.68 (6.98–8.37)	−0.92 (−1.02–0.82)
High SDI	731 762 (668800–764 116)	43.25 (41.36–44.25)	40.17 (37.29–41.71)	−0.27 (−0.39–0.16)	173 264 (153063–183 925)	13.44 (12.69–13.87)	8.38 (7.55–8.82)	−1.62 (−1.66–1.57)
Central Europe, eastern Europe, and central Asia	182 024 (169176–195 443)	23.82 (23.1924.40)	29.50 (27.41–31.70)	0.51 (0.41–0.62)	67 985 (62858–73 426)	11.36 (11.03–11.64)	10.66 (9.86–11.52)	−0.48 (−0.64–0.31)
Central Asia	15 435 (13776–17 276)	19.75 (18.59–20.78)	16.91 (15.16–18.85)	−0.22 (−0.30–0.14)	6646 (5947–7417)	10.74 (10.12–11.30)	7.78 (6.99–8.63)	−0.75 (−0.84–0.66)
Central Europe	65 494 (60228–70 802)	24.87 (23.83–25.87)	33.17 (30.56–35.96)	0.85 (0.70–1.01)	25 216 (22972–27 335)	12.24 (11.71–12.79)	11.61 (10.64–12.58)	−0.35 (−0.45–0.25)
Eastern Europe	101 094 (90962–112 244)	23.99 (23.22–24.69)	30.60 (27.53–34.02)	0.49 (0.37–0.61)	36 123 (32263–40 814)	11.00 (10.64–11.30)	10.54 (9.41–11.94)	−0.55 (−0.78–0.33)
High‐income	773 262 (704621–809 643)	45.25 (43.28–46.30)	41.71 (38.74–43.33)	−0.29 (−0.42–0.17)	188 146 (164652–200 466)	14.15 (13.33–14.59)	8.72 (7.85–9.18)	−1.64 (−1.68–1.59)
Australasia	19 849 (17623–22 084)	43.53 (41.28–45.77)	42.36 (37.94–47.09)	−0.11 (−0.29–0.06)	4368 (3750–4929)	14.49 (13.56–15.34)	8.34 (7.31–9.29)	−1.85 (−1.91–1.79)
High‐income Asia Pacific	97 151 (85264–105 262)	15.18 (14.37–15.91)	28.44 (25.74–30.43)	2.13 (1.89–2.37)	20 227 (16846–22 166)	3.84 (3.66–3.97)	4.89 (4.32–5.23)	0.79 (0. 65–0.93)
High‐income North America	298 018 (274479–311 948)	66.89 (63.79–68.76)	50.57 (47.29–52.74)	−1.08 (−1.18–0.98)	59 590 (53306–63 192)	15.85 (14.89–16.39)	9.32 (8.46–9.84)	−1.86 (−1.91–1.80)
Southern Latin America	22 973 (21144–24 783)	26.28 (24.78–27.59)	27.45 (25.29–29.60)	0.17 (0.02–0.32)	10 084 (9080–10 945)	15.81 (14.84–16.63)	11.64 (10.53–12.59)	−0.93 (−1.06–0.80)
Western Europe	335 271 (302442–355 611)	45.27 (43.27–46.51)	43.09 (40.06–45.40)	−0.13 (−0.31–0.05)	93 877 (80683–101 173)	16.64 (15.66–17.20)	9.93 (8.82–10.57)	−1.73 (−1.78–1.69)
Latin America and Caribbean	165 769 (151918–181 474)	15.96 (15.43–16.40)	25.87 (23.74–28.31)	1.36 (1.27–1.45)	53 712 (49411–58 008)	7.87 (7.53–8.15)	8.55 (7.85–9.24)	0.13 (0.08–0.18)
Andean Latin America	10 899 (8537–13 856)	10.20 (8.76–11.79)	17.51 (13.73–22.33)	1.54 (1.40–1.69)	4118 (3257–5195)	6.32 (5.39–7.33)	6.82 (5.39–8.62)	0.01 (−0.12–0.13)
Caribbean	15 175 (12898–17 380)	22.55 (21.13–24.08)	28.46 (24.17–32.62)	0.82 (0.73–0.91)	5687 (4808–6683)	10.19 (9.42–11.08)	10.58 (8.94–12.43)	0.22 (0.15–0.28)
Central Latin America	77 200 (67263–87 273)	15.43 (14.94–15.86)	29.39 (25.65–33.20	1.87 (1.77–1.98)	19 491 (17035–21 942)	6.35 (6.12–6.52	7.63 (6.67–8.58)	0.47 (0.37–0.57)
Tropical Latin America	62 495 (58527–66 095)	15.90 (15.23–16.52)	23.75 (22.21–25.13)	1.02 (0.92–1.12)	24 417 (22598–25 922)	8.99 (8.51–9.37)	9.43 (8.71–10.03)	−0.02 (−0.09–0.04)
North Africa and Middle East	128 349 (114487–144 654)	8.19 (7.37–9.27)	23.74 (21.16–26.69)	3.97 (3.81–4.13)	30 136 (26534–34 261)	3.76 (3.39–4.23)	6.11 (5.41–6.92)	2.03 (1.89–2.18)
South Asia	209 425 (183326–238 853)	6.37 (5.65–7.19)	12.67 (11.10–14.50)	2.18 (2.08–2.28)	108 085 (94490–123 379)	4.60 (4.03–5.22)	6.93 (6.07–7.94)	1.26 (1.18–1.34)
Southeast Asia, east Asia, and Oceania	559 353 (472834–664 130)	9.51 (8.12–11.15)	19.27 (16.30–22.88)	2.32 (2.26–2.38)	163 633 (140833–189 737)	5.30 (4.55–6.16)	5.73 (4.95–6.63)	0.07 (−0.02–0.16)
East Asia	418 198 (327546–520 456)	9.12 (7.50–11.00)	19.43 (15.21–24.14)	2.50 (2.42–2.58)	96 401 (76552–118 997)	4.71 (3.91–5.63)	4.48 (3.56–5.52)	−0.43 (−0.54–0.32)
Oceania	1572 (1284–1968)	14.57 (11.61–18.06)	16.34 (13.59–19.97)	0.21 (0.08–0.34)	957 (784–1181)	9.69 (7.74–12.12)	10.90 (9.06–13.21)	2.03 (1.89–2.18)
Southeast Asia	139 583 (116510–168 516)	10.74 (9.11–12.74)	19.43 (15.21–24.14)	1.83 (1.76–1.90)	66 275 (55066–80 748)	7.19 (6.06–8.61)	9.55 (7.99–11.54)	0.87 (0.79–0.95)
Sub‐Saharan Africa	103 382 (87860–119 637)	11.20 (10.02–12.37)	18.21 (15.83–20.84)	1.66 (1.56–1.76)	62 502 (53883–71 614)	8.88 (7.93–9.79)	12.35 (10.90–13.97)	1.13 (1.07–1.20)
Central sub‐ Sharan Africa	10 097 (7540–13 198)	9.61 (7.01–12.66)	14.96 (11.31–19.51)	1.45 (1.26–1.65)	6361 (4733–8364)	7.76 (5.76–10.00)	10.61 (8.10–13.77)	1.04 (0.90–1.18)
Eastern sub‐ Sharan Africa	34 527 (29176–40 787)	11.13 (9.46–13.34)	16.83 (14.54–19.56)	1.25 (1.13–1.38)	21 340 (18274–25 025)	9.13 (7.80–10.96)	11.87 (10.34–13.80)	0.79 (0.69–0.88)
Southern sub‐ Sharan Africa	15 278 (13704–16 888)	14.21 (11.80–16.67)	24.25 (21.92–26.74)	2.23 (2.01–2.45)	8553 (7744–9446)	9.92 (8.14–11.76)	14.88 (13.56–16.35)	1.71 (1.47–1.95)
Western sub‐ Saharan Africa	43 480 (32854–57 198)	10.60 (8.74–12.47)	18.49 (14.52–23.65)	1.89 (1.73–2.05)	26 248 (20506–33 461)	8.44 (6.97–9.89)	12.40 (10.12–15.45)	1.32 (1.19–1.45)

Abbreviations: CI: confidence interval; EAPC: estimated annual percentage change; SDI: socio‐demographic index; UI: uncertainty interval.

### 3.2 Regional Level

In 2021, the study found that the ASIR of BC was highest in high‐SDI regions, at 40.17/100000 (95% UI: 37.29–41.71). In terms of geographical distribution, high‐income North America had the highest ASIR, at 50.57/100000 (95% UI: 47.29–52.74). South Asia had the lowest, at 12.67/100000 (95% UI: 11.10–14.50) (Table 1). Globally, the disease burden of breast cancer ASMR and ASDR is similar, and the heaviest burdened regions are concentrated in sub‐Saharan Africa, with the highest ASMR and ASDR for breast cancer in southern sub‐Saharan Africa. The highest ASPR was in high‐income North America, at 543.55/100000 (95% UI: 514.63–570.29) (Table S1).

From 1990 to 2021, the changes in ASIR and ASPR in different regions were roughly similar. In general, the ASIR and ASPR in high SDI regions showed a downward trend (EAPC = −0.27 and − 0.12), while the ASIR and ASPR in medium and low SDI regions showed an obvious upward trend, with EAPCs of 2.27 and 2.23, respectively (Table 1). In terms of geographical distribution, the regions with the most obvious increases in ASIR and ASPR were North Africa and the Middle East, with EAPCs of 3.97 and 3.50, respectively (Tables 1 and S1). Global breast cancer ASMR and ASDR showed a downward trend, with the largest decline in high‐income North America, where EAPCs were − 1.86 (95% CI: −1.94 to −1.78) and − 1.94 (95% CI: −2.00 to −1.87), respectively (Tables 1 and S1).

### 3.3 National Level

**FIGURE 1:**
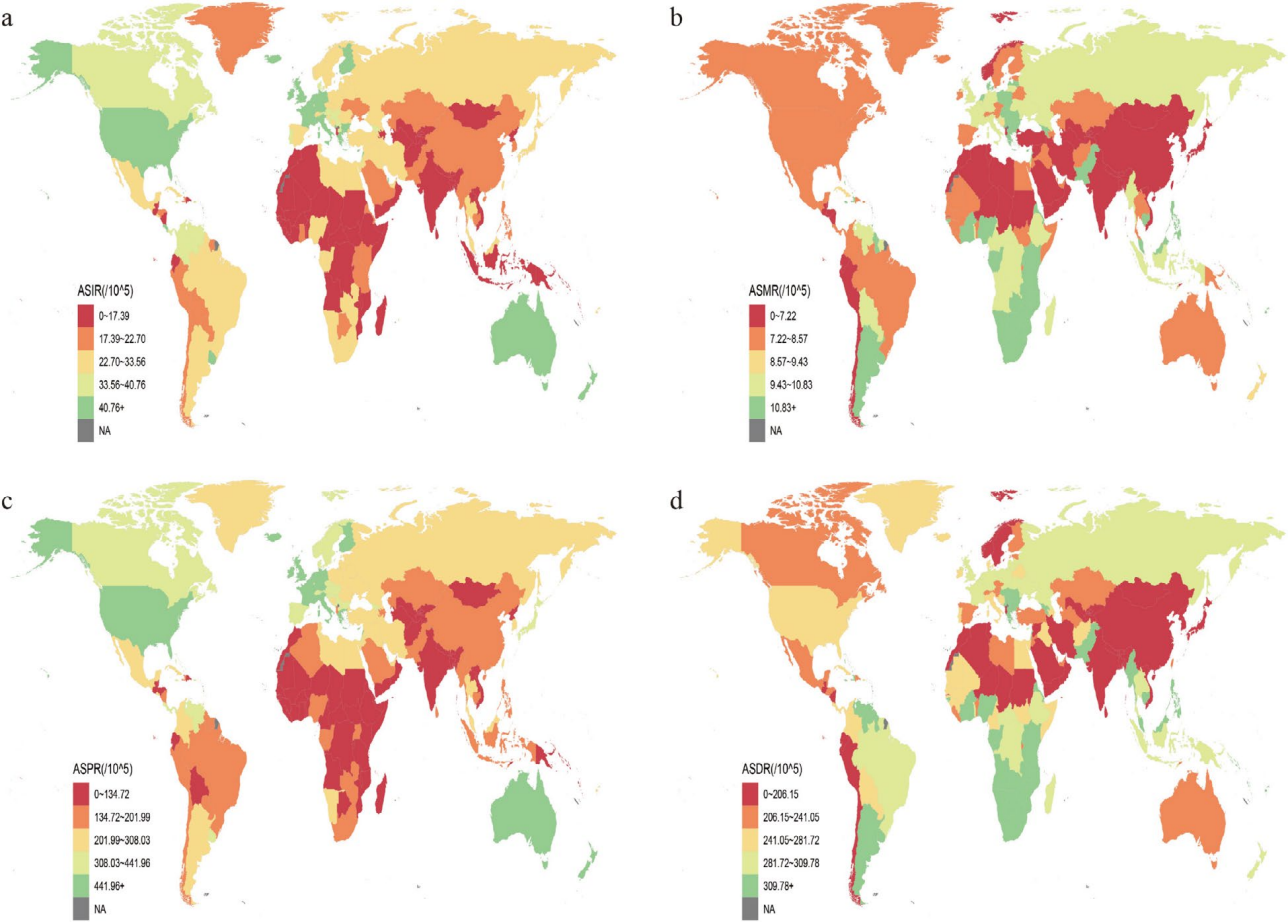
Global burden of breast cancer ASIR, ASMR, ASPR, and ASDR across 204 countries or territories in 2021. (a) ASIR; (b) ASMR; (c) ASPR; (d) ASDR. ASDR: age‐standardized DALYs rate; ASIR: age‐standardized incidence rate; ASMR: age‐standardized mortality rate; ASPR: age‐standardized prevalence rate; DALYs: disability‐adjusted life years.

In 2021, China had the highest number of BC cases globally, with 402 794 cases (95% UI: 312117–505 644), while Monaco recorded the highest ASIR at 86.05/100000 (95% UI: 63.59–116.05) (Figure 1 and Table S2). Palau had the highest ASMR at 21.90/100000 (95% UI: 17.28–27.70) (Figure 1 and Table S3), and Monaco reported the highest ASPR at 886.30/100000 (95% UI: 708.72–1127.15) (Figure 1 and Table S4). The highest ASDR was observed in Nauru at 640.76/100000 (95% UI: 381.85–994.52) (Figure 1 and Table S5).

Between 1990 and 2021, Turkmenistan showed the largest annual increase in ASIR, at 6.77% per year, while Turkey had the most significant ASMR growth, at 3.97% per year (Tables S2 and S4). Turkmenistan also recorded the fastest annual growth in ASPR, at 5.86% per year (Figure S1 and Table S4). Among these countries, 76 nations, including Denmark, experienced a decline in ASDR, whereas 128 countries, such as Turkey, saw a year‐by‐year increase in ASDR (Figure S1 and Table S5).

### 3.4 Age and Gender Patterns in Breast Cancer Burden

**FIGURE 2:**
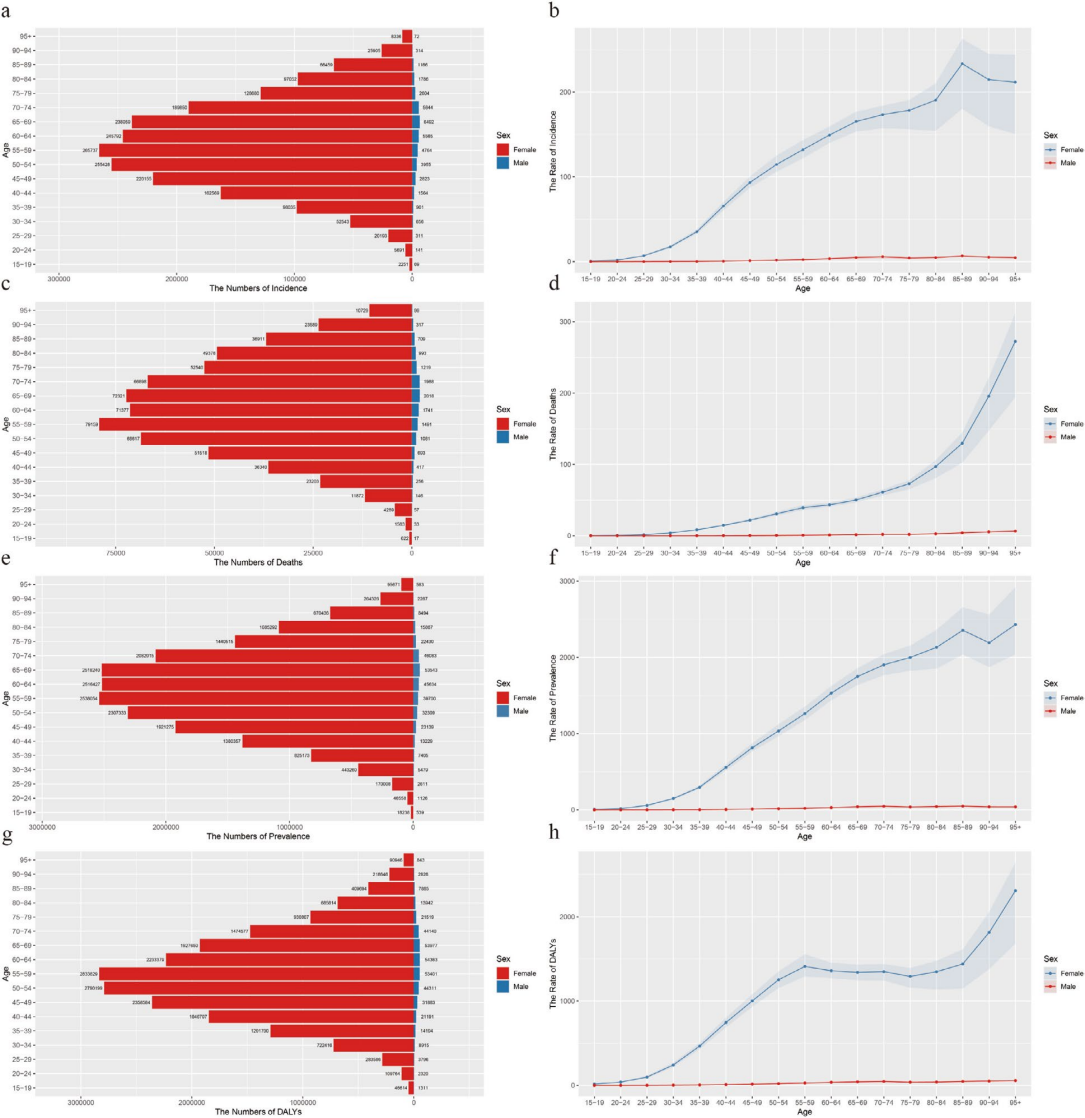
The global burden of breast cancer in different age groups of male and female in 2021. (a) Incidence cases in different age groups; (b) incidence rates in different age groups; (c) mortality cases in different age groups; (d) Mortality rates in different age groups; (e) prevalence cases in different age groups; (f) Prevalence rates in different age groups; (g) DALYs cases in different age groups; (h) DALYs rates in different age groups; DALYs: disability‐adjusted life years.

Breast cancer, the most common cancer among women, shows distinct gender differences. For females, mortality, prevalence, and DALYs were highest in the 95+ age group, while the incidence peaked in the 85–89 age group. The number of new cases increased with age, reaching peaks in the 55–59, 60–64, and 65–69 age groups before declining. Among males, new cases peaked around the age of 70 (Figure 2 and Tables S6–9).

In 2021, the crude incidence and prevalence rates of breast cancer increased with age, peaking in the 85–89 age group. Crude mortality and DALYs also rose with age, though DALYs remained relatively stable between the ages of 55 and 89. The age group with the highest crude incidence, prevalence, and DALYs was younger in males compared to females (Figure 2 and Tables S6–9).

### 3.5 Global Breast Cancer Burden Attributable to Risk Factors

**FIGURE 3:**
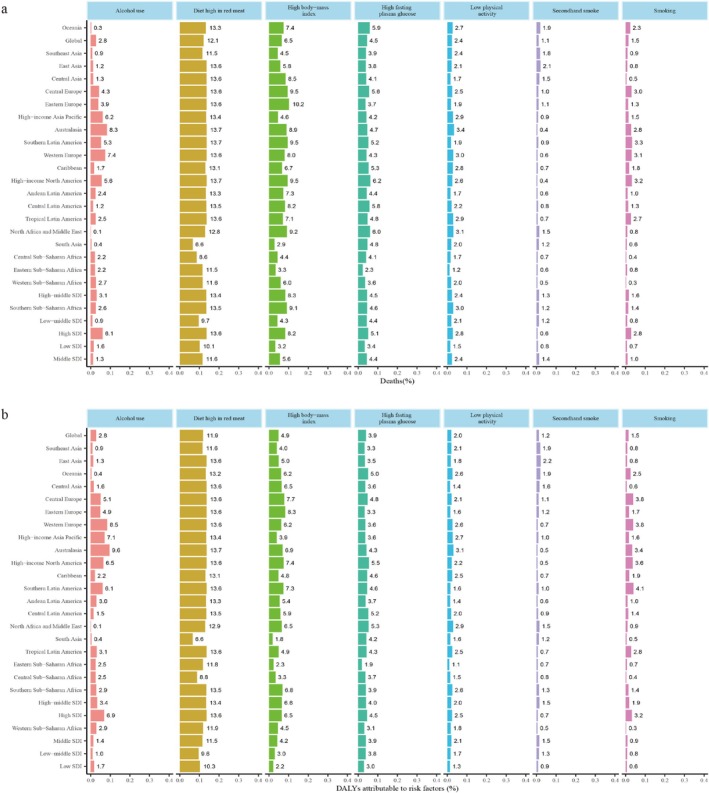
Percentage of deaths and DALYs owing to breast cancer attributable to risk factors for regional and global in 2021. (a) Percentage of deaths; (b) percentage of DALYs. DALYs: disability‐adjusted life years.

In 2021, an estimated 180,000 global deaths from breast cancer were attributed to all risk factors, marking an 86.48% increase compared to approximately 100,000 deaths in 1990. The ASMR decreased from 2.56/100000 (95% UI: 1.00–3.86) in 1990 to 2.14/100000 (95% UI: 0.67–3.31) in 2021 (Figure 3 and Table S10). Among behavioral risk factors, diets rich in red meat accounted for the highest number of deaths. For metabolic risk factors, high BMI was the leading contributor to mortality. Notably, deaths attributable to high fasting plasma glucose showed the largest increase, rising by 181.34% from 1990 to 2021, with its ASMR consistently increasing during this period (Figure 3 and Table S10).

In 2021, nearly 5.2 million DALYs (95% UI: 1685231–8 051 007) were attributed to seven breast cancer risk factors. Behavioral risk factors accounted for 3.77 million DALYs, while metabolic risk factors contributed 1.78 million DALYs (Figure 3 and Table S10). Compared to 1990, high fasting plasma glucose had the fastest DALY growth, increasing by 176.42%. Behavioral risk factors led to a declining ASDR trend (−0.97; 95% CI: −1.02 to −0.93), whereas metabolic risk factors showed an increasing ASDR trend (0.30; 95% CI: 0.27–0.34) (Figure 3 and Table S10).

### 3.6 Breast Cancer Burden Based on SDI

**FIGURE 4:**
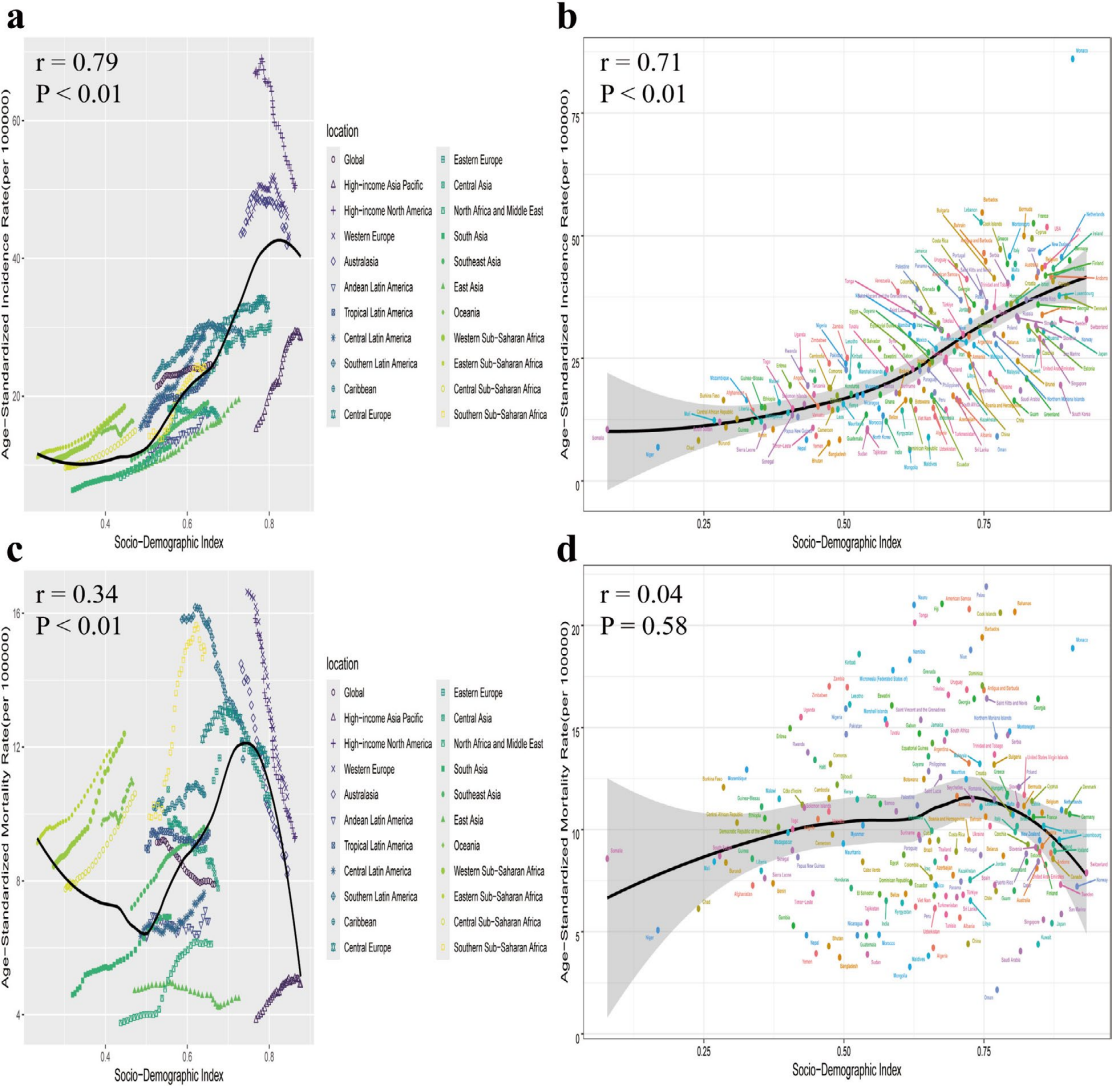
Age‐standardized incidence and mortality for breast cancer across 21 GBD regions and 204 countries and territories by Socio‐demographic Index from 1990 to 2021. (a) ASIR in 21 GBD regions; (b) ASIR in 204 countries; (c) ASMR in 21 GBD regions; (d) ASMR in 204 countries; Each colored line represents annual rates from 1990 to 2021 in a specified region, with expected values based on SDI and disease rates across all locations shown as the black line. ASIR: age‐standardized incidence rate; ASMR: age‐standardized mortality rate; GBD: Global Burden of Disease; SDI: socio‐demographic index.

Across the 21 GBD regions, breast cancer ASIR, ASMR, ASPR, and ASDR were positively correlated with SDI (*r* = 0.79, *r* = 0.34, *r* = 0.81, *r* = 0.34). Overall, global BC ASIR, ASMR, and ASDR in recent years were lower than expected. Among the 21 regions, high‐income North America consistently had higher‐than‐expected ASIR and ASMR, though both showed a declining trend over time. In contrast, high‐income Asia‐Pacific regions reported lower‐than‐expected values but exhibited an increasing trend. In 2021, among 204 countries and territories, ASIR and ASPR showed positive correlations with SDI. When the SDI exceeded 0.75, the relationship between ASMR, ASDR and SDI became negative (Figures 4 and S4).

### 3.7 Decomposition Analysis of Breast Cancer Burden

**FIGURE 5:**
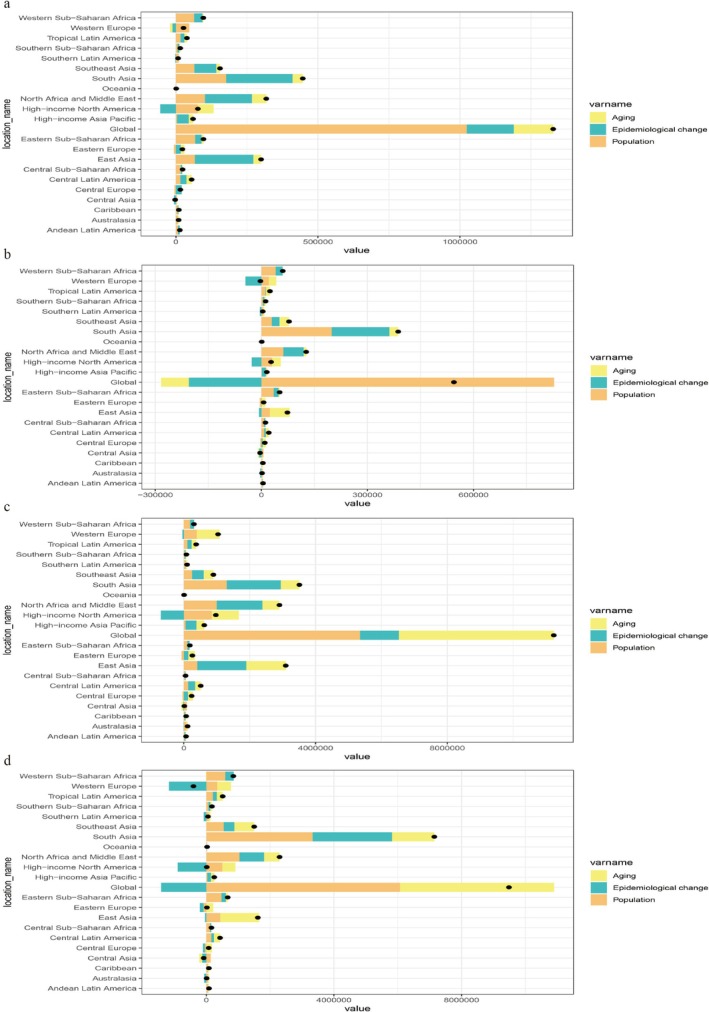
Decomposition analysis of breast cancer from 1990 to 2021. (a) incidence; (b) mortality; (c) prevalence; (d) DALYs. The black dot represents the overall change value of population growth, aging, and epidemiological change. DALYs: disability‐adjusted life years.

A decomposition analysis was conducted to assess the impact of population aging, epidemiological changes, and population growth on global incidence, mortality, prevalence, and DALYs. From 1990 to 2021, population growth emerged as the primary driver of increases in incidence, mortality, prevalence, and DALYs globally. Conversely, epidemiological changes had a negative effect on global mortality and DALYs. Regionally, South Asia recorded the largest increases in incidence, mortality, and DALYs. In this region, epidemiological changes were the main drivers of increases in incidence and prevalence, while population growth was the key contributor to rising mortality and DALYs (Figure 5).

### 3.8 Age‐Standardized Incidence and Mortality Predictions

**FIGURE 6:**
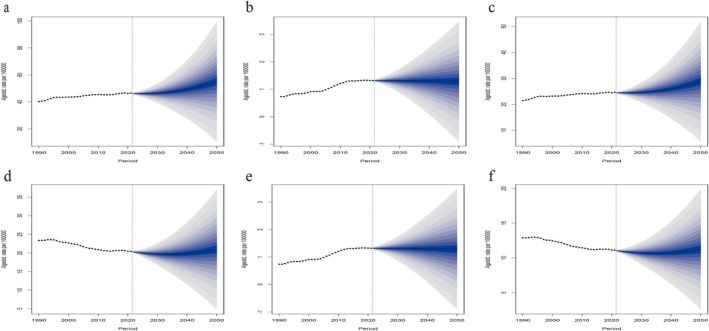
Trends of age‐standardized incidence and mortality of breast cancer for female and male: Actual rates (1990–2021) and predicted rates (2022–2050). (a) ASIR for female; (b) ASIR for male; (c) ASIR for both; (d) ASMR for female; (e) ASMR for male; (f) ASMR for both. The black dots represent the actual rates. The blue region in shows the upper and lower limits of the 95% UI. ASIR: age‐standardized incidence rate; ASMR: age‐standardized mortality rate; UI: uncertainty interval.

The ASIR and ASMR of breast cancer predicted by the BAPC model and their changing trends are shown in Figure 6. Overall, the global ASIR of breast cancer will continue to rise, while the ASMR will decline from 2022 to 2050. By 2050, the global ASIR of breast cancer is expected to rise to 28.79/100000 (95% CI: 5.73–51.86), of which 55.06/100000 (95% CI: 10.68–99.45) for women and 1.28/100000 (95% CI: −0.90‐3.46) for men (Figure 6a–c and Table S11). In addition, the estimated ASMR of breast cancer is predicted to be 11.25/100000 (95% CI: 2.63–19.87), of which 22.99/100000 (95% CI: 4.88–37.1) is for women and 0.41/100000 (95% CI: 0.04–0.78) is for men (Figure 6d–f and Table S11). The results of the ARIMA model were consistent with the above results. The results showed that the global ASIR showed an increasing trend and the ASMR showed a decreasing trend (Figure S5 and Table S12).

## 4 Discussion

This study is based on the latest breast cancer data from the 2021 Global Burden of Disease (GBD) database, comparing global, regional, and national data across different age groups, genders, and attributable risk factors. It is the most comprehensive analysis of breast cancer following the update of the GBD database. The global burden of breast cancer remains substantial, with 2 121 564 new cases and 674 199 deaths in 2021. Between 1990 and 2021, ASIR increased by 0.38% annually, while ASPR increased by 0.33% annually, which is contrary to previous research. In contrast, ASMR and ASDR showed a downward trend, with annual decreases of 0.59% and 0.46% during the same period [9]. Our findings align with previous studies showing rising breast cancer incidence globally, particularly in high SDI regions, while mortality is generally decreasing. However, unlike earlier GBD analyses, our study offers more detailed projections to 2050, indicating an expected rise in incidence. Using the latest GBD 2021 dataset, which incorporates updated demographic and health data, we improve the accuracy of these projections. This study also emphasizes the influence of sociodemographic factors on regional differences, a factor less explored in previous research. The aging global population is a major reason for the rising incidence of breast cancer. As life expectancy increases, more women are entering the high‐risk age group for breast cancer, significantly boosting the number of new cases. Societal development has led to changing dietary habits and lifestyles in many developing countries, such as high‐fat diets, high‐sugar diets, lack of exercise, obesity, and alcohol consumption, all of which increase the risk of breast cancer [18]. Additionally, trends such as later marriages, lower birth rates, reduced breastfeeding duration, and having fewer or no children are also contributing to higher breast cancer risks [19, 20, 21, 22]. Encouragingly, the implementation of national breast cancer screening programs in many countries has improved early diagnosis and survival rates [23]. Furthermore, continuous advancements in breast cancer treatment, including the development of various targeted therapies and immunotherapies, have also improved survival rates. These trends indicate that while the incidence burden of breast cancer is increasing, the burdens of breast cancer‐related deaths and DALYs have been alleviated. These improvements can be attributed to the positive impacts of societal development, technological advancements, industrialization, and enhanced healthcare resources.

Our study results indicate significant regional disparities in the burden of breast cancer. The ASIR in high SDI regions is the highest but shows a downward trend. Conversely, medium and low SDI regions show an increasing trend in ASIR, with the most significant rises observed in North Africa and the Middle East, East Asia, and sub‐Saharan Africa. In economically developed regions, breast cancer screening has been a priority for decades, with high mammography screening rates leading to the detection of more early‐stage cases, though possibly resulting in overdiagnosis of breast cancer [24, 25]. However, with slow population growth and stable screening participation in these regions, incidence has tended to stabilize or decline [26]. However, in some low‐ and middle‐income countries, there may be a socio‐cultural stigma and lack of awareness of breast cancer symptoms that can delay diagnosis and reduce survival rates [1, 2]. However, as population growth and awareness of breast cancer increase, breast cancer incidence rates continue to rise in these areas. Economic disparity also significantly affects access to health care. In low‐ and middle‐income countries, limited financial resources reduce access to diagnostic tools (e.g., mammography) and treatment options, leading to poorer outcomes [4]. High‐income countries allocate more funds to advanced diagnostic and treatment technologies to ensure higher patient survival rates [5]. In contrast, in low SDI regions, with population growth and increased awareness of breast cancer, incidence is expected to rise in the coming years. The ASMR in sub–Saharan Africa is showing the most significant upward trend, highlighting the ongoing challenges in cancer management and access to medical resources [11]. Conversely, high‐income regions, particularly North America, show a significant decline in ASMR. Low‐income regions lack adequate medical facilities, equipment, and specialized personnel, and the absence of early screening capabilities means many people cannot afford expensive treatments and prognoses, further exacerbating the disease burden and mortality [27, 28].

At the national level, China had the highest number of new BC cases in 2021. The countries with the highest ASIR were Monaco. The countries with the highest mortality were Palau. These disparities highlight the impact of national healthcare policies, socio‐economic factors, and access to medical services on breast cancer outcomes [29, 30]. While our analysis differs slightly from previous studies due to the expanded and updated data sources in GBD 2021, the trends remain consistent: low, lower‐middle, and middle SDI regions will increasingly bear a significant burden on individuals and society [9].

In 2021, global breast cancer deaths were attributable to various risk factors, an 88.48% increase compared to 1990. High body mass index and high fasting plasma glucose showed the most significant increases in attributable deaths and DALYs from 1990 to 2021. Additionally, we found that high red meat diets accounted for the largest proportion of attributable deaths and DALYs among global breast cancer risk factors in 2021. Over the past 30 years, while metabolic risk factors have continued to rise, the ASR of other risk factors has been declining. With increased awareness and the promotion of various prevention strategies, common risk factors like smoking and alcohol consumption are becoming more widely recognized for their dangers. However, with social development and improved living standards, metabolic factors are inevitably causing greater harm [31, 32]. These findings underscore the necessity of interventions targeting modifiable risk factors to reduce the burden of breast cancer [33, 34, 35].

Breast cancer remains the most common cancer among women, with significant gender differences. However, the steady rise in age‐standardized incidence (ASIR) and mortality (ASMR) rates for men over the past 30 years should not be ignored. BRCA mutations, radiation exposure, and familial inheritance are key risk factors for male breast cancer [36, 37, 38]. These findings underscore the need for increased awareness and surveillance for male breast cancer, particularly through social media and community outreach [39]. While women have a higher risk due to hormonal factors, sociocultural influences, such as gender roles and health behaviors, also contribute to the breast cancer burden. For instance, studies have shown that adverse lifestyle behaviors, including high alcohol use, low physical activity, and obesity, are associated with an increased risk of female‐specific cancers, including breast cancer [40]. In low‐ and middle‐income regions, barriers to screening and treatment lead to higher mortality rates. According to a study published in Nature, death rates from breast cancer were higher in poorer regions than in wealthier nations due to limited access to early detection and treatment [41]. Interventions like improving access to screening, reducing stigma, and enhancing women's health education are essential to address these disparities. Compared to 1990, breast cancer incidence, mortality, prevalence, and DALYs have increased across all age groups in 2021. Previous studies suggest the highest incidence is in women under 50 [10], though differences may arise from finer age groupings in our research. With global aging trends, the cancer risk among the elderly will continue to rise unless significant breakthroughs in prevention occur [41, 42]. In high‐risk countries, more developed regions, late‐onset breast cancer is more common, with incidence rising rapidly before 50 and then increasing slowly [44].

Based on our projections, by 2050, the ASIR of breast cancer is expected to continue increasing, the ASMR will continue to decline, and the overall disease burden will be somewhat alleviated but will remain one of the heaviest burdens. Our study underscores the ongoing need for comprehensive control strategies to address the increasing incidence of breast cancer, particularly in low‐ and middle‐income regions. Efforts to improve early detection, access to effective treatments, and prevention measures targeting modifiable risk factors are crucial. Additionally, addressing regional disparities and strengthening healthcare infrastructure in high‐burden areas can significantly reduce breast cancer mortality and incidence. Of course, like other studies, this research also has its limitations. First, The GBD database integrates data from various countries and regions, using scientific and standardized methods. However, challenges such as data gaps, timeliness, and quality, particularly in low‐income areas, can lead to biases. These limitations may result in the underestimation or overestimation of disease burden, affecting the accuracy of research findings and, consequently, the formulation of public health policies and resource allocation. In low‐income areas, incomplete and inaccurate data require further verification and additional data to ensure the reliability of the conclusions [45]. Second, the molecular subtypes of breast cancer are numerous and significantly different, but we cannot analyze based on the database data. Thirdly, there are many factors affecting breast cancer, but here we can only analyze based on the risk factors provided by the GBD database. Finally, the unprecedented impact of the COVID‐19 pandemic has not been detailed in our analysis.

The global burden of breast cancer remains significant, with notable regional and national differences. Although advances in early detection and treatment have led to reduced mortality, the rising incidence highlights the need for continued public health efforts. Given the projected rise in breast cancer incidence and mortality, particularly in low‐ and middle‐income countries, targeted public health strategies are essential. Governments should prioritize affordable early detection programs, such as mammography and clinical breast exams, especially in rural and underserved areas. Strengthening healthcare infrastructure through increased investment, healthcare professional training, and expanded access to treatments like chemotherapy, surgery, and radiotherapy is crucial. Public health campaigns should focus on educating populations about modifiable risk factors, such as diet, physical activity, and alcohol consumption, to reduce breast cancer risk. Raising awareness about symptoms and the importance of early detection can help address delays in diagnosis, particularly in regions where cancer stigma often leads to late‐stage diagnoses. Additionally, improving data collection through enhanced cancer registries and reporting systems is vital for more accurate estimates of the disease burden in these regions.

## Author Contributions

**Qiao Huang and Gao‐Song Wu:** conceptualization. **Tong Deng:** data curation. **Tong Deng, Hao Zi, and Qiao Huang:** formal analysis. **Xing‐Pei Guo:** investigation. **Li‐Sha Luo and Qiao Huang:** methodology. **Ya‐Long Yang and Jin‐Xuan Hou:** project administration. **Tong Deng:** software. **Jin‐Xuan Hou and Rui Zhou:** supervision. **Qian‐Qian Yuan and Qing Liu:** validation. **Tong Deng and Gao‐Song Wu:** writing – original draft. **Gao‐Song Wu:** writing – review and editing. The work reported in the article has been performed by the authors, unless clearly specified in the text.

## Ethics Statement

The authors have nothing to report.

## Consent

Informed consent was obtained from all individual participants included in the study.

## Conflicts of Interest

The authors declare no conflicts of interest.

## Supporting information


**Table S1.** Prevalence and DALYs cases for breast cancer in 2021 for both sexes and its corresponding EAPC of age‐standardized rates by Global Burden of Disease (GBD).
**Table S2.** The all‐ages numbers and the age‐standardized rates of incidence, and its corresponding EAPC of breast cancer among 204 countries in 1990 and 2021.
**Table S3.** The all‐ages numbers and the age‐standardized rates of mortality, and its corresponding EAPC of breast cancer among 204 countries in 1990 and 2021.
**Table S4.** The all‐ages numbers and the age‐standardized rates of prevalence, and its corresponding EAPC of breast cancer among 204 countries in 1990 and 2021.
**Table S5.** The all‐ages numbers and the age‐standardized rates of DALYs, and its corresponding EAPC of breast cancer among 204 countries in 1990 and 2021.
**Table S6.** Age structure of global male and female breast cancer incidence in 2021.
**Table S7.** Age structure of global male and female breast cancer mortality in 2021.
**Table S8.** Age structure of global male and female breast cancer prevalence in 2021.
**Table S9.** Age structure of global male and female breast cancer DALYs in 2021.
**Table S10.** Global deaths and DALYs from breast cancer risk factors in 1990 and 2021, with percentage changes.
**Table S11.** BAPC prediction model predicts the global age‐standardized incidence and mortality of breast cancer from 2022 to 2050.
**Table S12.** ARIMA prediction model predicts the global age‐standardized incidence and mortality of breast cancer from 2022 to 2050.
**Figure S1.** Global burden of breast cancer ASIR, ASMR, ASPR, and ASDR across 204 countries or territories in 1990. (a) ASIR; (b) ASMR; (c) ASPR; (d) ASDR. ASIR: age‐standardized incidence rate; ASMR: age‐standardized mortality rate; ASPR: age‐standardized prevalence rate; ASDR: age‐standardized DALYs rate; DALYs: disability‐adjusted life years.
**Figure S2.** Global burden of breast cancer ASIR, ASMR, ASPR, and ASDR across 204 countries or territories in 2021 for female and male. (a) ASIR for female; (b) ASIR for male; (c) ASMR for female; (d) ASMR for male; (e) ASPR for female; (f) ASPR for male; (g) ASDR for female; (h) ASDR for male; ASIR: age‐standardized incidence rate; ASMR: age‐standardized mortality rate; ASPR: age‐standardized prevalence rate; ASDR: age‐standardized DALYs rate; DALYs: disability‐adjusted life years.
**Figure S3.** Global burden of breast cancer ASIR, ASMR, ASPR, and ASDR across 204 countries or territories in 2021 for female and male. (a) ASIR for female; (b) ASMR for female; (c) ASPR for female; (d) ASDR for female; (e) ASIR for male; (f) ASMR for male; (g) ASPR for male; (h) ASDR for male; ASIR: age‐standardized incidence rate; ASMR: age‐standardized mortality rate; ASPR: age‐standardized prevalence rate; ASDR: age‐standardized DALYs rate; DALYs: disability‐adjusted life years.
**Figure S4.** Age‐standardized incidence and prevalence and DALYs for breast cancer across 21 GBD regions and 204 countries and territories by Socio‐demographic Index from 1990 to 2021. (a) ASPR in 21 GBD regions; (b) ASPR in 204 countries; (c) ASDR in 21 GBD regions; (d) ASDR in 204 countries; Each colored line represents annual rates from 1990 to 2021 in a specified region, with expected values based on SDI and disease rates across all locations shown as the black line. SDI: Socio‐demographic Index; ASPR: age‐standardized prevalence rate; ASDR: age‐standardized DALYs rate; DALYs: disability‐adjusted life years; GBD: Global Burden of Disease.
**Figure S5.** Trends of age‐standardized incidence and mortality of breast cancer for female and male: actual rates (1990–2021) and forecast rates (2022–2050). (a) ASIR for female; (b) ASIR for male; (c) ASIR for both; (d) ASMR for female; (e) ASMR for male; (f) ASMR for both. The red line represents the actual rates. The yellow dots represent the forecast rates. The yellow region in shows the upper and lower limits of the 95% UI. ASIR: age‐standardized incidence rate; ASMR: age‐standardized mortality rate; UI: uncertainty interval.

## Data Availability

Data used for the analyses are publicly available from the Institute of Health Metrics and Evaluation (http://www.healthdata.org/; http://ghdx.healthdata.org/gbd‐results‐tool). The datasets used and/or analysed during the current study are available from the corresponding author on reasonable request.
