# Supplementary material for: Global, Regional, and National Burden of Breast Cancer, 1990–2021, and Projections to 2050: A Systematic Analysis of the Global Burden of Disease Study 2021
Source: Thorac Cancer. 2025 May 4;16(9):e70052. doi: 10.1111/1759-7714.70052 (PMC12050159; doi:10.1111/1759-7714.70052)
Supplement: Supplementary file 1 — Table S1. Prevalence and DALYs cases for breast cancer in 2021 for both sexes and its corresponding EAPC of age‐standardized rates by Global Burden of Disease (GBD). Table S2. The all‐ages numbers and the age‐standardized rates of incidence, and its corresponding EAPC of breast cancer among 204 countries in 1990 and 2021. Table S3. The all‐ages numbers and the age‐standardized rates of mortality, and its corresponding EAPC of breast cancer among 204 countries in 1990 and 2021. Table S4. The all‐ages numbers and the age‐standardized rates of prevalence, and its corresponding EAPC of breast cancer among 204 countries in 1990 and 2021. Table S5. The all‐ages numbers and the age‐standardized rates of DALYs, and its corresponding EAPC of breast cancer among 204 countries in 1990 and 2021. Table S6. Age structure of global male and female breast cancer incidence in 2021. Table S7. Age structure of global male and female breast cancer mortality in 2021. Table S8. Age structure of global male and female breast cancer prevalence in 2021. Table S9. Age structure of global male and female breast cancer DALYs in 2021. Table S10. Global deaths and DALYs from breast cancer risk factors in 1990 and 2021, with percentage changes. Table S11. BAPC prediction model predicts the global age‐standardized incidence and mortality of breast cancer from 2022 to 2050. Table S12. ARIMA prediction model predicts the global age‐standardized incidence and mortality of breast cancer from 2022 to 2050. Figure S1. Global burden of breast cancer ASIR, ASMR, ASPR, and ASDR across 204 countries or territories in 1990. (a) ASIR; (b) ASMR; (c) ASPR; (d) ASDR. ASIR: age‐standardized incidence rate; ASMR: age‐standardized mortality rate; ASPR: age‐standardized prevalence rate; ASDR: age‐standardized DALYs rate; DALYs: disability‐adjusted life years. Figure S2. Global burden of breast cancer ASIR, ASMR, ASPR, and ASDR across 204 countries or territories in 2021 for female and male. (a) ASIR for female; (b) [file TCA-16-e70052-s001.docx]

**Global, regional, and national burden of breast cancer, 1990–2021: a systematic analysis for the global burden of disease study 2021**

Tong Deng^1,2^, Hao Zi^3^, Xingpei Guo^4^, Lisha Luo^2^, Yalong Yang^5^, Jinxuan Hou^1^, Rui Zhou^1^, Qianqian Yuan^1^, Qing Liu^6^, Qiao Huang^2*^, and GaoSong Wu^1*^

^1^ Department of Thyroid and Breast Surgery, Zhongnan Hospital of Wuhan University, No. 169 Donghu Road, 430000, Wuhan, China

^2^ Center for Evidence-Based and Translational Medicine, Zhongnan Hospital of Wuhan University, No. 169 Donghu Road, 430000, Wuhan, China

^3^Evidence-Based Medicine Center, Xiangyang No.1 People's Hospital, Hubei University of Medicine, No. 15 Jiefang Road, 441001, Xiangyang, China

^4^Department of General Surgery, Zhengzhou Central Hospital Affiliated to Zhengzhou University, Zhengzhou, No. 195 Tongbai Road, 450000, Zhengzhou, China

^5^Department of breast surgery, Hubei Cancer Hospital, Tongji Medical College, Huazhong University of Science and Technology, Hubei Provincial Clinical Research Center for Breast Cancer, Wuhan Clinical Research Center for Breast Cancer, No.116 Zhuo Daoquan South Road, 430079, Wuhan, China

^6^Department of Physical Examination, Zhengzhou Central Hospital Affiliated to Zhengzhou University, No. 195 Tongbai Road, 450000, Zhengzhou, China

^*^ Corresponding authors: Qiao Huang, mailing address: No. 169, Donghu Road, Wuchang District, Wuhan, 430071, China. (+86) 027 6781 2817, stat_bigdata@163.com;

GaoSong Wu, mailing address: No. 169, Donghu Road, Wuchang District, Wuhan, 430071, China. (+86) 13296687983, 0000-0001-9296-7080,[wugaosong@whu.edu.cn](mailto:wugaosong@whu.edu.cn)

**Supplementary Table**

Table S1. Prevalence and DALYs cases for breast cancer in 2021 for both sexes and its corresponding EAPC of age-standardised rates by Global Burden of Disease (GBD)

Table S2. The all-ages numbers and the age-standardized rates of incidence, and its corresponding EAPC of breast cancer among 204 countries in 1990 and 2021

Table S3. The all-ages numbers and the age-standardized rates of mortality, and its corresponding EAPC of breast cancer among 204 countries in 1990 and 2021

Table S4. The all-ages numbers and the age-standardized rates of prevalence, and its corresponding EAPC of breast cancer among 204 countries in 1990 and 2021

Table S5. The all-ages numbers and the age-standardized rates of DALYs, and its corresponding EAPC of breast cancer among 204 countries in 1990 and 2021

Table S6. Age structure of global male and female breast cancer incidence in 2021

Table S7. Age structure of global male and female breast cancer mortality in 2021

Table S8. Age structure of global male and female breast cancer prevalence in 2021

Table S9. Age structure of global male and female breast cancer DALYs in 2021

Table S10. Global deaths and DALYs from breast cancer risk factors in 1990 and 2021, with percentage changes

Table S11. BAPC prediction model predicts the global age-standardized incidence and mortality of breast cancer from 2022 to 2050

Table S12. ARIMA prediction model predicts the global age-standardized incidence and mortality of breast cancer from 2022 to 2050

**Supplementary figures**

**Figure S1.** Global burden of breast cancer ASIR, ASMR, ASPR, and ASDR across 204 countries or territories in 1990. (a) ASIR; (b) ASMR; (c) ASPR; (d) ASDR. ASIR: age-standardized incidence rate; ASMR: age-standardized mortality rate; ASPR: age-standardized prevalence rate; ASDR: age-standardized DALYs rate; DALYs: disability-adjusted life years.

**Figure S2.** Global burden of breast cancer ASIR, ASMR, ASPR, and ASDR across 204 countries or territories in 2021 for female and male. (a) ASIR for female; (b) ASIR for male; (c) ASMR for female; (d) ASMR for male; (e) ASPR for female; (f) ASPR for male; (g) ASDR for female; (h) ASDR for male; ASIR: age-standardized incidence rate; ASMR: age-standardized mortality rate; ASPR: age-standardized prevalence rate; ASDR: age-standardized DALYs rate; DALYs: disability-adjusted life years.

**Figure S3.** Global burden of breast cancer ASIR, ASMR, ASPR, and ASDR across 204 countries or territories in 2021 for female and male. (a) ASIR for female; (b) ASMR for female; (c) ASPR for female; (d) ASDR for female; (e) ASIR for male; (f) ASMR for male; (g) ASPR for male; (h) ASDR for male; ASIR: age-standardized incidence rate; ASMR: age-standardized mortality rate; ASPR: age-standardized prevalence rate; ASDR: age-standardized DALYs rate; DALYs: disability-adjusted life years.

**Figure S4.** Age-standardized incidence and prevalence and DALYs for breast cancer across 21 GBD regions and 204 countries and territories by Socio-demographic Index from 1990 to 2021. (a) ASPR in 21 GBD regions; (b) ASPR in 204 countries; (c) ASDR in 21 GBD regions; (d) ASDR in 204 countries; Each colored line represents annual rates from 1990 to 2021 in a specified region, with expected values based on SDI and disease rates across all locations shown as the black line. SDI: Socio-demographic Index; ASPR: age-standardized prevalence rate; ASDR: age-standardized DALYs rate; DALYs: disability-adjusted life years; GBD: Global Burden of Disease.

**Figure S5.** Trends of age-standardized incidence and mortality of breast cancer for female and male: actual rates (1990–2021) and forecast rates (2022–2050). (a) ASIR for female; (b) ASIR for male; (c) ASIR for both; (d) ASMR for female; (e) ASMR for male; (f) ASMR for both. The red line represents the actual rates. The yellow dots represent the forecast rates. The yellow region in shows the upper and lower limits of the 95% UI. ASIR: age-standardized incidence rate; ASMR: age-standardized mortality rate; UI: uncertainty interval.

Table S1. Prevalence and DALYs cases for breast cancer in 2021 for both sexes and its corresponding EAPC of age-standardised rates by Global Burden of Disease.

| Characteristics | Counts  (2021) | Age-standardized Prevalence rate (per 100,000) (95% UI) | |  | EAPC (95% CI)  1990–2021 |  | Counts  (2021) | Age-standardized DALYs rate (per 100,000) (95% UI) | |  | EAPC (95% CI)  1990–2021 |
| --- | --- | --- | --- | --- | --- | --- | --- | --- | --- | --- | --- |
|  |  | 1990 | 2021 |  |  |  |  | 1990 | 2021 |  |  |
| Global | 20643638(19562590-21799703) | 218.44(203.00-237.82**)** | 238.86(226.17-252.24**)** |  | 0.33(0.31-0.35) |  | 20635718(19358111-21993503**)** | 265.03(250.31-279.67**)** | 239.03(224.20-254.88**)** |  | -0.46(-0.51--0.40) |
| Female | 20323179(19248044-21451412) | 404.54(377.02-439.95) | 450.64(427.02-475.96**)** |  | 0.38(0.36-0.40) |  | 20254802(18963376-21574429**)** | 503.81(475.91-532.23**)** | 455.56(426.64-485.30**)** |  | -0.46(-0.52--0.40) |
| Male | 320459(220533-384317) | 4.26(3.81-4.76) | 7.75(5.41-9.24**)** |  | 2.30(2.14-2.46) |  | 380917(252900-476417**)** | 7.39(6.23-8.83**)** | 9.16(6.12-11.42**)** |  | 0.85(0.75-0.95) |
| Low SDI | 575477(512773-637763**)** | 59.55(52.38-68.77**)** | 91.33(82.05-100.57**)** |  | 1.41(1.23-1.59) |  | 1677080(1468417-1891533**)** | 199.37(170.46-232.14**)** | 258.71(228.42-292.99**)** |  | 0.78(0.66-0.90) |
| Low-middle SDI | 1907724(1752622-2048800**)** | 60.77(55.07-68.50**)** | 116.76(107.42-125.57**)** |  | 2.23(2.14-2.32) |  | 4135798(3730357-4539759**)** | 166.11(148.37-188.98**)** | 248.84(224.37-272.93**)** |  | 1.31(1.27-1.36) |
| Middle SDI | 4891405(476818-5364127**)** | 93.17(85.21-102.80**)** | 172.33(157.93-188.88**)** |  | 2.08(2.01-2.15) |  | 6169416(5577685-6827006**)** | 188.07(172.55-206.71**)** | 216.97(196.42-240.17**)** |  | 0.33(0.28-0.39) |
| High-middle SDI | 5135356(4733252-5632081**)** | 216.86(200.24-237.99**)** | 433.68(413.83-451.67**)** |  | 0.78(0.73-0.84) |  | 4262982(3900200-4726495**)** | 288.35(272.25-303.78**)** | 225.40(206.14-249.94**)** |  | -0.98(-1.07--0.89) |
| High SDI | 8113345(7670871-8493246**)** | 456.40(425.78 492.41**)** | 268.27(247.13-294.80**)** |  | -0.12(-0.21--0.04) |  | 4367550(4024277-4651990**)** | 387.28(372.21-401.58**)** | 242.88(226.31-257.07**)** |  | -1.59(-1.63--1.55) |
| Central Europe, eastern Europe, and central Asia | 1707274(1605907-1814487**)** | 230.30(213.98-251.49**)** | 274.72(258.21-292.34**)** |  | 0.55(0.49-0.60) |  | 1819469(1682867-1970621**)** | 349.40(340.29-359.32**)** | 298.30(275.85-323.45**)** |  | -0.81(-0.98--0.64) |
| Central Asia | 141234(128794-154430**)** | 174.38(161.69-187.73**)** | 156.25(143.31-169.89**)** |  | -0.24(-0.29--0.18) |  | 219259(194881-246457**)** | 340.59(321.52-358.40**)** | 236.07(210.60-264.05**)** |  | -1.01(-1.09--0.92) |
| Central Europe | 621426(581968-661846**)** | 238.39(221.22-261.16**)** | 311.89(291.51-333.07**)** |  | 0.99(0.88-1.10) |  | 609778(562490-659711**)** | 355.66(340.84-371.30**)** | 308.58(284.00-335.34**)** |  | -0.61(-0.72--0.51) |
| Eastern Europe | 944614(861346-1028372**)** | 235.65(219.18-258.23**)** | 283.78(258.01-310.11**)** |  | 0.47(0.39-0.55) |  | 990433(884267-1123187**)** | 347.42(336.28-358.59**)** | 304.43(270.91-346.39**)** |  | -0.88(-1.11--0.66) |
| High-income | 8701146(8226464-9136854**)** | 479.86(447.61-519.25**)** | 455.17(435.80-474.02**)** |  | -0.12(-0.21--0.03) |  | 4640614(4265442-4938832**)** | 410.25(394.25-425.33**)** | 253.55(236.71-268.09**)** |  | -1.62(-1.66--1.58) |
| Australasia | 223891(205321-241788**)** | 471.22(434.21-516.49**)** | 465.32(427.93-503.56**)** |  | 0.08(-0.05-0.21) |  | 113411(101204-126350**)** | 422.79(399.84-445.11**)** | 244.59(220.61-271.20**)** |  | -1.85(-1.90--1.80) |
| High-income Asia Pacific | 1151787(1053383-1232465**)** | 201.78(182.52-225.31**)** | 319.44(296.44-339.61**)** |  | 1.80(1.67-1.93) |  | 542175(483247-588128**)** | 137.49(131.61-143.88**)** | 163.11(149.62-175.49**)** |  | 0.54(0.38-0.70) |
| High-income North America | 3285243(3093192-3457738**)** | 680.39(639.49-725.19**)** | 543.55(514.63-570.29**)** |  | -0.88(-0.97--0.79) |  | 1573540(1465781-1678355**)** | 479.56(457.62-500.68**)** | 273.49(256.25-290.02**)** |  | -1.94(-2.00--1.87) |
| Southern Latin America | 222099(208273-236256**)** | 240.72(221.64-265.40**)** | 264.28(248.10-280.74**)** |  | 0.45(0.34-0.55) |  | 263673(242741-283346**)** | 441.07(418.20-461.24**)** | 317.67(292.68-341.54**)** |  | -1.01(-1.11--0.92) |
| Western Europe | 3818125(3592823-4001091**)** | 482.33(447.37-526.16**)** | 476.46(453.30-496.45**)** |  | 0.10(-0.03-0.24) |  | 2147815(1938628-2312416**)** | 472.03(454.04-488.72**)** | 274.25(252.53-293.45**)** |  | -1.81(-1.86--1.76) |
| Latin America and Caribbean | 1390973(1284982-1510846**)** | 127.75(121.87-134.17**)** | 216.80(200.33-235.37**)** |  | 1.61(1.55-1.67) |  | 1691734(1562334-1836822 | 237.91(230.29-246.50**)** | 263.22(242.89-285.78**)** |  | 0.16(0.10-0.21) |
| Andean Latin America | 89325(71777-110048**)** | 77.93(67.89-89.18**)** | 143.22(115.33-176.36**)** |  | 1.95(1.84-2.05) |  | 129549(102648-163266**)** | 195.24(166.79-227.87**)** | 206.64(163.89-259.54**)** |  | -0.05(-0.17-0.07) |
| Caribbean | 127499(111507-143817**)** | 186.20(174.11-199.40**)** | 239.12(208.82-270.17**)** |  | 0.92(0.84-1.00) |  | 166676(139163-197643 | 304.94(278.31-337.12**)** | 314.12(261.83-372.85**)** |  | 0.17(0.12-0.23) |
| Central Latin America | 654422(576794-734015**)** | 126.42(120.13-132.98**)** | 248.95(219.71-278.94**)** |  | 2.09(2.01-2.17) |  | 636255(550996-717861**)** | 195.63(190.02-201.65**)** | 241.70(209.72-272.68**)** |  | 0.53(0.44-0.63) |
| Tropical Latin America | 519728(491298-547381**)** | 123.93(117.88-130.82**)** | 197.09(186.10-207.54**)** |  | 1.32(1.25-1.40) |  | 759254(713099-797995**)** | 268.47(258.30-278.29**)** | 287.76(269.91-302.59**)** |  | -0.01(-0.08-0.06) |
| North Africa and Middle East | 1184781(1075661-1306097**)** | 90.83(80.74-103.22**)** | 221.39(201.53-242.41**)** |  | 3.50(3.32-3.68) |  | 1083820(944244-1245185**)** | 124.15(110.76-142.33**)** | 194.85(170.82-223.46**)** |  | 1.85(1.71-1.98) |
| South Asia | 1654319(1471628-1860382**)** | 50.31(45.43-56.33**)** | 99.07(88.43-111.43**)** |  | 2.23(2.12-2.33) |  | 3781141(3308591-4332369**)** | 152.43(135.30-172.16**)** | 223.52(195.17-255.88**)** |  | 1.16(1.08-1.24) |
| Southeast Asia, east Asia, and Oceania | 5251350(4550425-6123624**)** | 90.05(78.49-104.03**)** | 180.14(156.39-209.54**)** |  | 2.47(2.38-2.55) |  | 5479715(4699431-6354842**)** | 171.80(146.41-201.66**)** | 189.57(162.59-219.88**)** |  | 0.16(0.09-0.23) |
| East Asia | 4047969(3326766-4890732**)** | 89.91(76.93-105.05 | 186.55(153.14-226.45**)** |  | 2.62(2.51-2.73) |  | 3187711(2516808-4001378**)** | 152.23(125.18-184.05**)** | 148.69(117.24-186.75**)** |  | -0.30(-0.39--0.21) |
| Oceania | 12396(10413-15080**)** | 116.47(98.54-139.57**)** | 127.90(109.66-152.25**)** |  | 0.18(0.07-0.28) |  | 36209(29094-46122**)** | 310.08(244.28-391.00**)** | 352.82(289.38-436.76**)** |  | 0.40(0.32-0.49) |
| Southeast Asia | 1190985(1032899-1390419**)** | 90.19(78.70-105.62**)** | 162.31(141.45-188.60**)** |  | 1.99(1.97-2.02) |  | 2255796(1865830-2764871**)** | 234.04(195.31-283.35**)** | 302.57(250.69-369.35**)** |  | 0.78(0.70-0.86) |
| Sub-Saharan Africa | 753794(647600-857603**)** | 78.54(71.34-87.49**)** | 127.42(111.57-143.46**)** |  | 1.70(1.53-1.86) |  | 2139224(1809113-2484563**)** | 257.02(227.86-288.06**)** | 351.00(301.64-403.14**)** |  | 1.08(1.00-1.16) |
| Central sub- Sharan Africa | 73639(57284-93439**)** | 68.48(53.11-86.77**)** | 105.15(83.18-131.80**)** |  | 1.45(1.21-1.68) |  | 225987(167663-298098**)** | 235.74(170.69-313.48**)** | 309.51(231.84-406.91**)** |  | 0.90(0.75-1.06) |
| Eastern sub- Sharan Africa | 252224(214878-295496**)** | 75.24(64.44-88.19**)** | 116.33(101.52-132.62**)** |  | 1.39(1.24-1.55) |  | 749842(627813-899054**)** | 268.21(224.59-324.80**)** | 335.92(286.45-392.21**)** |  | 0.62(0.52-0.73) |
| Southern sub- Sharan Africa | 115140(104874-126455**)** | 111.83(95.10-126.33**)** | 177.55(162.95-193.23**)** |  | 1.99(1.78-2.19) |  | 271585(242599-303051**)** | 286.58(239.94-333.66**)** | 412.60(370.93-457.52**)** |  | 1.72(1.44-2.00) |
| Western sub- Saharan Africa | 312792(241362-402620**)** | 72.88(62.45-83.78**)** | 128.47(102.95-161.08**)** |  | 1.96(1.76-2.15) |  | 891810(669025-1159840**)** | 241.45(197.66-286.13**)** | 356.64(276.51-455.53**)** |  | 1.35(1.21-1.48) |

DALYs: disability-adjusted life years; SDI: socio-demographic index; EAPC: estimated annual percentage change; UI: uncertainty interval; CI: confidence interval;

Table S2. The all-ages numbers and the age-standardized rates of incidence, and its corresponding EAPC of breast cancer among 204 countries in 1990 and 2021

| Characteristics | Counts (2021) | | | Age-standardized incidence rate (per 100,000) (95% UI) | | | | | | EAPC (95% CI)1990–2021 | | |
| --- | --- | --- | --- | --- | --- | --- | --- | --- | --- | --- | --- | --- |
|  |  |  |  | 1990 | | | 2021 | | |  |  |  |
|  | val | upper | lower | val | upper | lower | val | upper | lower | val | upper | lower |
| Afghanistan | 2043.43 | 3724.69 | 1005.98 | 7.25 | 12.38 | 3.76 | 14.29 | 24.59 | 7.74 | 2.37 | 2.52 | 2.21 |
| Albania | 620.03 | 829.94 | 438.42 | 8.77 | 11.18 | 6.89 | 16.26 | 21.82 | 11.60 | 2.64 | 2.97 | 2.31 |
| Algeria | 6065.40 | 8053.74 | 4460.10 | 7.71 | 9.93 | 5.93 | 14.88 | 19.49 | 11.15 | 2.30 | 2.42 | 2.18 |
| American Samoa | 17.47 | 21.78 | 13.70 | 22.08 | 26.45 | 18.32 | 34.86 | 43.65 | 27.34 | 1.60 | 1.70 | 1.49 |
| Andorra | 61.15 | 84.92 | 40.97 | 38.07 | 54.44 | 26.54 | 41.75 | 58.13 | 27.89 | 0.70 | 0.97 | 0.44 |
| Angola | 2291.13 | 3134.62 | 1563.43 | 8.33 | 11.24 | 6.03 | 15.20 | 20.37 | 10.53 | 2.03 | 2.15 | 1.92 |
| Antigua and Barbuda | 51.45 | 55.96 | 47.65 | 32.10 | 35.48 | 28.64 | 46.45 | 50.39 | 43.13 | 1.39 | 1.57 | 1.22 |
| Argentina | 15948.80 | 17342.20 | 14609.41 | 28.18 | 29.94 | 26.40 | 29.49 | 32.06 | 27.01 | 0.15 | 0.34 | -0.05 |
| Armenia | 1170.45 | 1344.38 | 1004.16 | 32.05 | 34.39 | 29.89 | 27.56 | 31.58 | 23.73 | -0.43 | -0.11 | -0.74 |
| Australia | 16366.47 | 18461.42 | 14402.82 | 40.84 | 43.20 | 38.60 | 41.54 | 46.62 | 36.81 | -0.03 | 0.18 | -0.23 |
| Austria | 5296.47 | 5834.58 | 4717.89 | 43.14 | 46.12 | 39.92 | 33.88 | 37.08 | 30.55 | -0.69 | -0.56 | -0.83 |
| Azerbaijan | 2063.93 | 2680.31 | 1506.75 | 16.83 | 19.20 | 13.93 | 17.38 | 22.44 | 12.88 | 0.27 | 0.49 | 0.05 |
| Bahamas | 230.76 | 285.19 | 185.88 | 41.44 | 45.28 | 37.83 | 52.82 | 64.99 | 42.74 | 1.10 | 1.27 | 0.93 |
| Bahrain | 531.03 | 681.88 | 412.73 | 25.30 | 29.73 | 21.64 | 44.81 | 57.52 | 34.69 | 1.69 | 1.94 | 1.45 |
| Bangladesh | 13210.87 | 16952.96 | 9919.09 | 4.02 | 5.43 | 3.01 | 8.36 | 10.74 | 6.27 | 2.25 | 2.43 | 2.07 |
| Barbados | 259.64 | 326.59 | 205.51 | 40.91 | 44.36 | 37.30 | 54.72 | 68.93 | 43.19 | 1.34 | 1.57 | 1.12 |
| Belarus | 4445.58 | 5569.80 | 3472.80 | 23.64 | 26.34 | 21.46 | 29.74 | 37.38 | 23.19 | -0.02 | 0.22 | -0.26 |
| Belgium | 8666.36 | 9577.29 | 7604.98 | 55.28 | 59.33 | 51.18 | 43.41 | 47.61 | 38.92 | -0.82 | -0.59 | -1.04 |
| Belize | 57.16 | 65.32 | 49.78 | 9.57 | 10.50 | 8.64 | 16.48 | 18.77 | 14.40 | 1.83 | 2.06 | 1.60 |
| Benin | 644.90 | 868.72 | 459.02 | 7.67 | 9.12 | 6.21 | 10.51 | 13.81 | 7.73 | 0.95 | 1.03 | 0.87 |
| Bermuda | 59.31 | 76.06 | 48.36 | 52.89 | 58.59 | 47.34 | 49.96 | 63.97 | 40.43 | -0.62 | -0.44 | -0.79 |
| Bhutan | 52.08 | 72.84 | 36.24 | 4.91 | 6.47 | 3.48 | 7.72 | 10.78 | 5.43 | 1.43 | 1.58 | 1.29 |
| Bolivia | 1772.57 | 2585.48 | 1162.21 | 11.73 | 16.91 | 7.71 | 17.86 | 26.05 | 11.71 | 1.25 | 1.30 | 1.20 |
| Bosnia and Herzegovina | 1468.67 | 1825.28 | 1143.16 | 15.24 | 17.19 | 13.12 | 25.73 | 32.25 | 19.82 | 2.36 | 2.66 | 2.05 |
| Botswana | 313.18 | 451.17 | 215.27 | 12.64 | 17.38 | 8.74 | 18.56 | 25.52 | 13.67 | 1.75 | 2.11 | 1.39 |
| Brazil | 61092.71 | 64461.05 | 57179.68 | 16.00 | 16.61 | 15.31 | 23.79 | 25.13 | 22.26 | 1.00 | 1.11 | 0.90 |
| Brunei Darussalam | 111.49 | 138.33 | 86.19 | 15.71 | 20.77 | 11.79 | 24.43 | 30.30 | 18.96 | 2.01 | 2.18 | 1.84 |
| Bulgaria | 5071.09 | 6087.50 | 4090.75 | 28.89 | 32.95 | 25.59 | 41.37 | 49.87 | 33.48 | 1.35 | 1.47 | 1.23 |
| Burkina Faso | 1794.50 | 2409.60 | 1260.02 | 13.39 | 16.87 | 10.27 | 16.48 | 21.62 | 12.05 | 0.70 | 0.78 | 0.61 |
| Burundi | 650.21 | 877.24 | 475.18 | 11.83 | 16.50 | 8.52 | 10.92 | 14.74 | 8.18 | -0.60 | -0.42 | -0.78 |
| Cabo Verde | 74.13 | 96.43 | 56.60 | 12.72 | 15.60 | 10.17 | 15.35 | 19.96 | 11.80 | 0.98 | 1.31 | 0.65 |
| Cambodia | 2672.96 | 3591.60 | 1917.26 | 9.78 | 14.75 | 6.30 | 18.98 | 25.17 | 13.71 | 2.18 | 2.22 | 2.14 |
| Cameroon | 2235.40 | 3125.60 | 1582.47 | 10.20 | 12.60 | 8.17 | 14.41 | 19.76 | 10.45 | 1.03 | 1.15 | 0.92 |
| Canada | 25610.49 | 28545.24 | 22765.25 | 53.65 | 58.11 | 49.14 | 40.76 | 45.01 | 36.74 | -0.96 | -0.83 | -1.09 |
| Central African Republic | 346.75 | 490.06 | 232.37 | 10.42 | 13.60 | 7.73 | 12.63 | 16.74 | 8.92 | 0.56 | 0.61 | 0.51 |
| Chad | 584.58 | 780.12 | 410.00 | 6.16 | 8.04 | 4.42 | 8.25 | 10.82 | 6.01 | 1.01 | 1.05 | 0.97 |
| Chile | 4987.21 | 5514.16 | 4480.41 | 16.73 | 17.86 | 15.59 | 20.33 | 22.46 | 18.32 | 0.91 | 1.02 | 0.80 |
| China | 402794.18 | 505644.32 | 312117.30 | 9.08 | 11.02 | 7.41 | 19.36 | 24.30 | 15.00 | 2.50 | 2.58 | 2.42 |
| Colombia | 19094.81 | 22988.12 | 15798.50 | 18.71 | 20.09 | 17.32 | 34.75 | 41.84 | 28.75 | 1.95 | 2.14 | 1.76 |
| Comoros | 103.71 | 137.99 | 77.45 | 11.98 | 16.15 | 8.72 | 18.89 | 25.15 | 14.17 | 1.28 | 1.37 | 1.18 |
| Congo | 807.38 | 1290.76 | 474.34 | 15.61 | 24.32 | 9.53 | 23.07 | 35.76 | 14.30 | 1.20 | 1.33 | 1.06 |
| Cook Islands | 11.07 | 14.44 | 8.30 | 34.44 | 44.07 | 26.78 | 47.22 | 61.62 | 35.02 | 1.01 | 1.17 | 0.85 |
| Costa Rica | 2402.06 | 2745.30 | 2081.55 | 22.43 | 24.42 | 20.53 | 43.77 | 49.96 | 37.98 | 2.23 | 2.38 | 2.07 |
| Croatia | 2798.59 | 3222.55 | 2359.95 | 33.32 | 37.59 | 29.31 | 36.09 | 41.78 | 30.17 | 0.36 | 0.55 | 0.17 |
| Cuba | 5885.31 | 7002.92 | 4988.88 | 26.21 | 28.05 | 24.32 | 32.13 | 38.32 | 27.19 | 0.66 | 0.76 | 0.55 |
| Cyprus | 957.14 | 1158.06 | 781.72 | 33.25 | 40.27 | 27.26 | 49.42 | 59.63 | 40.10 | 1.74 | 2.10 | 1.38 |
| Czechia | 5949.54 | 7034.94 | 4945.04 | 31.86 | 35.35 | 28.59 | 31.53 | 37.43 | 26.09 | -0.45 | -0.14 | -0.75 |
| Côte d'Ivoire | 2319.00 | 3146.15 | 1640.43 | 10.74 | 13.51 | 8.56 | 16.68 | 22.25 | 12.29 | 1.53 | 1.60 | 1.46 |
| Democratic People's Republic of Korea | 4536.36 | 6257.92 | 3073.77 | 9.22 | 13.28 | 6.10 | 13.30 | 18.16 | 9.14 | 1.43 | 1.54 | 1.32 |
| Democratic Republic of the Congo | 6205.87 | 8484.63 | 4588.15 | 9.23 | 12.49 | 6.58 | 14.02 | 19.04 | 10.35 | 1.37 | 1.63 | 1.12 |
| Denmark | 3860.95 | 4269.69 | 3447.02 | 51.90 | 54.68 | 49.09 | 37.59 | 41.32 | 33.98 | -0.81 | -0.53 | -1.08 |
| Djibouti | 138.82 | 206.78 | 92.27 | 12.49 | 16.49 | 9.33 | 17.40 | 24.69 | 12.07 | 1.03 | 1.08 | 0.98 |
| Dominica | 28.19 | 35.68 | 21.48 | 29.99 | 34.44 | 25.60 | 34.46 | 43.29 | 26.41 | 0.46 | 0.53 | 0.39 |
| Dominican Republic | 1714.85 | 2222.13 | 1292.78 | 10.74 | 12.59 | 9.06 | 16.36 | 21.17 | 12.39 | 1.59 | 1.83 | 1.35 |
| Ecuador | 2765.76 | 3498.61 | 2084.93 | 7.80 | 8.30 | 7.26 | 16.37 | 20.69 | 12.37 | 2.46 | 2.73 | 2.19 |
| Egypt | 20680.03 | 25725.15 | 16203.59 | 8.50 | 9.98 | 7.38 | 27.30 | 33.75 | 21.98 | 4.13 | 4.53 | 3.74 |
| El Salvador | 1636.70 | 2072.37 | 1276.21 | 9.51 | 10.88 | 8.23 | 26.98 | 34.17 | 21.00 | 3.45 | 3.74 | 3.16 |
| Equatorial Guinea | 162.97 | 262.31 | 93.49 | 9.93 | 13.98 | 6.79 | 24.27 | 37.62 | 14.54 | 3.19 | 3.33 | 3.05 |
| Eritrea | 658.47 | 908.72 | 457.62 | 12.97 | 17.29 | 9.76 | 19.50 | 26.24 | 13.73 | 1.30 | 1.40 | 1.21 |
| Estonia | 734.92 | 870.85 | 597.19 | 29.62 | 32.59 | 26.96 | 30.86 | 36.51 | 25.16 | 0.09 | 0.21 | -0.02 |
| Eswatini | 146.43 | 231.13 | 81.95 | 12.78 | 16.30 | 9.67 | 22.95 | 35.28 | 13.69 | 2.11 | 2.46 | 1.76 |
| Ethiopia | 7869.31 | 9705.93 | 6358.33 | 11.75 | 16.89 | 8.04 | 14.95 | 18.33 | 12.29 | 0.63 | 0.85 | 0.42 |
| Fiji | 250.54 | 328.66 | 181.66 | 25.71 | 32.18 | 20.32 | 30.73 | 39.57 | 22.71 | 0.49 | 0.64 | 0.34 |
| Finland | 4276.42 | 4750.81 | 3748.82 | 40.41 | 42.89 | 37.76 | 41.86 | 46.34 | 37.20 | 0.28 | 0.52 | 0.04 |
| France | 59205.37 | 65570.51 | 52391.66 | 43.51 | 46.30 | 40.68 | 52.55 | 57.80 | 46.95 | 0.79 | 0.99 | 0.58 |
| Gabon | 282.57 | 392.90 | 190.92 | 15.17 | 19.97 | 10.89 | 23.49 | 32.53 | 16.26 | 1.23 | 1.34 | 1.12 |
| Gambia | 92.68 | 125.02 | 66.56 | 4.15 | 5.43 | 3.11 | 8.02 | 10.59 | 5.86 | 1.96 | 2.16 | 1.76 |
| Georgia | 1963.39 | 2273.78 | 1683.74 | 33.21 | 36.53 | 29.65 | 35.95 | 41.83 | 30.90 | 0.40 | 0.69 | 0.12 |
| Germany | 73076.59 | 79851.30 | 65325.53 | 42.08 | 44.64 | 39.39 | 44.96 | 48.63 | 40.98 | 0.22 | 0.41 | 0.02 |
| Ghana | 3584.50 | 4794.53 | 2641.86 | 12.18 | 15.49 | 9.41 | 17.57 | 23.22 | 13.25 | 0.99 | 1.08 | 0.91 |
| Greece | 8771.19 | 9580.24 | 7767.48 | 43.33 | 46.07 | 40.43 | 44.28 | 48.41 | 40.19 | -0.22 | -0.06 | -0.37 |
| Greenland | 15.17 | 20.00 | 11.07 | 28.20 | 35.19 | 22.77 | 20.87 | 27.19 | 15.55 | -1.15 | -1.02 | -1.28 |
| Grenada | 45.06 | 51.67 | 38.81 | 27.97 | 31.11 | 25.19 | 39.25 | 44.94 | 33.99 | 1.28 | 1.51 | 1.05 |
| Guam | 31.80 | 38.50 | 26.48 | 17.16 | 19.90 | 14.75 | 16.39 | 19.68 | 13.64 | 0.32 | 0.58 | 0.07 |
| Guatemala | 1642.40 | 1950.53 | 1375.57 | 5.57 | 5.95 | 5.19 | 13.51 | 16.04 | 11.37 | 2.89 | 3.19 | 2.60 |
| Guinea | 799.75 | 1121.08 | 563.28 | 8.05 | 10.08 | 6.06 | 12.09 | 16.53 | 8.72 | 1.25 | 1.29 | 1.21 |
| Guinea-Bissau | 143.92 | 196.92 | 101.16 | 9.85 | 14.00 | 6.82 | 15.05 | 20.34 | 10.45 | 1.40 | 1.43 | 1.38 |
| Guyana | 184.86 | 243.70 | 137.56 | 17.22 | 19.62 | 14.98 | 26.20 | 34.22 | 19.59 | 1.64 | 1.90 | 1.38 |
| Haiti | 1803.28 | 2835.35 | 1099.06 | 15.49 | 23.94 | 9.53 | 20.54 | 31.82 | 12.80 | 1.02 | 1.11 | 0.94 |
| Honduras | 1268.98 | 1745.81 | 893.79 | 8.09 | 10.59 | 5.82 | 17.73 | 24.14 | 12.58 | 2.50 | 2.68 | 2.32 |
| Hungary | 6160.56 | 7202.13 | 5209.90 | 32.24 | 36.37 | 28.63 | 36.06 | 42.31 | 30.21 | -0.08 | 0.17 | -0.32 |
| Iceland | 216.03 | 241.96 | 189.42 | 47.41 | 51.83 | 42.78 | 41.87 | 46.80 | 36.72 | -0.47 | -0.34 | -0.61 |
| India | 159271.48 | 187901.49 | 135111.62 | 5.92 | 6.94 | 5.05 | 12.15 | 14.38 | 10.30 | 2.38 | 2.57 | 2.19 |
| Indonesia | 49532.23 | 70939.49 | 33319.17 | 10.14 | 14.25 | 6.99 | 17.39 | 24.75 | 11.69 | 1.62 | 1.71 | 1.53 |
| Iran (Islamic Republic of) | 24764.09 | 27715.31 | 22197.53 | 9.90 | 11.70 | 8.35 | 26.46 | 29.63 | 23.70 | 3.61 | 3.92 | 3.30 |
| Iraq | 9554.25 | 12992.40 | 6666.06 | 13.35 | 17.64 | 9.85 | 31.20 | 42.15 | 21.84 | 2.87 | 2.99 | 2.76 |
| Ireland | 3126.35 | 3456.29 | 2787.20 | 49.13 | 52.72 | 46.02 | 43.34 | 47.78 | 38.81 | -0.03 | 0.16 | -0.22 |
| Israel | 4171.62 | 4644.94 | 3675.55 | 40.98 | 44.06 | 37.98 | 36.71 | 40.81 | 32.58 | -0.56 | -0.28 | -0.83 |
| Italy | 51530.18 | 55879.77 | 44988.07 | 48.63 | 51.26 | 45.84 | 44.23 | 47.57 | 40.25 | -0.40 | -0.20 | -0.59 |
| Jamaica | 1243.64 | 1623.16 | 931.49 | 23.89 | 25.78 | 21.89 | 40.40 | 52.75 | 30.22 | 1.67 | 1.96 | 1.39 |
| Japan | 78401.01 | 85214.55 | 68142.21 | 16.98 | 17.83 | 15.99 | 31.93 | 34.14 | 29.04 | 2.09 | 2.33 | 1.86 |
| Jordan | 3030.90 | 4111.75 | 2143.85 | 17.05 | 22.07 | 13.06 | 32.60 | 43.77 | 23.20 | 2.48 | 2.92 | 2.05 |
| Kazakhstan | 3539.70 | 4149.33 | 2966.52 | 22.73 | 25.44 | 20.06 | 18.15 | 21.28 | 15.20 | -0.01 | 0.22 | -0.23 |
| Kenya | 4823.77 | 6664.82 | 3386.72 | 8.91 | 12.03 | 6.50 | 17.40 | 23.64 | 12.53 | 2.29 | 2.39 | 2.20 |
| Kiribati | 19.22 | 26.01 | 14.39 | 16.28 | 20.81 | 12.57 | 23.53 | 31.49 | 17.85 | 1.20 | 1.28 | 1.13 |
| Kuwait | 1204.42 | 1428.70 | 1019.69 | 15.90 | 17.61 | 14.12 | 26.35 | 31.05 | 22.21 | 1.55 | 2.03 | 1.07 |
| Kyrgyzstan | 811.38 | 980.92 | 660.57 | 17.49 | 19.44 | 15.60 | 14.34 | 17.31 | 11.79 | -0.73 | -0.46 | -0.99 |
| Lao People's Democratic Republic | 823.60 | 1129.83 | 578.64 | 9.10 | 14.51 | 5.48 | 14.62 | 20.06 | 10.42 | 1.65 | 1.69 | 1.62 |
| Latvia | 1074.68 | 1268.09 | 878.61 | 26.97 | 30.69 | 23.94 | 30.54 | 36.43 | 24.87 | 0.32 | 0.51 | 0.13 |
| Lebanon | 3123.36 | 3848.64 | 2480.80 | 24.18 | 32.58 | 17.19 | 52.72 | 65.01 | 41.84 | 2.97 | 3.29 | 2.65 |
| Lesotho | 242.65 | 357.16 | 154.58 | 10.19 | 14.20 | 7.23 | 21.21 | 30.97 | 13.73 | 3.10 | 3.51 | 2.69 |
| Liberia | 342.68 | 496.92 | 233.51 | 7.07 | 8.72 | 5.57 | 12.13 | 17.06 | 8.42 | 1.90 | 2.02 | 1.77 |
| Libya | 1563.44 | 2190.13 | 1120.97 | 9.64 | 12.18 | 7.63 | 23.30 | 31.93 | 16.91 | 3.18 | 3.57 | 2.79 |
| Lithuania | 1486.71 | 1756.46 | 1238.93 | 27.26 | 29.54 | 25.20 | 30.51 | 35.94 | 25.57 | 0.21 | 0.40 | 0.01 |
| Luxembourg | 377.47 | 419.05 | 333.12 | 47.34 | 50.34 | 44.19 | 37.80 | 41.93 | 33.40 | -0.31 | -0.06 | -0.57 |
| Madagascar | 2048.06 | 2805.49 | 1456.76 | 11.07 | 13.65 | 9.03 | 13.90 | 18.41 | 10.10 | 0.64 | 0.85 | 0.44 |
| Malawi | 1421.22 | 1868.10 | 1014.34 | 9.07 | 11.23 | 7.16 | 16.00 | 20.68 | 11.79 | 1.87 | 1.98 | 1.76 |
| Malaysia | 9434.87 | 11197.88 | 7905.31 | 16.52 | 19.21 | 13.97 | 30.28 | 35.84 | 25.48 | 2.01 | 2.11 | 1.91 |
| Maldives | 39.60 | 50.65 | 29.95 | 6.00 | 10.01 | 3.29 | 8.90 | 11.22 | 6.92 | 1.16 | 1.57 | 0.75 |
| Mali | 1310.44 | 1824.54 | 935.44 | 9.44 | 11.42 | 7.69 | 12.33 | 16.89 | 8.97 | 0.79 | 0.87 | 0.71 |
| Malta | 331.16 | 377.51 | 287.95 | 45.48 | 49.78 | 41.89 | 41.48 | 46.95 | 36.62 | -0.48 | -0.32 | -0.65 |
| Marshall Islands | 9.60 | 15.08 | 5.62 | 15.06 | 20.97 | 10.85 | 22.02 | 33.80 | 13.43 | 1.07 | 1.14 | 0.99 |
| Mauritania | 382.98 | 507.06 | 285.34 | 9.86 | 13.36 | 7.15 | 15.69 | 20.69 | 11.79 | 1.29 | 1.45 | 1.14 |
| Mauritius | 503.90 | 542.44 | 455.35 | 10.91 | 11.73 | 10.11 | 28.29 | 30.46 | 25.49 | 2.39 | 2.78 | 2.01 |
| Mexico | 36561.66 | 43119.32 | 30342.92 | 14.80 | 15.21 | 14.36 | 26.95 | 31.72 | 22.43 | 1.54 | 1.70 | 1.37 |
| Micronesia (Federated States of) | 21.40 | 28.91 | 15.16 | 18.26 | 24.98 | 12.51 | 25.89 | 34.60 | 18.54 | 1.05 | 1.12 | 0.99 |
| Monaco | 63.03 | 83.03 | 47.84 | 60.36 | 79.88 | 44.76 | 86.05 | 116.05 | 63.59 | 1.31 | 1.49 | 1.12 |
| Mongolia | 181.86 | 225.18 | 141.82 | 4.18 | 5.34 | 3.22 | 6.30 | 7.79 | 4.84 | 1.26 | 1.37 | 1.14 |
| Montenegro | 418.47 | 533.14 | 322.33 | 35.29 | 46.00 | 27.57 | 45.79 | 58.06 | 35.15 | 1.32 | 1.44 | 1.19 |
| Morocco | 5619.46 | 8227.51 | 3790.70 | 5.68 | 7.38 | 4.38 | 14.81 | 21.45 | 10.10 | 3.32 | 3.40 | 3.23 |
| Mozambique | 2143.32 | 2848.26 | 1488.08 | 9.76 | 11.82 | 8.10 | 16.52 | 21.65 | 11.70 | 1.98 | 2.10 | 1.86 |
| Myanmar | 9610.09 | 12682.92 | 7313.64 | 12.35 | 17.38 | 8.59 | 17.62 | 23.13 | 13.62 | 1.02 | 1.09 | 0.94 |
| Namibia | 477.64 | 684.53 | 308.23 | 13.55 | 16.52 | 11.25 | 29.91 | 41.99 | 19.55 | 2.91 | 3.02 | 2.81 |
| Nauru | 2.23 | 3.47 | 1.29 | 20.73 | 31.80 | 12.46 | 31.60 | 48.60 | 18.87 | 1.28 | 1.36 | 1.20 |
| Nepal | 2141.31 | 2938.51 | 1543.66 | 4.93 | 6.44 | 3.61 | 8.37 | 11.46 | 6.04 | 1.78 | 2.04 | 1.51 |
| Netherlands | 13450.15 | 14773.32 | 12102.77 | 49.37 | 52.36 | 46.19 | 45.62 | 49.79 | 41.59 | -0.16 | 0.06 | -0.37 |
| New Zealand | 3483.02 | 3813.01 | 3086.39 | 57.16 | 61.28 | 52.90 | 46.62 | 50.75 | 41.78 | -0.48 | -0.40 | -0.57 |
| Nicaragua | 955.10 | 1213.09 | 747.26 | 7.84 | 9.22 | 6.65 | 17.26 | 21.78 | 13.54 | 2.87 | 3.07 | 2.67 |
| Niger | 661.26 | 923.27 | 449.41 | 5.29 | 7.09 | 3.96 | 6.88 | 9.51 | 4.75 | 0.85 | 0.92 | 0.78 |
| Nigeria | 26098.66 | 37952.02 | 17281.85 | 11.73 | 15.13 | 8.88 | 23.95 | 34.04 | 16.56 | 2.54 | 2.79 | 2.28 |
| Niue | 0.66 | 0.86 | 0.50 | 23.77 | 31.05 | 17.93 | 32.59 | 42.18 | 24.69 | 0.64 | 0.76 | 0.52 |
| North Macedonia | 1119.09 | 1446.70 | 856.96 | 24.46 | 29.06 | 20.11 | 34.97 | 45.29 | 26.72 | 1.29 | 1.60 | 0.98 |
| Northern Mariana Islands | 15.47 | 18.25 | 12.49 | 22.52 | 29.84 | 16.75 | 27.89 | 32.31 | 22.79 | 0.32 | 0.49 | 0.15 |
| Norway | 2699.52 | 2938.28 | 2448.11 | 34.40 | 36.01 | 32.53 | 30.90 | 33.51 | 28.34 | -0.46 | -0.10 | -0.81 |
| Oman | 243.77 | 319.58 | 181.50 | 4.21 | 5.70 | 3.09 | 9.09 | 11.58 | 6.96 | 2.68 | 3.09 | 2.27 |
| Pakistan | 34748.76 | 46366.89 | 24741.61 | 12.41 | 15.41 | 9.76 | 22.64 | 29.83 | 16.37 | 1.58 | 1.76 | 1.41 |
| Palau | 7.78 | 10.06 | 5.92 | 34.87 | 45.95 | 26.57 | 35.40 | 45.27 | 27.45 | -0.02 | 0.05 | -0.10 |
| Palestine | 1202.27 | 1470.71 | 971.87 | 21.69 | 29.82 | 15.49 | 39.19 | 47.83 | 31.64 | 2.12 | 2.24 | 2.01 |
| Panama | 1627.68 | 1993.96 | 1307.19 | 17.83 | 19.30 | 16.35 | 36.87 | 45.17 | 29.60 | 2.39 | 2.50 | 2.28 |
| Papua New Guinea | 954.88 | 1296.06 | 691.54 | 11.44 | 15.97 | 7.99 | 13.05 | 17.79 | 9.57 | 0.17 | 0.34 | 0.00 |
| Paraguay | 1402.68 | 1854.32 | 1028.31 | 12.10 | 14.60 | 9.73 | 22.47 | 29.73 | 16.58 | 1.96 | 2.14 | 1.78 |
| Peru | 6360.71 | 8382.14 | 4542.11 | 10.87 | 12.97 | 8.85 | 18.04 | 23.87 | 12.95 | 1.30 | 1.53 | 1.08 |
| Philippines | 21561.45 | 26892.22 | 16967.20 | 14.94 | 16.74 | 13.28 | 22.65 | 27.99 | 17.93 | 1.26 | 1.37 | 1.15 |
| Poland | 21058.68 | 23510.41 | 18540.69 | 22.58 | 23.57 | 21.58 | 32.69 | 36.36 | 28.63 | 1.16 | 1.33 | 0.99 |
| Portugal | 7310.21 | 7997.26 | 6495.64 | 35.33 | 37.87 | 33.05 | 38.34 | 42.04 | 34.66 | 0.24 | 0.46 | 0.03 |
| Puerto Rico | 2106.58 | 2512.28 | 1742.15 | 28.54 | 30.67 | 26.54 | 36.95 | 44.04 | 30.47 | 0.85 | 1.01 | 0.69 |
| Qatar | 603.84 | 833.61 | 432.34 | 21.15 | 26.30 | 16.72 | 42.59 | 56.60 | 31.94 | 2.56 | 2.83 | 2.28 |
| Republic of Korea | 16186.35 | 19147.48 | 13078.75 | 6.81 | 8.07 | 5.93 | 19.55 | 23.18 | 15.93 | 3.76 | 4.12 | 3.39 |
| Republic of Moldova | 1586.30 | 1893.06 | 1323.31 | 25.23 | 28.22 | 22.38 | 27.68 | 32.83 | 23.16 | 0.57 | 0.82 | 0.33 |
| Romania | 9728.08 | 11065.17 | 8488.18 | 18.35 | 19.73 | 17.08 | 29.93 | 33.96 | 26.01 | 1.54 | 1.69 | 1.39 |
| Russian Federation | 76078.63 | 83982.06 | 67845.32 | 22.22 | 22.74 | 21.68 | 33.56 | 37.04 | 29.84 | 1.09 | 1.25 | 0.94 |
| Rwanda | 1424.40 | 1966.03 | 989.53 | 15.91 | 22.13 | 11.34 | 19.45 | 26.79 | 13.84 | 0.29 | 0.52 | 0.06 |
| Saint Kitts and Nevis | 26.41 | 32.03 | 21.57 | 39.32 | 42.95 | 35.81 | 36.43 | 43.65 | 29.95 | 0.24 | 0.42 | 0.06 |
| Saint Lucia | 73.61 | 88.75 | 60.86 | 31.41 | 33.94 | 29.20 | 30.89 | 37.31 | 25.57 | -0.22 | -0.01 | -0.44 |
| Saint Vincent and the Grenadines | 48.95 | 56.87 | 42.39 | 31.21 | 34.14 | 27.89 | 35.09 | 40.87 | 30.36 | 0.34 | 0.57 | 0.11 |
| Samoa | 27.99 | 37.30 | 20.24 | 13.55 | 17.43 | 10.32 | 17.95 | 23.83 | 13.02 | 0.73 | 0.83 | 0.63 |
| San Marino | 16.33 | 23.97 | 9.57 | 35.65 | 45.27 | 28.01 | 27.45 | 41.39 | 15.83 | -0.05 | 0.29 | -0.38 |
| Sao Tome and Principe | 20.80 | 27.33 | 15.40 | 7.98 | 9.73 | 6.50 | 15.25 | 19.82 | 11.57 | 2.05 | 2.15 | 1.96 |
| Saudi Arabia | 6190.14 | 8826.21 | 4373.23 | 6.29 | 8.44 | 4.63 | 18.43 | 24.78 | 13.82 | 3.67 | 3.94 | 3.39 |
| Senegal | 1189.64 | 1604.46 | 886.74 | 7.74 | 9.65 | 6.12 | 13.42 | 17.91 | 10.15 | 1.77 | 1.89 | 1.66 |
| Serbia | 5924.72 | 7582.37 | 4494.75 | 29.32 | 37.47 | 22.22 | 40.40 | 51.68 | 30.66 | 0.89 | 1.04 | 0.74 |
| Seychelles | 29.51 | 34.62 | 25.00 | 13.88 | 16.04 | 11.93 | 23.61 | 27.68 | 19.97 | 1.71 | 2.08 | 1.34 |
| Sierra Leone | 504.90 | 667.58 | 353.30 | 6.53 | 8.61 | 4.69 | 11.03 | 14.46 | 7.96 | 1.91 | 1.99 | 1.84 |
| Singapore | 2452.39 | 2666.31 | 2243.25 | 21.42 | 23.06 | 19.96 | 28.35 | 30.91 | 26.07 | 1.28 | 1.52 | 1.03 |
| Slovakia | 2989.99 | 3710.26 | 2282.04 | 25.22 | 29.37 | 21.85 | 33.24 | 41.08 | 25.57 | 0.89 | 1.05 | 0.73 |
| Slovenia | 1233.63 | 1478.68 | 1011.61 | 32.37 | 35.60 | 29.45 | 31.66 | 38.08 | 25.78 | -0.07 | 0.18 | -0.31 |
| Solomon Islands | 69.71 | 96.60 | 48.68 | 8.74 | 12.50 | 5.63 | 15.65 | 21.45 | 10.98 | 1.83 | 1.99 | 1.67 |
| Somalia | 803.56 | 1122.69 | 527.39 | 9.20 | 12.45 | 6.41 | 10.50 | 14.50 | 7.16 | 0.43 | 0.47 | 0.39 |
| South Africa | 12094.18 | 13506.19 | 10835.27 | 14.87 | 17.86 | 11.95 | 24.29 | 26.95 | 21.91 | 2.06 | 2.26 | 1.86 |
| South Sudan | 578.79 | 837.48 | 406.09 | 8.63 | 12.15 | 6.16 | 12.06 | 16.92 | 8.64 | 1.02 | 1.25 | 0.79 |
| Spain | 26779.74 | 29682.14 | 23479.74 | 36.03 | 38.35 | 33.59 | 32.79 | 36.12 | 29.17 | -0.36 | -0.18 | -0.53 |
| Sri Lanka | 4474.85 | 6152.30 | 2873.98 | 8.77 | 10.54 | 7.28 | 16.64 | 22.81 | 10.74 | 2.44 | 2.62 | 2.27 |
| Sudan | 2853.40 | 4511.12 | 1677.87 | 4.75 | 7.13 | 3.02 | 10.65 | 16.52 | 6.56 | 2.70 | 2.88 | 2.52 |
| Suriname | 130.48 | 166.82 | 99.86 | 15.51 | 18.16 | 13.03 | 20.17 | 25.81 | 15.39 | 1.08 | 1.23 | 0.94 |
| Sweden | 5996.81 | 6924.02 | 4991.58 | 40.45 | 43.29 | 37.51 | 32.88 | 37.87 | 27.60 | -0.32 | 0.06 | -0.69 |
| Switzerland | 5294.88 | 5853.15 | 4682.67 | 40.05 | 42.67 | 37.42 | 32.97 | 36.23 | 29.76 | -0.63 | -0.22 | -1.05 |
| Syrian Arab Republic | 3371.25 | 4534.64 | 2459.83 | 9.71 | 12.16 | 7.34 | 22.84 | 30.62 | 16.73 | 2.68 | 2.79 | 2.58 |
| Taiwan (Province of China) | 10867.26 | 11888.89 | 9842.45 | 11.65 | 12.43 | 10.90 | 28.61 | 31.30 | 25.80 | 2.98 | 3.38 | 2.59 |
| Tajikistan | 766.22 | 1139.20 | 467.53 | 12.40 | 14.95 | 10.03 | 10.16 | 14.92 | 6.44 | -0.82 | -0.66 | -0.97 |
| Thailand | 24635.62 | 31808.28 | 18506.30 | 10.46 | 12.44 | 8.51 | 24.43 | 31.69 | 18.32 | 2.85 | 3.12 | 2.59 |
| Timor-Leste | 99.38 | 135.36 | 67.42 | 6.15 | 9.18 | 3.98 | 10.83 | 14.79 | 7.39 | 2.03 | 2.25 | 1.81 |
| Togo | 694.72 | 951.08 | 487.84 | 9.28 | 11.53 | 7.53 | 14.77 | 19.94 | 10.72 | 1.49 | 1.60 | 1.37 |
| Tokelau | 0.42 | 0.56 | 0.31 | 21.50 | 30.45 | 14.12 | 29.49 | 39.32 | 21.91 | 0.79 | 0.89 | 0.70 |
| Tonga | 28.19 | 37.99 | 20.57 | 28.71 | 36.26 | 22.71 | 33.78 | 45.43 | 24.75 | 0.23 | 0.34 | 0.12 |
| Trinidad and Tobago | 663.71 | 852.28 | 498.20 | 27.60 | 30.07 | 25.50 | 35.45 | 45.61 | 26.61 | 0.96 | 1.15 | 0.77 |
| Tunisia | 3487.14 | 4860.32 | 2483.71 | 11.63 | 14.31 | 9.51 | 25.10 | 34.94 | 17.87 | 2.41 | 2.54 | 2.28 |
| Turkmenistan | 694.94 | 939.53 | 509.24 | 12.87 | 14.60 | 11.22 | 14.54 | 19.50 | 10.77 | 6.77 | 7.63 | 5.92 |
| Tuvalu | 2.43 | 3.36 | 1.72 | 17.62 | 25.31 | 11.71 | 22.70 | 31.27 | 16.27 | 0.91 | 1.39 | 0.43 |
| Turkey | 28899.68 | 35853.31 | 22612.04 | 6.10 | 7.44 | 4.90 | 29.92 | 36.97 | 23.33 | 0.63 | 0.74 | 0.52 |
| Uganda | 4063.85 | 5449.58 | 2963.80 | 12.61 | 16.67 | 9.29 | 22.70 | 29.97 | 16.94 | 1.38 | 1.62 | 1.14 |
| Ukraine | 15687.53 | 22758.72 | 10102.19 | 28.15 | 30.32 | 25.99 | 21.89 | 32.23 | 13.88 | -1.27 | -1.08 | -1.45 |
| United Arab Emirates | 1475.84 | 1978.01 | 1059.25 | 17.05 | 22.15 | 12.87 | 29.08 | 38.63 | 21.35 | 3.32 | 3.87 | 2.77 |
| United Kingdom | 49440.22 | 51651.32 | 45755.97 | 56.19 | 57.43 | 54.27 | 43.86 | 45.51 | 41.31 | -0.77 | -0.64 | -0.89 |
| United Republic of Tanzania | 5378.90 | 7111.72 | 3935.63 | 12.20 | 14.77 | 9.89 | 17.62 | 23.24 | 13.23 | 1.12 | 1.20 | 1.03 |
| United States of America | 272387.51 | 285257.70 | 251345.10 | 68.32 | 70.31 | 65.13 | 51.76 | 54.11 | 48.36 | -1.08 | -0.99 | -1.18 |
| United States Virgin Islands | 48.53 | 68.37 | 33.43 | 36.78 | 44.26 | 30.53 | 33.43 | 47.61 | 23.23 | -0.06 | 0.09 | -0.21 |
| Uruguay | 2036.06 | 2213.63 | 1829.83 | 36.99 | 39.16 | 34.74 | 41.12 | 44.63 | 36.94 | 0.19 | 0.32 | 0.07 |
| Uzbekistan | 4243.62 | 5085.52 | 3460.22 | 13.26 | 14.77 | 11.89 | 13.18 | 15.82 | 10.82 | 0.16 | 0.39 | -0.08 |
| Vanuatu | 31.01 | 40.65 | 22.48 | 9.51 | 13.39 | 6.61 | 15.07 | 19.24 | 11.19 | 1.19 | 1.32 | 1.06 |
| Venezuela (Bolivarian Republic of) | 12010.33 | 15577.20 | 9029.75 | 18.80 | 19.98 | 17.54 | 38.57 | 49.84 | 29.08 | 2.20 | 2.36 | 2.04 |
| Viet Nam | 15969.79 | 21410.11 | 12014.68 | 7.63 | 9.94 | 5.90 | 14.55 | 19.28 | 11.00 | 2.17 | 2.21 | 2.13 |
| Yemen | 1722.16 | 2437.81 | 1169.69 | 4.09 | 5.70 | 2.68 | 9.20 | 12.82 | 6.40 | 2.92 | 3.07 | 2.77 |
| Zambia | 2390.53 | 3771.43 | 1331.83 | 11.47 | 15.44 | 8.46 | 25.10 | 37.77 | 15.34 | 2.73 | 3.11 | 2.35 |
| Zimbabwe | 2004.08 | 2761.80 | 1439.47 | 12.00 | 15.27 | 9.30 | 23.98 | 32.24 | 17.47 | 2.94 | 3.64 | 2.24 |

EAPC: estimated annual percentage change; UI: uncertainty interval; CI: confidence interval.

Table S3. The all-ages numbers and the age-standardized rates of mortality, and its corresponding EAPC of breast cancer among 204 countries in 1990 and 2021

| Characteristics | Counts (2021) | | | Age-standardized mortality rate (per 100,000) (95% UI) | | | | | | EAPC (95% CI)1990–2021 | | |
| --- | --- | --- | --- | --- | --- | --- | --- | --- | --- | --- | --- | --- |
|  |  |  |  | 1990 | | | 2021 | | |  |  |  |
|  | val | upper | lower | val | upper | lower | val | upper | lower | val | upper | lower |
| Afghanistan | 335.89 | 561.91 | 177.07 | 4.71 | 7.69 | 2.60 | 7.27 | 11.86 | 4.19 | 1.54 | 1.63 | 1.45 |
| Albania | 105.38 | 132.33 | 82.65 | 5.04 | 6.35 | 3.93 | 5.69 | 7.62 | 3.98 | 0.77 | 0.95 | 0.59 |
| Algeria | 452.29 | 582.58 | 346.71 | 3.81 | 4.85 | 2.94 | 4.22 | 5.35 | 3.24 | 0.61 | 0.74 | 0.48 |
| American Samoa | 3.19 | 3.83 | 2.63 | 13.97 | 16.75 | 11.67 | 20.79 | 25.70 | 16.46 | 1.60 | 1.71 | 1.49 |
| Andorra | 6.67 | 9.73 | 4.66 | 11.98 | 17.34 | 8.42 | 8.85 | 12.08 | 6.18 | -0.57 | -0.40 | -0.74 |
| Angola | 301.05 | 417.15 | 215.21 | 6.85 | 9.02 | 5.11 | 10.52 | 13.91 | 7.42 | 1.45 | 1.53 | 1.38 |
| Antigua and Barbuda | 7.76 | 8.66 | 7.00 | 14.66 | 16.34 | 13.18 | 16.83 | 18.18 | 15.65 | 0.64 | 0.84 | 0.44 |
| Argentina | 5436.70 | 5719.67 | 5085.73 | 17.22 | 18.18 | 16.11 | 13.25 | 14.45 | 11.92 | -0.78 | -0.62 | -0.93 |
| Armenia | 454.09 | 485.93 | 420.84 | 15.99 | 17.19 | 14.78 | 11.65 | 13.50 | 10.20 | -1.06 | -0.75 | -1.36 |
| Australia | 2613.48 | 2779.45 | 2436.21 | 13.83 | 14.67 | 12.87 | 8.12 | 9.11 | 7.08 | -1.83 | -1.75 | -1.90 |
| Austria | 1871.22 | 2002.62 | 1726.88 | 16.40 | 17.46 | 15.23 | 9.20 | 10.16 | 8.05 | -1.80 | -1.74 | -1.86 |
| Azerbaijan | 507.79 | 577.45 | 424.64 | 9.85 | 11.13 | 8.26 | 7.78 | 9.81 | 5.97 | -0.62 | -0.48 | -0.76 |
| Bahamas | 32.19 | 35.20 | 29.55 | 19.47 | 21.24 | 17.88 | 20.66 | 25.15 | 16.93 | 0.41 | 0.54 | 0.27 |
| Bahrain | 22.29 | 26.01 | 18.70 | 11.48 | 13.67 | 9.64 | 10.64 | 13.71 | 8.35 | -0.53 | -0.31 | -0.75 |
| Bangladesh | 1638.56 | 2218.07 | 1232.29 | 2.76 | 3.71 | 2.10 | 3.75 | 4.81 | 2.85 | 0.79 | 0.93 | 0.64 |
| Barbados | 49.10 | 53.11 | 44.81 | 18.06 | 19.47 | 16.56 | 19.41 | 23.90 | 15.77 | 0.67 | 0.89 | 0.45 |
| Belarus | 1247.79 | 1361.16 | 1135.69 | 9.87 | 10.77 | 8.99 | 8.73 | 10.92 | 6.95 | -1.31 | -1.02 | -1.59 |
| Belgium | 2936.67 | 3125.41 | 2667.69 | 20.00 | 21.20 | 18.27 | 10.83 | 11.97 | 9.41 | -1.96 | -1.86 | -2.05 |
| Belize | 4.74 | 5.17 | 4.30 | 4.97 | 5.42 | 4.51 | 6.86 | 7.70 | 6.06 | 1.04 | 1.36 | 0.72 |
| Benin | 125.16 | 150.66 | 102.24 | 6.05 | 7.24 | 4.98 | 7.37 | 9.64 | 5.58 | 0.61 | 0.67 | 0.54 |
| Bermuda | 12.67 | 13.91 | 11.44 | 20.86 | 22.82 | 18.78 | 11.16 | 13.99 | 9.16 | -2.38 | -2.17 | -2.58 |
| Bhutan | 10.46 | 13.68 | 7.32 | 3.75 | 4.93 | 2.68 | 4.30 | 5.75 | 3.12 | 0.38 | 0.50 | 0.27 |
| Bolivia (Plurinational State of) | 283.57 | 409.35 | 185.77 | 8.37 | 11.99 | 5.58 | 9.53 | 13.75 | 6.35 | 0.30 | 0.35 | 0.26 |
| Bosnia and Herzegovina | 319.47 | 360.11 | 272.91 | 7.59 | 8.48 | 6.57 | 9.49 | 11.72 | 7.49 | 1.14 | 1.28 | 0.99 |
| Botswana | 53.56 | 74.26 | 37.39 | 9.70 | 13.13 | 6.90 | 12.48 | 16.79 | 9.53 | 1.28 | 1.60 | 0.96 |
| Brazil | 8381.64 | 8696.47 | 8032.25 | 9.05 | 9.42 | 8.55 | 9.43 | 10.01 | 8.70 | -0.05 | 0.01 | -0.12 |
| Brunei Darussalam | 10.75 | 14.18 | 8.00 | 8.18 | 10.69 | 6.18 | 9.33 | 11.42 | 7.32 | 1.04 | 1.27 | 0.82 |
| Bulgaria | 1312.58 | 1496.41 | 1152.14 | 11.42 | 12.97 | 10.05 | 13.19 | 15.65 | 10.80 | 0.68 | 0.82 | 0.55 |
| Burkina Faso | 459.36 | 581.82 | 353.16 | 10.65 | 13.33 | 8.45 | 12.08 | 15.59 | 9.10 | 0.41 | 0.52 | 0.31 |
| Burundi | 237.28 | 333.84 | 169.34 | 9.85 | 13.72 | 7.18 | 8.39 | 11.51 | 6.33 | -0.87 | -0.70 | -1.04 |
| Cabo Verde | 17.76 | 21.54 | 14.46 | 8.26 | 10.10 | 6.69 | 8.43 | 10.69 | 6.46 | 0.31 | 0.59 | 0.04 |
| Cambodia | 381.62 | 578.82 | 243.55 | 7.66 | 11.42 | 5.06 | 11.53 | 15.07 | 8.37 | 1.31 | 1.36 | 1.26 |
| Cameroon | 371.36 | 461.04 | 297.56 | 7.87 | 9.68 | 6.34 | 9.76 | 13.27 | 7.14 | 0.64 | 0.70 | 0.57 |
| Canada | 4822.11 | 5138.54 | 4433.23 | 15.10 | 16.07 | 13.87 | 8.57 | 9.45 | 7.55 | -1.94 | -1.87 | -2.01 |
| Central African Republic | 108.96 | 148.03 | 78.27 | 8.88 | 11.35 | 6.67 | 10.34 | 13.53 | 7.51 | 0.44 | 0.49 | 0.40 |
| Chad | 141.89 | 186.15 | 103.01 | 4.95 | 6.53 | 3.64 | 6.13 | 8.04 | 4.54 | 0.74 | 0.78 | 0.71 |
| Chile | 974.11 | 1043.73 | 908.17 | 9.76 | 10.49 | 9.04 | 7.00 | 7.69 | 6.23 | -0.88 | -0.79 | -0.96 |
| China | 41217.71 | 50194.44 | 33621.06 | 4.70 | 5.66 | 3.87 | 4.40 | 5.46 | 3.45 | -0.50 | -0.39 | -0.62 |
| Colombia | 1423.56 | 1513.62 | 1333.02 | 7.60 | 8.08 | 7.12 | 7.66 | 9.10 | 6.41 | -0.02 | 0.20 | -0.24 |
| Comoros | 19.62 | 26.53 | 14.24 | 9.49 | 12.62 | 6.97 | 13.22 | 17.71 | 9.88 | 0.87 | 0.97 | 0.77 |
| Congo | 138.28 | 220.21 | 81.67 | 12.54 | 19.11 | 7.83 | 15.22 | 22.72 | 9.91 | 0.53 | 0.64 | 0.43 |
| Cook Islands | 2.39 | 3.02 | 1.86 | 19.58 | 24.30 | 15.40 | 20.62 | 26.26 | 15.68 | 0.29 | 0.43 | 0.14 |
| Costa Rica | 118.44 | 126.81 | 109.29 | 6.50 | 6.96 | 6.00 | 8.93 | 10.15 | 7.77 | 1.12 | 1.27 | 0.97 |
| Croatia | 831.25 | 938.43 | 734.69 | 14.37 | 16.20 | 12.72 | 10.80 | 12.38 | 9.22 | -0.81 | -0.63 | -1.00 |
| Cuba | 998.06 | 1057.07 | 938.61 | 9.87 | 10.45 | 9.27 | 9.48 | 11.07 | 8.12 | -0.06 | 0.06 | -0.17 |
| Cyprus | 101.19 | 122.34 | 82.50 | 13.82 | 16.86 | 11.09 | 11.39 | 13.64 | 9.39 | -0.43 | -0.30 | -0.56 |
| Czechia | 1948.48 | 2174.88 | 1757.35 | 14.46 | 16.14 | 13.05 | 9.27 | 10.91 | 7.76 | -1.80 | -1.60 | -1.99 |
| Côte d'Ivoire | 367.31 | 471.07 | 284.47 | 8.48 | 10.59 | 6.79 | 11.22 | 14.77 | 8.44 | 1.00 | 1.11 | 0.89 |
| Democratic People's Republic of Korea | 992.16 | 1437.48 | 655.96 | 5.72 | 8.20 | 3.82 | 6.18 | 8.21 | 4.39 | 0.42 | 0.51 | 0.34 |
| Democratic Republic of the Congo | 1251.78 | 1714.66 | 876.93 | 7.38 | 9.84 | 5.40 | 10.12 | 13.79 | 7.49 | 1.07 | 1.26 | 0.88 |
| Denmark | 1734.62 | 1834.14 | 1617.14 | 22.45 | 23.68 | 21.04 | 10.89 | 11.96 | 9.51 | -2.38 | -2.28 | -2.48 |
| Djibouti | 14.94 | 20.04 | 10.88 | 9.91 | 12.87 | 7.51 | 12.20 | 16.82 | 8.71 | 0.62 | 0.67 | 0.58 |
| Dominica | 9.54 | 11.07 | 7.98 | 16.63 | 19.21 | 14.06 | 17.04 | 21.21 | 13.09 | 0.18 | 0.25 | 0.11 |
| Dominican Republic | 239.63 | 281.40 | 201.76 | 6.32 | 7.41 | 5.36 | 7.41 | 9.58 | 5.66 | 0.88 | 1.09 | 0.67 |
| Ecuador | 267.36 | 283.67 | 251.41 | 4.76 | 5.04 | 4.48 | 6.77 | 8.52 | 5.29 | 1.27 | 1.47 | 1.07 |
| Egypt | 1402.99 | 1672.87 | 1220.52 | 4.36 | 5.11 | 3.86 | 8.77 | 10.67 | 7.14 | 2.87 | 3.30 | 2.44 |
| El Salvador | 132.87 | 151.11 | 116.01 | 4.26 | 4.83 | 3.72 | 6.84 | 8.49 | 5.41 | 1.57 | 1.68 | 1.45 |
| Equatorial Guinea | 17.01 | 24.43 | 11.41 | 8.26 | 11.31 | 5.81 | 14.23 | 21.51 | 8.91 | 1.88 | 1.98 | 1.78 |
| Eritrea | 137.95 | 188.46 | 100.16 | 10.86 | 14.22 | 8.22 | 14.95 | 19.98 | 10.67 | 1.04 | 1.14 | 0.94 |
| Estonia | 253.12 | 272.95 | 233.40 | 12.65 | 13.67 | 11.63 | 9.09 | 10.75 | 7.39 | -1.29 | -1.14 | -1.44 |
| Eswatini | 28.74 | 36.80 | 21.88 | 10.00 | 12.75 | 7.63 | 16.08 | 24.21 | 9.90 | 1.79 | 2.17 | 1.41 |
| Ethiopia | 2075.58 | 3049.98 | 1382.73 | 10.26 | 14.49 | 7.19 | 10.55 | 12.80 | 8.74 | -0.06 | 0.11 | -0.24 |
| Fiji | 66.68 | 84.36 | 52.68 | 16.37 | 20.74 | 13.04 | 21.06 | 26.73 | 15.82 | 0.86 | 0.95 | 0.76 |
| Finland | 907.59 | 965.37 | 852.68 | 13.17 | 13.99 | 12.37 | 8.27 | 9.09 | 7.25 | -1.48 | -1.42 | -1.53 |
| France | 12971.14 | 13779.97 | 12006.51 | 16.20 | 17.13 | 15.07 | 10.62 | 11.65 | 9.32 | -1.38 | -1.26 | -1.49 |
| Gabon | 66.07 | 88.54 | 47.52 | 11.76 | 15.41 | 8.57 | 14.71 | 19.73 | 10.31 | 0.55 | 0.66 | 0.44 |
| Gambia | 11.73 | 15.52 | 8.73 | 3.20 | 4.14 | 2.47 | 5.30 | 6.89 | 3.85 | 1.47 | 1.65 | 1.30 |
| Georgia | 959.70 | 1058.88 | 867.17 | 15.61 | 17.16 | 14.12 | 16.40 | 18.94 | 14.12 | 0.56 | 0.86 | 0.27 |
| Germany | 20718.64 | 22034.89 | 19236.21 | 16.84 | 17.87 | 15.66 | 10.76 | 11.68 | 9.51 | -1.53 | -1.43 | -1.63 |
| Ghana | 626.28 | 794.92 | 483.93 | 9.03 | 11.47 | 7.02 | 11.26 | 14.64 | 8.53 | 0.60 | 0.66 | 0.54 |
| Greece | 1998.30 | 2111.92 | 1863.43 | 13.86 | 14.64 | 12.91 | 11.75 | 12.72 | 10.50 | -0.89 | -0.71 | -1.07 |
| Greenland | 5.84 | 7.26 | 4.67 | 16.64 | 20.42 | 13.63 | 8.43 | 10.82 | 6.28 | -2.48 | -2.32 | -2.64 |
| Grenada | 10.37 | 11.43 | 9.36 | 14.98 | 16.58 | 13.55 | 17.33 | 19.45 | 15.12 | 0.86 | 1.15 | 0.58 |
| Guam | 6.60 | 7.70 | 5.64 | 9.65 | 11.17 | 8.27 | 7.01 | 8.56 | 5.80 | -0.26 | 0.04 | -0.57 |
| Guatemala | 114.35 | 120.91 | 106.98 | 3.19 | 3.35 | 3.00 | 4.80 | 5.57 | 4.05 | 1.33 | 1.58 | 1.09 |
| Guinea | 219.18 | 275.45 | 164.29 | 6.40 | 8.04 | 4.80 | 8.58 | 11.62 | 6.23 | 0.93 | 0.97 | 0.88 |
| Guinea-Bissau | 35.07 | 50.94 | 24.32 | 8.04 | 11.34 | 5.69 | 11.13 | 14.89 | 7.74 | 1.10 | 1.12 | 1.08 |
| Guyana | 44.17 | 50.35 | 38.66 | 10.92 | 12.35 | 9.62 | 13.59 | 17.40 | 10.40 | 1.07 | 1.31 | 0.83 |
| Haiti | 394.11 | 610.95 | 239.49 | 11.56 | 17.44 | 7.32 | 13.41 | 20.18 | 8.49 | 0.58 | 0.65 | 0.51 |
| Honduras | 91.15 | 119.15 | 64.92 | 4.12 | 5.55 | 2.83 | 7.14 | 9.73 | 5.17 | 1.77 | 1.93 | 1.61 |
| Hungary | 2152.56 | 2414.99 | 1916.35 | 15.42 | 17.35 | 13.68 | 11.62 | 13.53 | 9.90 | -1.22 | -1.07 | -1.37 |
| Iceland | 40.16 | 43.55 | 36.69 | 14.40 | 15.57 | 13.17 | 8.95 | 9.99 | 7.76 | -1.51 | -1.40 | -1.63 |
| India | 22510.66 | 26349.53 | 19225.89 | 4.24 | 5.01 | 3.57 | 6.52 | 7.72 | 5.52 | 1.44 | 1.59 | 1.29 |
| Indonesia | 8000.83 | 11346.58 | 5502.58 | 7.16 | 10.05 | 4.93 | 9.94 | 14.24 | 6.55 | 0.96 | 1.08 | 0.85 |
| Iran (Islamic Republic of) | 926.61 | 1066.67 | 796.74 | 3.23 | 3.68 | 2.78 | 4.75 | 5.26 | 4.28 | 1.73 | 2.02 | 1.45 |
| Iraq | 536.60 | 704.45 | 405.79 | 6.08 | 7.98 | 4.58 | 8.43 | 11.14 | 6.03 | 0.98 | 1.05 | 0.91 |
| Ireland | 704.60 | 749.86 | 661.21 | 18.34 | 19.45 | 17.21 | 9.28 | 10.33 | 8.14 | -1.99 | -1.89 | -2.09 |
| Israel | 768.67 | 822.68 | 712.89 | 16.72 | 17.84 | 15.51 | 10.17 | 11.23 | 8.73 | -2.02 | -1.79 | -2.24 |
| Italy | 13040.00 | 13601.29 | 12147.25 | 15.56 | 16.21 | 14.54 | 9.89 | 10.76 | 8.59 | -1.54 | -1.48 | -1.60 |
| Jamaica | 189.66 | 204.83 | 175.11 | 10.90 | 11.80 | 10.09 | 14.73 | 18.60 | 11.19 | 0.94 | 1.23 | 0.65 |
| Japan | 6605.09 | 6812.72 | 6280.99 | 3.95 | 4.08 | 3.75 | 5.42 | 5.76 | 4.79 | 1.02 | 1.16 | 0.87 |
| Jordan | 110.92 | 142.68 | 84.64 | 7.09 | 8.94 | 5.53 | 7.53 | 10.05 | 5.40 | 0.30 | 0.65 | -0.06 |
| Kazakhstan | 1638.36 | 1828.52 | 1445.40 | 12.60 | 14.05 | 11.11 | 7.56 | 8.79 | 6.28 | -1.04 | -0.67 | -1.42 |
| Kenya | 576.64 | 781.34 | 417.15 | 6.57 | 8.86 | 4.80 | 11.48 | 15.62 | 8.33 | 2.06 | 2.16 | 1.96 |
| Kiribati | 5.05 | 6.43 | 3.90 | 13.08 | 16.77 | 10.03 | 18.59 | 24.68 | 14.09 | 1.24 | 1.32 | 1.16 |
| Kuwait | 34.16 | 37.46 | 30.72 | 4.79 | 5.24 | 4.23 | 4.39 | 5.08 | 3.69 | -0.27 | 0.13 | -0.66 |
| Kyrgyzstan | 305.98 | 339.13 | 276.25 | 10.18 | 11.27 | 9.17 | 6.42 | 7.67 | 5.29 | -1.56 | -1.37 | -1.75 |
| Lao People's Democratic Republic | 164.15 | 264.08 | 96.82 | 7.39 | 11.54 | 4.56 | 9.43 | 12.92 | 6.82 | 0.87 | 0.92 | 0.82 |
| Latvia | 417.76 | 469.06 | 374.32 | 11.96 | 13.42 | 10.73 | 10.84 | 12.75 | 8.94 | -0.34 | -0.15 | -0.53 |
| Lebanon | 221.90 | 296.01 | 160.35 | 10.30 | 13.62 | 7.59 | 11.11 | 13.76 | 8.88 | 0.46 | 0.67 | 0.26 |
| Lesotho | 67.31 | 93.30 | 47.68 | 8.16 | 11.33 | 5.78 | 16.13 | 23.05 | 10.43 | 2.97 | 3.39 | 2.55 |
| Liberia | 66.47 | 82.15 | 52.39 | 5.71 | 7.03 | 4.53 | 7.99 | 11.13 | 5.64 | 1.21 | 1.34 | 1.09 |
| Libya | 83.51 | 106.64 | 66.12 | 4.10 | 5.19 | 3.27 | 6.53 | 8.71 | 4.92 | 1.66 | 1.87 | 1.44 |
| Lithuania | 471.20 | 509.85 | 432.18 | 10.67 | 11.49 | 9.81 | 10.18 | 11.93 | 8.49 | -0.16 | 0.05 | -0.37 |
| Luxembourg | 99.68 | 106.23 | 93.15 | 18.94 | 20.17 | 17.75 | 9.51 | 10.60 | 8.40 | -1.86 | -1.70 | -2.02 |
| Madagascar | 473.03 | 585.23 | 380.83 | 8.72 | 10.61 | 7.21 | 9.85 | 12.80 | 7.21 | 0.34 | 0.53 | 0.15 |
| Malawi | 299.39 | 368.47 | 237.29 | 7.32 | 8.96 | 5.84 | 11.51 | 14.93 | 8.57 | 1.50 | 1.60 | 1.41 |
| Malaysia | 1074.30 | 1253.43 | 909.74 | 10.27 | 11.87 | 8.76 | 13.23 | 15.39 | 11.27 | 0.80 | 0.89 | 0.71 |
| Maldives | 4.23 | 7.18 | 2.23 | 4.07 | 6.60 | 2.37 | 3.83 | 4.79 | 3.03 | -0.54 | -0.25 | -0.84 |
| Mali | 318.60 | 384.28 | 258.63 | 7.41 | 8.89 | 5.99 | 8.44 | 11.42 | 6.18 | 0.37 | 0.45 | 0.28 |
| Malta | 80.95 | 87.63 | 74.72 | 19.30 | 20.95 | 17.74 | 10.72 | 12.13 | 9.30 | -2.21 | -2.06 | -2.36 |
| Marshall Islands | 1.92 | 2.70 | 1.36 | 11.31 | 15.39 | 8.28 | 15.39 | 23.03 | 9.63 | 0.91 | 1.02 | 0.81 |
| Mauritania | 78.67 | 106.29 | 56.75 | 7.85 | 10.52 | 5.66 | 9.31 | 12.28 | 7.17 | 0.38 | 0.50 | 0.25 |
| Mauritius | 48.61 | 52.27 | 45.35 | 6.49 | 6.97 | 6.03 | 12.47 | 13.26 | 11.32 | 1.59 | 1.90 | 1.28 |
| Mexico | 2830.54 | 2909.15 | 2746.51 | 6.20 | 6.38 | 5.99 | 7.29 | 8.49 | 6.15 | 0.29 | 0.39 | 0.20 |
| Micronesia (Federated States of) | 6.73 | 9.36 | 4.61 | 13.65 | 18.69 | 9.47 | 17.80 | 23.34 | 13.15 | 0.85 | 0.91 | 0.79 |
| Monaco | 12.23 | 16.18 | 8.76 | 18.66 | 24.61 | 13.58 | 18.88 | 24.95 | 14.03 | 0.11 | 0.20 | 0.02 |
| Mongolia | 32.65 | 41.33 | 25.39 | 2.92 | 3.74 | 2.22 | 3.28 | 4.06 | 2.51 | 0.11 | 0.28 | -0.06 |
| Montenegro | 79.05 | 102.58 | 60.76 | 12.62 | 16.35 | 9.71 | 14.80 | 18.73 | 11.55 | 0.63 | 0.77 | 0.50 |
| Morocco | 453.51 | 582.75 | 353.49 | 2.96 | 3.77 | 2.36 | 4.86 | 6.85 | 3.35 | 1.84 | 1.94 | 1.74 |
| Mozambique | 491.61 | 598.04 | 405.95 | 8.35 | 10.10 | 6.92 | 12.94 | 17.15 | 9.17 | 1.76 | 1.90 | 1.63 |
| Myanmar | 2400.56 | 3405.26 | 1653.28 | 9.08 | 12.54 | 6.44 | 10.21 | 13.36 | 7.94 | 0.23 | 0.33 | 0.14 |
| Namibia | 67.65 | 82.51 | 55.93 | 10.27 | 12.47 | 8.48 | 18.31 | 24.60 | 12.34 | 2.19 | 2.32 | 2.06 |
| Nauru | 0.75 | 1.17 | 0.45 | 13.93 | 21.31 | 8.39 | 21.00 | 32.24 | 12.71 | 1.38 | 1.46 | 1.31 |
| Nepal | 399.18 | 519.53 | 295.11 | 3.78 | 4.90 | 2.80 | 4.83 | 6.69 | 3.47 | 0.86 | 1.12 | 0.59 |
| Netherlands | 3728.61 | 3990.58 | 3360.76 | 19.14 | 20.43 | 17.37 | 10.96 | 12.01 | 9.76 | -2.04 | -1.91 | -2.17 |
| New Zealand | 673.29 | 718.75 | 624.82 | 17.85 | 18.99 | 16.58 | 9.48 | 10.39 | 8.35 | -1.96 | -1.89 | -2.04 |
| Nicaragua | 56.68 | 66.14 | 48.27 | 3.31 | 3.89 | 2.80 | 4.82 | 6.03 | 3.77 | 1.49 | 1.69 | 1.28 |
| Niger | 131.21 | 176.06 | 98.51 | 4.35 | 5.89 | 3.25 | 5.08 | 6.91 | 3.52 | 0.47 | 0.56 | 0.39 |
| Nigeria | 4197.26 | 5415.94 | 3176.42 | 9.50 | 12.15 | 7.27 | 15.92 | 21.98 | 11.41 | 1.84 | 2.05 | 1.62 |
| Niue | 0.35 | 0.45 | 0.27 | 15.58 | 20.25 | 11.92 | 18.79 | 24.16 | 14.33 | 0.42 | 0.49 | 0.35 |
| North Macedonia | 234.71 | 282.47 | 192.62 | 12.16 | 14.68 | 9.95 | 13.93 | 17.48 | 10.86 | 0.43 | 0.70 | 0.17 |
| Northern Mariana Islands | 2.47 | 3.30 | 1.76 | 12.23 | 15.84 | 9.27 | 14.58 | 16.73 | 12.18 | 0.55 | 0.72 | 0.38 |
| Norway | 855.45 | 898.40 | 789.26 | 13.24 | 13.85 | 12.34 | 7.22 | 7.86 | 6.42 | -2.18 | -2.04 | -2.32 |
| Oman | 14.23 | 19.26 | 10.27 | 1.94 | 2.61 | 1.45 | 2.16 | 2.68 | 1.71 | 0.60 | 0.92 | 0.28 |
| Pakistan | 5634.07 | 7033.68 | 4440.10 | 9.32 | 11.74 | 7.31 | 14.67 | 19.11 | 10.72 | 1.11 | 1.33 | 0.90 |
| Palau | 2.15 | 2.80 | 1.66 | 22.44 | 28.84 | 17.38 | 21.90 | 27.70 | 17.28 | 0.05 | 0.15 | -0.05 |
| Palestine | 87.78 | 121.51 | 63.73 | 9.79 | 13.43 | 7.22 | 11.19 | 13.56 | 8.99 | 0.67 | 0.81 | 0.53 |
| Panama | 91.07 | 97.39 | 84.30 | 5.91 | 6.31 | 5.46 | 7.46 | 8.96 | 5.92 | 0.92 | 1.03 | 0.80 |
| Papua New Guinea | 170.84 | 242.60 | 117.26 | 7.54 | 10.56 | 5.32 | 8.66 | 11.81 | 6.36 | 0.34 | 0.44 | 0.25 |
| Paraguay | 152.89 | 185.16 | 123.95 | 6.59 | 7.96 | 5.32 | 9.51 | 12.63 | 7.04 | 1.25 | 1.43 | 1.07 |
| Peru | 830.15 | 985.90 | 686.07 | 6.48 | 7.73 | 5.34 | 6.14 | 8.26 | 4.44 | -0.61 | -0.40 | -0.83 |
| Philippines | 3215.85 | 3555.35 | 2864.73 | 10.12 | 11.29 | 9.00 | 12.86 | 15.85 | 10.24 | 0.77 | 0.86 | 0.68 |
| Poland | 5060.08 | 5228.06 | 4855.99 | 11.97 | 12.37 | 11.48 | 12.09 | 13.44 | 10.68 | -0.23 | -0.10 | -0.36 |
| Portugal | 1827.71 | 1947.09 | 1706.46 | 14.39 | 15.32 | 13.49 | 8.75 | 9.49 | 7.80 | -1.73 | -1.65 | -1.80 |
| Puerto Rico | 365.50 | 388.69 | 339.11 | 10.44 | 11.11 | 9.66 | 8.82 | 10.38 | 7.27 | -0.62 | -0.49 | -0.74 |
| Qatar | 12.34 | 15.38 | 9.76 | 8.83 | 10.89 | 7.09 | 8.44 | 11.15 | 6.33 | -0.03 | 0.32 | -0.37 |
| Republic of Korea | 1020.31 | 1212.31 | 896.29 | 2.96 | 3.50 | 2.60 | 3.30 | 3.97 | 2.63 | 0.29 | 0.38 | 0.20 |
| Republic of Moldova | 544.42 | 598.90 | 486.48 | 12.30 | 13.52 | 11.01 | 10.05 | 11.78 | 8.55 | -0.23 | 0.01 | -0.46 |
| Romania | 2656.59 | 2861.33 | 2489.72 | 9.77 | 10.48 | 9.18 | 11.49 | 13.03 | 10.03 | 0.38 | 0.51 | 0.25 |
| Russian Federation | 17353.86 | 17797.12 | 16830.28 | 9.83 | 10.07 | 9.52 | 10.83 | 11.92 | 9.72 | -0.09 | 0.20 | -0.38 |
| Rwanda | 400.36 | 560.48 | 283.88 | 13.31 | 18.29 | 9.59 | 13.78 | 18.81 | 10.01 | -0.29 | -0.08 | -0.49 |
| Saint Kitts and Nevis | 8.13 | 8.88 | 7.48 | 23.53 | 25.58 | 21.69 | 16.42 | 19.23 | 13.64 | -0.59 | -0.39 | -0.79 |
| Saint Lucia | 14.11 | 15.17 | 13.05 | 17.13 | 18.36 | 15.92 | 12.57 | 15.02 | 10.49 | -1.33 | -1.06 | -1.61 |
| Saint Vincent and the Grenadines | 11.41 | 12.32 | 10.30 | 16.82 | 18.15 | 15.14 | 15.37 | 17.52 | 13.46 | -0.26 | -0.02 | -0.50 |
| Samoa | 7.71 | 9.91 | 5.80 | 9.12 | 11.50 | 6.93 | 10.90 | 14.44 | 8.13 | 0.57 | 0.63 | 0.51 |
| San Marino | 3.83 | 4.83 | 2.94 | 11.11 | 13.90 | 8.57 | 5.87 | 8.49 | 3.51 | -1.37 | -1.11 | -1.63 |
| Sao Tome and Principe | 3.78 | 4.62 | 3.07 | 6.00 | 7.35 | 4.94 | 9.02 | 11.60 | 6.90 | 1.30 | 1.38 | 1.22 |
| Saudi Arabia | 207.95 | 282.62 | 151.03 | 2.87 | 3.78 | 2.17 | 4.05 | 5.35 | 3.08 | 1.09 | 1.40 | 0.78 |
| Senegal | 201.81 | 253.10 | 159.15 | 5.97 | 7.40 | 4.76 | 8.99 | 11.80 | 6.86 | 1.37 | 1.49 | 1.26 |
| Serbia | 1644.14 | 2105.06 | 1243.71 | 15.75 | 20.27 | 11.87 | 14.63 | 18.80 | 11.16 | -0.56 | -0.41 | -0.70 |
| Seychelles | 4.84 | 5.58 | 4.19 | 8.65 | 9.95 | 7.50 | 11.48 | 13.37 | 9.75 | 0.88 | 1.22 | 0.54 |
| Sierra Leone | 108.04 | 142.71 | 77.99 | 5.17 | 6.70 | 3.82 | 7.62 | 10.11 | 5.61 | 1.50 | 1.59 | 1.41 |
| Singapore | 196.37 | 208.62 | 185.12 | 7.95 | 8.45 | 7.46 | 5.60 | 6.05 | 5.04 | -0.91 | -0.75 | -1.07 |
| Slovakia | 701.74 | 826.84 | 601.15 | 11.99 | 14.14 | 10.27 | 11.21 | 14.15 | 8.46 | -0.29 | -0.19 | -0.40 |
| Slovenia | 319.93 | 348.32 | 293.77 | 13.10 | 14.25 | 12.01 | 9.14 | 10.94 | 7.51 | -1.43 | -1.20 | -1.65 |
| Solomon Islands | 9.61 | 13.80 | 5.98 | 6.54 | 9.21 | 4.37 | 11.08 | 15.09 | 7.88 | 1.77 | 1.87 | 1.66 |
| Somalia | 216.37 | 299.44 | 147.40 | 7.76 | 10.52 | 5.46 | 8.58 | 11.62 | 5.91 | 0.35 | 0.40 | 0.31 |
| South Africa | 2166.76 | 2610.41 | 1733.35 | 10.12 | 12.32 | 7.95 | 14.36 | 15.86 | 12.98 | 1.46 | 1.66 | 1.26 |
| South Sudan | 180.47 | 256.34 | 129.98 | 7.01 | 9.86 | 5.09 | 8.71 | 12.03 | 6.30 | 0.64 | 0.85 | 0.42 |
| Spain | 6748.12 | 7233.36 | 6183.52 | 13.23 | 14.10 | 12.17 | 7.29 | 8.15 | 6.36 | -1.96 | -1.90 | -2.02 |
| Sri Lanka | 629.40 | 748.69 | 524.82 | 5.48 | 6.60 | 4.53 | 6.69 | 8.98 | 4.41 | 0.88 | 1.04 | 0.72 |
| Sudan | 279.51 | 423.46 | 177.30 | 2.73 | 4.01 | 1.81 | 3.90 | 6.00 | 2.61 | 1.26 | 1.43 | 1.09 |
| Suriname | 23.68 | 27.46 | 20.12 | 8.99 | 10.39 | 7.64 | 9.73 | 12.37 | 7.47 | 0.52 | 0.65 | 0.38 |
| Sweden | 1669.15 | 1789.20 | 1535.03 | 11.98 | 12.80 | 11.10 | 7.31 | 8.34 | 6.09 | -1.32 | -1.11 | -1.54 |
| Switzerland | 1426.61 | 1521.89 | 1313.39 | 14.07 | 14.96 | 13.07 | 7.90 | 8.76 | 6.79 | -1.83 | -1.67 | -1.99 |
| Syrian Arab Republic | 255.94 | 319.21 | 194.95 | 4.30 | 5.36 | 3.34 | 5.46 | 7.26 | 4.01 | 0.62 | 0.76 | 0.48 |
| Taiwan (Province of China) | 781.54 | 823.03 | 738.53 | 4.61 | 4.85 | 4.35 | 7.04 | 7.66 | 6.40 | 1.45 | 1.65 | 1.24 |
| Tajikistan | 218.28 | 265.32 | 175.32 | 7.62 | 9.29 | 6.09 | 5.44 | 7.71 | 3.61 | -1.22 | -1.10 | -1.34 |
| Thailand | 2371.32 | 2825.72 | 1921.79 | 5.89 | 7.06 | 4.74 | 8.62 | 10.90 | 6.48 | 1.31 | 1.54 | 1.08 |
| Timor-Leste | 16.19 | 24.75 | 10.17 | 4.81 | 7.17 | 3.18 | 6.88 | 9.36 | 4.76 | 1.28 | 1.47 | 1.08 |
| Togo | 97.69 | 122.80 | 79.18 | 7.03 | 8.71 | 5.77 | 10.02 | 13.52 | 7.32 | 1.16 | 1.23 | 1.10 |
| Tokelau | 0.18 | 0.26 | 0.12 | 14.50 | 20.44 | 9.83 | 16.60 | 21.90 | 12.17 | 0.40 | 0.44 | 0.36 |
| Tonga | 9.89 | 12.42 | 7.92 | 17.60 | 21.84 | 14.14 | 20.12 | 26.55 | 14.90 | 0.37 | 0.43 | 0.31 |
| Trinidad and Tobago | 119.39 | 128.56 | 111.05 | 14.69 | 15.74 | 13.73 | 13.68 | 17.37 | 10.58 | -0.20 | -0.02 | -0.38 |
| Tunisia | 244.97 | 295.46 | 199.62 | 4.86 | 5.88 | 3.96 | 5.85 | 8.00 | 4.19 | 0.48 | 0.57 | 0.39 |
| Turkey | 1117.95 | 1383.18 | 890.27 | 2.96 | 3.63 | 2.36 | 6.81 | 8.45 | 5.32 | 3.97 | 4.71 | 3.24 |
| Turkmenistan | 156.85 | 178.08 | 136.87 | 7.70 | 8.71 | 6.73 | 6.75 | 8.94 | 5.07 | 0.01 | 0.46 | -0.44 |
| Tuvalu | 0.85 | 1.23 | 0.56 | 13.16 | 18.62 | 8.92 | 15.16 | 20.28 | 11.21 | 0.41 | 0.48 | 0.34 |
| Uganda | 660.69 | 878.99 | 481.39 | 10.12 | 13.28 | 7.55 | 15.50 | 20.88 | 11.81 | 0.87 | 1.13 | 0.61 |
| Ukraine | 9738.11 | 10412.35 | 9064.58 | 14.08 | 15.03 | 13.13 | 10.18 | 14.73 | 6.61 | -1.54 | -1.31 | -1.77 |
| United Arab Emirates | 44.10 | 58.66 | 32.53 | 8.31 | 10.63 | 6.37 | 8.90 | 11.70 | 6.53 | 2.03 | 2.65 | 1.41 |
| United Kingdom | 17643.67 | 18138.86 | 16628.80 | 20.69 | 21.26 | 19.62 | 10.62 | 11.16 | 9.63 | -2.27 | -2.19 | -2.36 |
| United Republic of Tanzania | 1051.39 | 1285.55 | 848.16 | 9.70 | 11.81 | 7.93 | 12.23 | 16.07 | 9.30 | 0.72 | 0.77 | 0.66 |
| United States of America | 49187.61 | 50998.79 | 45894.16 | 15.94 | 16.51 | 14.95 | 9.41 | 9.94 | 8.55 | -1.85 | -1.79 | -1.91 |
| United States Virgin Islands | 14.27 | 16.99 | 11.98 | 16.91 | 20.17 | 14.30 | 11.70 | 16.14 | 8.21 | -0.88 | -0.73 | -1.03 |
| Uruguay | 755.37 | 799.42 | 708.10 | 20.14 | 21.30 | 18.90 | 16.85 | 18.44 | 15.12 | -0.74 | -0.64 | -0.84 |
| Uzbekistan | 896.12 | 993.41 | 797.25 | 7.54 | 8.39 | 6.68 | 6.16 | 7.38 | 5.14 | -0.53 | -0.32 | -0.74 |
| Vanuatu | 4.60 | 6.49 | 3.17 | 6.90 | 9.54 | 4.98 | 10.88 | 13.72 | 8.17 | 1.33 | 1.42 | 1.23 |
| Venezuela (Bolivarian Republic of) | 788.56 | 834.78 | 745.74 | 7.47 | 7.90 | 7.04 | 10.69 | 13.51 | 8.23 | 1.02 | 1.17 | 0.87 |
| Viet Nam | 1997.73 | 2586.02 | 1555.69 | 4.91 | 6.33 | 3.85 | 6.23 | 8.08 | 4.74 | 0.83 | 0.86 | 0.79 |
| Yemen | 130.67 | 184.18 | 82.08 | 2.44 | 3.37 | 1.63 | 3.94 | 5.46 | 2.80 | 1.70 | 1.83 | 1.58 |
| Zambia | 286.73 | 394.85 | 208.65 | 9.24 | 12.29 | 6.94 | 16.97 | 24.81 | 10.99 | 2.15 | 2.44 | 1.85 |
| Zimbabwe | 358.93 | 462.03 | 276.78 | 8.99 | 11.49 | 7.03 | 17.01 | 22.42 | 12.69 | 2.89 | 3.54 | 2.23 |

EAPC: estimated annual percentage change; UI: uncertainty interval; CI: confidence interval.

Table S4. The all-ages numbers and the age-standardized rates of prevalence, and its corresponding EAPC of breast cancer among 204 countries in 1990 and 2021

| Characteristics | Counts (2021) | | | Age-standardized Prevalence rate (per 100,000) (95% UI) | | | | | | EAPC (95% CI)1990–2021 | | |
| --- | --- | --- | --- | --- | --- | --- | --- | --- | --- | --- | --- | --- |
|  |  |  |  | 1990 | | | 2021 | | |  |  |  |
|  | val | upper | lower | val | upper | lower | val | upper | lower | val | upper | lower |
| Afghanistan | 16322.28 | 28505.18 | 8781.83 | 67.82 | 100.31 | 44.55 | 116.26 | 187.98 | 70.89 | 2.00 | 2.29 | 1.70 |
| Albania | 6222.76 | 7793.67 | 4883.07 | 103.06 | 125.80 | 85.34 | 159.05 | 201.13 | 124.01 | 2.26 | 2.49 | 2.02 |
| Algeria | 56710.01 | 72733.09 | 44150.29 | 86.73 | 105.40 | 70.99 | 139.05 | 175.63 | 110.23 | 1.80 | 1.99 | 1.60 |
| American Samoa | 143.02 | 171.51 | 118.30 | 178.52 | 208.16 | 149.70 | 278.21 | 333.37 | 230.93 | 1.62 | 1.71 | 1.53 |
| Andorra | 706.16 | 891.75 | 548.17 | 415.21 | 553.50 | 324.89 | 476.20 | 608.03 | 363.99 | 0.94 | 1.14 | 0.75 |
| Angola | 17024.92 | 22376.60 | 12158.48 | 60.01 | 77.66 | 46.47 | 109.07 | 141.17 | 80.44 | 2.12 | 2.31 | 1.94 |
| Antigua and Barbuda | 426.63 | 461.87 | 395.19 | 264.08 | 290.35 | 237.61 | 381.00 | 411.57 | 354.20 | 1.42 | 1.59 | 1.26 |
| Argentina | 152826.27 | 163086.49 | 142791.84 | 254.83 | 280.63 | 233.13 | 281.63 | 300.75 | 262.06 | 0.44 | 0.58 | 0.29 |
| Armenia | 11577.52 | 12886.34 | 10422.72 | 280.29 | 302.72 | 259.37 | 270.28 | 301.23 | 243.33 | -0.09 | 0.18 | -0.37 |
| Australia | 185104.68 | 201520.51 | 169174.21 | 447.56 | 491.05 | 411.20 | 457.66 | 499.32 | 418.55 | 0.19 | 0.35 | 0.03 |
| Austria | 60223.56 | 64960.17 | 55402.91 | 460.44 | 504.84 | 421.41 | 374.71 | 404.03 | 346.89 | -0.51 | -0.41 | -0.60 |
| Azerbaijan | 18801.04 | 23362.54 | 14865.82 | 148.89 | 167.16 | 128.71 | 159.61 | 196.02 | 128.15 | 0.28 | 0.44 | 0.12 |
| Bahamas | 1938.70 | 2357.10 | 1612.60 | 328.63 | 358.74 | 301.84 | 439.90 | 531.69 | 367.72 | 1.24 | 1.39 | 1.09 |
| Bahrain | 4927.70 | 6138.81 | 3969.12 | 226.20 | 259.60 | 197.48 | 416.63 | 511.33 | 340.67 | 1.88 | 2.06 | 1.71 |
| Bangladesh | 115490.12 | 142931.36 | 90587.36 | 33.54 | 43.30 | 26.86 | 73.72 | 91.13 | 58.30 | 2.51 | 2.67 | 2.35 |
| Barbados | 2153.65 | 2663.52 | 1756.31 | 331.60 | 360.13 | 304.02 | 454.62 | 561.12 | 368.33 | 1.39 | 1.58 | 1.20 |
| Belarus | 43345.89 | 51842.42 | 35842.42 | 237.06 | 263.58 | 213.11 | 286.64 | 346.68 | 234.29 | 0.12 | 0.29 | -0.05 |
| Belgium | 99189.92 | 106068.84 | 91373.00 | 560.24 | 611.82 | 515.62 | 482.65 | 517.11 | 446.62 | -0.49 | -0.29 | -0.68 |
| Belize | 467.93 | 526.58 | 412.70 | 84.99 | 93.63 | 76.56 | 134.27 | 150.53 | 119.15 | 1.67 | 1.79 | 1.56 |
| Benin | 4778.21 | 6234.48 | 3524.22 | 55.63 | 64.90 | 46.44 | 75.23 | 96.15 | 57.60 | 0.97 | 1.05 | 0.88 |
| Bermuda | 536.43 | 668.01 | 452.52 | 425.21 | 471.22 | 381.87 | 452.37 | 562.91 | 377.16 | -0.19 | -0.05 | -0.34 |
| Bhutan | 425.36 | 571.41 | 311.65 | 39.06 | 48.64 | 30.75 | 62.68 | 83.10 | 46.91 | 1.62 | 1.78 | 1.45 |
| Bolivia (Plurinational State of) | 13391.71 | 18855.52 | 9173.07 | 80.86 | 110.97 | 56.72 | 132.61 | 186.74 | 91.71 | 1.61 | 1.64 | 1.59 |
| Bosnia and Herzegovina | 14269.75 | 17008.78 | 11806.18 | 163.21 | 187.02 | 141.50 | 247.16 | 297.80 | 202.09 | 2.16 | 2.41 | 1.92 |
| Botswana | 2309.40 | 3257.89 | 1636.39 | 91.24 | 119.12 | 68.64 | 130.44 | 174.66 | 98.01 | 1.61 | 1.97 | 1.26 |
| Brazil | 508143.62 | 534889.08 | 480596.83 | 124.51 | 131.41 | 118.35 | 197.45 | 207.88 | 186.47 | 1.31 | 1.38 | 1.23 |
| Brunei Darussalam | 1067.38 | 1280.23 | 871.27 | 170.69 | 209.17 | 136.48 | 243.35 | 286.60 | 202.12 | 1.71 | 1.92 | 1.50 |
| Bulgaria | 47456.13 | 54569.35 | 40310.41 | 281.21 | 313.51 | 250.59 | 382.03 | 446.91 | 322.13 | 1.18 | 1.29 | 1.08 |
| Burkina Faso | 13378.25 | 17538.81 | 9772.49 | 90.56 | 109.89 | 73.28 | 116.89 | 147.96 | 89.61 | 0.89 | 0.96 | 0.82 |
| Burundi | 4691.76 | 6187.45 | 3543.92 | 77.63 | 104.99 | 59.67 | 74.36 | 96.23 | 58.29 | -0.36 | -0.22 | -0.50 |
| Cabo Verde | 606.57 | 762.44 | 480.95 | 99.77 | 118.86 | 82.18 | 124.27 | 155.39 | 100.15 | 0.90 | 1.14 | 0.65 |
| Cambodia | 21216.58 | 27791.70 | 16011.68 | 77.31 | 106.88 | 56.02 | 148.49 | 191.50 | 113.48 | 2.31 | 2.39 | 2.22 |
| Cameroon | 16492.76 | 22507.98 | 12151.01 | 72.10 | 85.89 | 59.88 | 102.02 | 135.54 | 77.68 | 1.03 | 1.20 | 0.87 |
| Canada | 285912.79 | 309423.97 | 264124.68 | 542.07 | 589.41 | 500.10 | 441.96 | 477.29 | 409.06 | -0.72 | -0.60 | -0.85 |
| Central African Republic | 2355.91 | 3225.92 | 1664.84 | 71.00 | 91.36 | 54.31 | 83.02 | 107.14 | 62.30 | 0.49 | 0.60 | 0.39 |
| Chad | 4173.39 | 5432.08 | 3082.68 | 46.15 | 57.06 | 36.15 | 57.16 | 72.96 | 43.96 | 0.84 | 0.97 | 0.72 |
| Chile | 49910.53 | 53731.13 | 46190.72 | 165.82 | 185.69 | 149.96 | 201.99 | 217.17 | 186.81 | 1.08 | 1.21 | 0.95 |
| China | 3893548.90 | 4736669.32 | 3168427.28 | 89.45 | 105.04 | 76.11 | 185.68 | 226.99 | 150.84 | 2.62 | 2.74 | 2.51 |
| Colombia | 165477.43 | 197451.80 | 139186.01 | 149.39 | 161.25 | 137.82 | 301.12 | 359.58 | 253.56 | 2.29 | 2.45 | 2.13 |
| Comoros | 762.54 | 974.84 | 596.53 | 84.87 | 108.90 | 65.96 | 134.30 | 170.38 | 106.88 | 1.37 | 1.45 | 1.29 |
| Congo | 5996.23 | 9317.66 | 3771.07 | 106.24 | 156.03 | 72.52 | 166.05 | 249.12 | 110.76 | 1.44 | 1.58 | 1.29 |
| Cook Islands | 95.83 | 119.68 | 76.59 | 267.28 | 333.84 | 212.17 | 406.90 | 514.29 | 320.88 | 1.32 | 1.43 | 1.22 |
| Costa Rica | 20978.17 | 23794.31 | 18470.26 | 191.36 | 208.10 | 176.33 | 382.16 | 432.30 | 336.38 | 2.31 | 2.45 | 2.17 |
| Croatia | 27715.74 | 31020.55 | 24349.10 | 318.23 | 357.55 | 281.08 | 353.11 | 399.36 | 305.84 | 0.49 | 0.64 | 0.34 |
| Cuba | 51128.27 | 59525.24 | 44294.73 | 222.63 | 239.65 | 206.58 | 279.63 | 328.01 | 243.05 | 0.77 | 0.86 | 0.67 |
| Cyprus | 10283.74 | 11769.14 | 8808.03 | 369.67 | 433.43 | 316.00 | 526.51 | 602.47 | 450.05 | 1.67 | 1.91 | 1.42 |
| Czechia | 60285.77 | 67979.54 | 52135.40 | 301.21 | 334.40 | 270.61 | 314.23 | 357.59 | 270.06 | -0.05 | 0.20 | -0.29 |
| Côte d'Ivoire | 17258.26 | 22902.44 | 12666.56 | 74.08 | 91.26 | 60.73 | 120.54 | 155.66 | 92.88 | 1.73 | 1.81 | 1.66 |
| Democratic People's Republic of Korea | 40717.70 | 52953.49 | 30000.67 | 86.99 | 115.11 | 65.63 | 119.55 | 154.64 | 88.80 | 1.38 | 1.54 | 1.22 |
| Democratic Republic of the Congo | 44812.08 | 59193.75 | 34291.30 | 66.26 | 85.23 | 50.87 | 97.45 | 126.00 | 75.67 | 1.28 | 1.56 | 0.99 |
| Denmark | 43751.20 | 47094.34 | 40700.23 | 505.37 | 547.68 | 465.73 | 414.11 | 445.51 | 386.41 | -0.43 | -0.20 | -0.66 |
| Djibouti | 1031.02 | 1492.39 | 716.39 | 86.79 | 109.38 | 68.15 | 121.20 | 168.43 | 88.25 | 1.04 | 1.14 | 0.95 |
| Dominica | 216.93 | 266.36 | 170.24 | 230.14 | 263.54 | 200.63 | 262.31 | 321.06 | 207.40 | 0.43 | 0.49 | 0.38 |
| Dominican Republic | 14121.87 | 17870.85 | 11137.91 | 90.63 | 102.94 | 77.93 | 134.41 | 169.98 | 106.86 | 1.60 | 1.77 | 1.44 |
| Ecuador | 22555.80 | 27951.83 | 17665.94 | 62.75 | 68.09 | 57.93 | 132.71 | 164.27 | 104.18 | 2.71 | 2.92 | 2.49 |
| Egypt | 180028.29 | 217820.63 | 146474.43 | 97.00 | 112.04 | 84.09 | 235.78 | 281.39 | 197.07 | 3.38 | 3.64 | 3.13 |
| El Salvador | 14012.96 | 17454.78 | 11179.07 | 81.36 | 91.72 | 71.32 | 231.37 | 288.15 | 184.30 | 3.65 | 3.88 | 3.41 |
| Equatorial Guinea | 1269.82 | 1991.17 | 779.56 | 72.35 | 95.39 | 54.21 | 184.10 | 275.16 | 119.61 | 3.48 | 3.63 | 3.33 |
| Eritrea | 4704.63 | 6285.49 | 3414.98 | 86.24 | 111.15 | 68.71 | 130.99 | 168.63 | 99.59 | 1.35 | 1.38 | 1.32 |
| Estonia | 7211.82 | 8189.55 | 6207.46 | 280.87 | 307.82 | 254.73 | 300.63 | 341.53 | 256.59 | 0.22 | 0.31 | 0.12 |
| Eswatini | 1055.42 | 1579.63 | 656.29 | 94.34 | 113.39 | 76.04 | 158.22 | 231.44 | 104.04 | 1.93 | 2.05 | 1.82 |
| Ethiopia | 57919.28 | 70350.72 | 47593.58 | 74.61 | 102.14 | 54.40 | 104.09 | 125.03 | 88.05 | 1.00 | 1.21 | 0.79 |
| Fiji | 1969.45 | 2481.93 | 1525.81 | 197.18 | 238.96 | 163.00 | 231.71 | 287.75 | 181.87 | 0.37 | 0.51 | 0.23 |
| Finland | 50156.94 | 54065.35 | 45714.06 | 448.05 | 495.19 | 407.35 | 468.05 | 506.18 | 430.11 | 0.43 | 0.60 | 0.25 |
| France | 679458.73 | 729277.29 | 624121.63 | 474.42 | 518.89 | 432.88 | 582.38 | 625.97 | 536.78 | 0.98 | 1.12 | 0.83 |
| Gabon | 2179.54 | 2950.67 | 1554.66 | 109.15 | 140.81 | 83.15 | 175.98 | 233.65 | 128.86 | 1.42 | 1.52 | 1.33 |
| Gambia | 682.58 | 889.94 | 519.13 | 33.99 | 41.52 | 27.20 | 57.74 | 73.87 | 44.85 | 1.77 | 2.00 | 1.55 |
| Georgia | 18059.92 | 20196.50 | 16054.16 | 288.92 | 315.86 | 258.86 | 328.48 | 368.79 | 291.69 | 0.42 | 0.63 | 0.21 |
| Germany | 804903.60 | 855720.41 | 744049.79 | 454.99 | 502.66 | 418.70 | 482.06 | 510.91 | 448.72 | 0.41 | 0.55 | 0.27 |
| Ghana | 27931.06 | 36402.58 | 21555.38 | 88.64 | 108.98 | 71.61 | 133.14 | 169.35 | 105.24 | 1.18 | 1.25 | 1.11 |
| Greece | 94146.64 | 100391.34 | 87575.41 | 474.10 | 520.52 | 436.33 | 470.74 | 503.88 | 439.56 | -0.08 | 0.00 | -0.16 |
| Greenland | 151.77 | 187.94 | 121.02 | 260.31 | 305.74 | 219.62 | 212.08 | 259.81 | 170.93 | -0.68 | -0.59 | -0.78 |
| Grenada | 362.37 | 412.65 | 316.92 | 218.75 | 242.39 | 197.34 | 308.90 | 350.62 | 270.91 | 1.25 | 1.44 | 1.05 |
| Guam | 315.38 | 362.23 | 278.10 | 141.64 | 161.70 | 123.78 | 160.09 | 182.68 | 140.43 | 0.78 | 0.96 | 0.60 |
| Guatemala | 13194.65 | 15479.76 | 11234.10 | 46.14 | 50.52 | 42.19 | 107.97 | 126.53 | 92.30 | 3.02 | 3.25 | 2.79 |
| Guinea | 5726.67 | 7807.58 | 4188.70 | 57.06 | 69.16 | 45.42 | 85.33 | 113.48 | 64.43 | 1.34 | 1.40 | 1.29 |
| Guinea-Bissau | 1034.45 | 1368.62 | 759.40 | 68.14 | 90.57 | 51.94 | 104.24 | 134.77 | 78.51 | 1.43 | 1.47 | 1.38 |
| Guyana | 1413.59 | 1824.94 | 1087.23 | 128.79 | 147.25 | 113.90 | 197.64 | 252.83 | 153.35 | 1.72 | 1.94 | 1.51 |
| Haiti | 12539.00 | 19234.87 | 8080.04 | 105.42 | 153.41 | 71.44 | 137.73 | 208.04 | 90.83 | 1.09 | 1.20 | 0.99 |
| Honduras | 9742.78 | 13200.73 | 7150.72 | 66.95 | 83.11 | 51.90 | 134.49 | 180.39 | 100.08 | 2.38 | 2.46 | 2.30 |
| Hungary | 59731.00 | 67452.52 | 52590.09 | 298.63 | 336.98 | 265.43 | 345.20 | 394.82 | 300.54 | 0.26 | 0.46 | 0.07 |
| Iceland | 2381.42 | 2594.84 | 2165.66 | 496.03 | 545.01 | 448.29 | 450.51 | 492.14 | 408.07 | -0.36 | -0.25 | -0.46 |
| India | 1267815.40 | 1464295.32 | 1102428.14 | 47.66 | 54.11 | 41.98 | 95.64 | 110.69 | 83.28 | 2.37 | 2.57 | 2.17 |
| Indonesia | 406686.71 | 553103.49 | 294172.17 | 84.80 | 111.80 | 65.35 | 141.79 | 191.30 | 104.33 | 1.64 | 1.68 | 1.60 |
| Iran (Islamic Republic of) | 237390.48 | 264064.92 | 215254.32 | 110.28 | 128.26 | 95.74 | 257.13 | 284.33 | 233.32 | 3.25 | 3.57 | 2.93 |
| Iraq | 86035.22 | 113183.40 | 63151.47 | 131.02 | 165.75 | 105.13 | 284.62 | 370.57 | 212.57 | 2.76 | 2.92 | 2.60 |
| Ireland | 35562.92 | 38323.21 | 32726.84 | 498.70 | 544.17 | 456.56 | 483.96 | 521.11 | 444.98 | 0.21 | 0.36 | 0.07 |
| Israel | 45861.42 | 49688.14 | 42390.44 | 427.18 | 470.00 | 392.26 | 399.73 | 433.76 | 370.08 | -0.15 | 0.04 | -0.34 |
| Italy | 602228.22 | 646331.33 | 552431.83 | 524.47 | 572.03 | 481.93 | 497.81 | 532.77 | 463.06 | -0.19 | -0.03 | -0.34 |
| Jamaica | 10361.44 | 13287.97 | 8074.19 | 199.19 | 213.85 | 183.52 | 337.80 | 432.99 | 262.73 | 1.79 | 2.02 | 1.55 |
| Japan | 936022.08 | 1010572.85 | 849337.53 | 220.82 | 245.94 | 200.63 | 357.74 | 381.88 | 331.66 | 1.83 | 1.95 | 1.70 |
| Jordan | 28006.74 | 36465.54 | 20822.09 | 167.27 | 208.91 | 136.05 | 305.35 | 394.74 | 231.76 | 2.41 | 2.76 | 2.06 |
| Kazakhstan | 33642.00 | 38366.84 | 29310.17 | 197.92 | 219.21 | 177.79 | 174.58 | 198.30 | 152.76 | -0.05 | 0.06 | -0.16 |
| Kenya | 36342.83 | 48896.34 | 26549.58 | 67.78 | 86.12 | 52.55 | 125.24 | 165.70 | 93.69 | 2.12 | 2.30 | 1.93 |
| Kiribati | 139.20 | 181.19 | 109.87 | 121.93 | 144.60 | 100.99 | 160.72 | 204.94 | 128.92 | 1.07 | 1.12 | 1.01 |
| Kuwait | 11733.95 | 13551.94 | 10229.19 | 160.73 | 179.88 | 141.99 | 272.54 | 310.23 | 238.32 | 1.61 | 2.04 | 1.19 |
| Kyrgyzstan | 7486.25 | 8801.29 | 6296.98 | 155.09 | 173.65 | 137.52 | 134.72 | 157.11 | 114.86 | -0.63 | -0.46 | -0.80 |
| Lao People's Democratic Republic | 6338.87 | 8282.86 | 4724.76 | 67.88 | 99.24 | 46.43 | 111.32 | 144.46 | 84.39 | 1.85 | 1.95 | 1.76 |
| Latvia | 10359.79 | 11817.82 | 8904.08 | 259.29 | 289.13 | 227.97 | 290.64 | 336.62 | 246.06 | 0.32 | 0.46 | 0.19 |
| Lebanon | 29624.56 | 35333.76 | 24587.16 | 224.02 | 288.87 | 174.73 | 501.65 | 600.63 | 415.96 | 3.11 | 3.36 | 2.86 |
| Lesotho | 1626.34 | 2267.39 | 1102.63 | 80.23 | 103.69 | 62.29 | 136.99 | 187.88 | 96.48 | 2.41 | 2.67 | 2.16 |
| Liberia | 2564.59 | 3638.36 | 1819.67 | 49.87 | 60.26 | 40.53 | 88.06 | 117.85 | 65.12 | 2.08 | 2.25 | 1.92 |
| Libya | 14676.04 | 19857.92 | 11159.78 | 105.11 | 125.87 | 87.95 | 225.47 | 292.90 | 176.98 | 2.92 | 3.19 | 2.66 |
| Lithuania | 14944.45 | 16832.66 | 13114.90 | 266.59 | 291.51 | 244.46 | 300.42 | 337.80 | 261.77 | 0.29 | 0.43 | 0.16 |
| Luxembourg | 4158.15 | 4513.20 | 3803.84 | 484.04 | 529.95 | 441.00 | 412.15 | 447.37 | 377.38 | -0.14 | 0.07 | -0.35 |
| Madagascar | 15215.11 | 20307.78 | 11347.78 | 77.76 | 93.69 | 65.16 | 98.47 | 126.81 | 75.05 | 0.65 | 0.83 | 0.48 |
| Malawi | 10068.22 | 13026.60 | 7450.18 | 63.91 | 76.42 | 52.76 | 107.20 | 137.14 | 81.91 | 1.80 | 1.98 | 1.62 |
| Malaysia | 81970.16 | 95020.75 | 70217.74 | 134.86 | 157.16 | 116.41 | 261.91 | 302.41 | 225.28 | 2.22 | 2.31 | 2.13 |
| Maldives | 363.06 | 452.20 | 287.08 | 51.52 | 78.23 | 32.51 | 83.10 | 100.51 | 68.00 | 1.70 | 2.04 | 1.35 |
| Mali | 9592.14 | 12799.59 | 7170.88 | 65.87 | 77.51 | 55.59 | 89.02 | 116.09 | 67.97 | 0.91 | 0.98 | 0.84 |
| Malta | 3632.67 | 3996.83 | 3325.75 | 456.76 | 504.20 | 417.94 | 440.16 | 484.81 | 402.15 | -0.15 | -0.04 | -0.27 |
| Marshall Islands | 74.66 | 110.97 | 48.50 | 120.64 | 157.12 | 94.02 | 164.70 | 237.77 | 112.78 | 1.02 | 1.06 | 0.98 |
| Mauritania | 2930.62 | 3790.63 | 2271.08 | 69.07 | 88.44 | 53.63 | 117.53 | 150.23 | 92.33 | 1.62 | 1.79 | 1.46 |
| Mauritius | 4394.52 | 4688.75 | 4022.94 | 99.94 | 109.79 | 90.82 | 244.77 | 261.45 | 223.13 | 2.55 | 2.83 | 2.28 |
| Mexico | 307231.10 | 359389.08 | 260492.67 | 121.95 | 128.90 | 115.92 | 226.18 | 263.72 | 192.09 | 1.74 | 1.87 | 1.61 |
| Micronesia (Federated States of) | 165.77 | 213.03 | 125.73 | 137.24 | 179.91 | 105.10 | 193.74 | 245.43 | 149.08 | 1.16 | 1.20 | 1.13 |
| Monaco | 672.03 | 832.44 | 551.29 | 631.63 | 780.29 | 511.42 | 886.30 | 1127.15 | 708.72 | 1.33 | 1.45 | 1.20 |
| Mongolia | 1581.22 | 1887.96 | 1312.22 | 45.74 | 54.58 | 37.76 | 55.33 | 65.62 | 46.25 | 0.91 | 1.11 | 0.71 |
| Montenegro | 4084.55 | 4951.86 | 3371.61 | 344.79 | 424.56 | 280.97 | 438.93 | 530.89 | 361.35 | 1.23 | 1.33 | 1.13 |
| Morocco | 50206.48 | 70547.47 | 36055.60 | 63.52 | 76.51 | 52.71 | 132.50 | 183.87 | 96.54 | 2.76 | 3.01 | 2.51 |
| Mozambique | 14806.72 | 19245.13 | 10688.72 | 66.57 | 78.80 | 56.89 | 104.42 | 132.30 | 78.55 | 1.72 | 1.89 | 1.54 |
| Myanmar | 79434.44 | 100271.44 | 62930.91 | 94.07 | 125.35 | 70.29 | 145.05 | 181.73 | 116.36 | 1.37 | 1.41 | 1.33 |
| Namibia | 3629.02 | 5084.36 | 2484.07 | 101.27 | 117.84 | 86.91 | 223.75 | 304.97 | 158.02 | 3.02 | 3.18 | 2.86 |
| Nauru | 17.62 | 25.86 | 11.49 | 156.29 | 224.97 | 106.00 | 242.78 | 347.59 | 165.23 | 1.35 | 1.43 | 1.27 |
| Nepal | 17111.64 | 22540.35 | 12984.63 | 38.11 | 46.48 | 30.50 | 65.90 | 86.23 | 50.48 | 1.89 | 2.15 | 1.63 |
| Netherlands | 155787.08 | 166634.90 | 144227.22 | 512.13 | 563.21 | 472.30 | 509.74 | 543.65 | 475.03 | 0.17 | 0.35 | 0.00 |
| New Zealand | 38786.60 | 41872.86 | 35517.32 | 590.88 | 652.82 | 542.17 | 505.24 | 544.64 | 464.39 | -0.38 | -0.33 | -0.44 |
| Nicaragua | 8246.18 | 10163.15 | 6649.46 | 70.75 | 81.37 | 61.14 | 149.55 | 183.27 | 121.70 | 2.84 | 3.01 | 2.68 |
| Niger | 4784.63 | 6407.05 | 3472.28 | 38.94 | 48.01 | 31.71 | 48.11 | 63.25 | 35.70 | 0.82 | 0.93 | 0.72 |
| Nigeria | 182778.47 | 259883.47 | 125815.99 | 78.33 | 97.38 | 61.91 | 161.87 | 223.27 | 117.86 | 2.59 | 2.88 | 2.30 |
| Niue | 5.24 | 6.61 | 4.15 | 189.64 | 235.43 | 152.61 | 257.22 | 323.52 | 204.88 | 0.73 | 0.81 | 0.66 |
| North Macedonia | 10487.68 | 12883.15 | 8542.62 | 244.79 | 282.49 | 206.79 | 323.14 | 396.78 | 263.46 | 1.22 | 1.40 | 1.03 |
| Northern Mariana Islands | 140.79 | 160.35 | 119.19 | 183.84 | 232.58 | 144.98 | 244.49 | 275.04 | 209.73 | 0.56 | 0.71 | 0.42 |
| Norway | 31780.35 | 34275.62 | 29429.78 | 378.53 | 416.55 | 347.77 | 353.15 | 379.52 | 329.06 | -0.12 | 0.16 | -0.39 |
| Oman | 2281.18 | 2883.29 | 1768.21 | 53.60 | 65.52 | 43.75 | 88.01 | 107.53 | 71.82 | 2.09 | 2.29 | 1.89 |
| Pakistan | 253476.71 | 329179.93 | 188056.25 | 89.45 | 106.04 | 74.49 | 160.80 | 204.41 | 121.63 | 1.65 | 1.76 | 1.54 |
| Palau | 64.30 | 79.71 | 51.46 | 262.35 | 328.45 | 213.56 | 276.94 | 340.09 | 225.06 | 0.16 | 0.23 | 0.08 |
| Palestine | 10879.30 | 13023.32 | 9073.24 | 201.94 | 259.66 | 156.13 | 356.42 | 421.21 | 298.94 | 2.05 | 2.17 | 1.93 |
| Panama | 14093.94 | 17013.09 | 11605.00 | 149.35 | 162.52 | 137.07 | 319.56 | 386.00 | 263.06 | 2.54 | 2.64 | 2.45 |
| Papua New Guinea | 7456.87 | 9872.66 | 5668.57 | 94.43 | 124.43 | 72.66 | 103.35 | 133.57 | 81.06 | 0.10 | 0.24 | -0.04 |
| Paraguay | 11583.88 | 14914.93 | 8886.32 | 100.27 | 117.97 | 84.62 | 184.71 | 236.70 | 142.46 | 2.02 | 2.14 | 1.90 |
| Peru | 53377.12 | 68740.93 | 39618.29 | 83.90 | 98.18 | 70.43 | 151.47 | 194.99 | 113.15 | 1.77 | 1.93 | 1.60 |
| Philippines | 169413.89 | 205583.42 | 139290.56 | 115.12 | 127.02 | 102.26 | 176.71 | 211.88 | 146.63 | 1.37 | 1.47 | 1.27 |
| Poland | 196861.71 | 215692.31 | 176919.86 | 215.44 | 235.45 | 197.80 | 303.80 | 334.53 | 272.16 | 1.30 | 1.43 | 1.18 |
| Portugal | 86041.53 | 91809.33 | 79480.39 | 385.72 | 427.60 | 351.61 | 432.87 | 462.76 | 399.35 | 0.56 | 0.72 | 0.41 |
| Puerto Rico | 19398.34 | 22571.78 | 16555.67 | 246.65 | 266.03 | 228.39 | 337.87 | 395.19 | 287.34 | 1.07 | 1.21 | 0.93 |
| Qatar | 5485.17 | 7339.90 | 4028.14 | 181.84 | 217.10 | 153.21 | 387.49 | 488.10 | 306.73 | 2.44 | 2.59 | 2.28 |
| Republic of Korea | 187733.33 | 213925.60 | 162212.90 | 109.73 | 129.35 | 93.16 | 218.87 | 250.52 | 189.72 | 2.94 | 3.20 | 2.68 |
| Republic of Moldova | 14787.82 | 17061.32 | 12801.51 | 226.35 | 254.37 | 200.96 | 256.55 | 296.86 | 221.90 | 0.54 | 0.73 | 0.35 |
| Romania | 89670.21 | 99415.89 | 80087.54 | 183.24 | 204.49 | 166.96 | 274.28 | 305.19 | 243.43 | 1.55 | 1.63 | 1.46 |
| Russian Federation | 702931.99 | 768999.45 | 636799.59 | 224.82 | 246.76 | 208.37 | 308.03 | 337.06 | 278.38 | 0.97 | 1.08 | 0.86 |
| Rwanda | 10376.95 | 13832.76 | 7585.12 | 100.44 | 135.05 | 75.50 | 133.62 | 175.41 | 100.05 | 0.76 | 0.98 | 0.53 |
| Saint Kitts and Nevis | 209.78 | 251.59 | 173.57 | 275.46 | 302.23 | 250.96 | 281.90 | 334.75 | 235.67 | 0.41 | 0.56 | 0.27 |
| Saint Lucia | 601.18 | 718.51 | 506.69 | 235.25 | 253.92 | 218.45 | 250.99 | 299.55 | 211.81 | 0.12 | 0.29 | -0.06 |
| Saint Vincent and the Grenadines | 393.30 | 449.26 | 345.75 | 238.82 | 260.96 | 216.32 | 279.13 | 319.43 | 244.36 | 0.43 | 0.61 | 0.25 |
| Samoa | 230.92 | 296.74 | 178.77 | 115.69 | 141.23 | 95.18 | 147.27 | 187.26 | 115.13 | 0.79 | 0.88 | 0.70 |
| San Marino | 206.70 | 269.20 | 156.63 | 394.42 | 474.42 | 324.04 | 330.39 | 444.76 | 239.96 | 0.17 | 0.41 | -0.07 |
| Sao Tome and Principe | 165.38 | 214.03 | 126.40 | 63.07 | 74.52 | 53.71 | 118.66 | 149.18 | 92.58 | 2.14 | 2.21 | 2.06 |
| Saudi Arabia | 55873.47 | 76374.69 | 41132.55 | 75.00 | 93.29 | 61.39 | 177.49 | 224.59 | 142.08 | 3.31 | 3.46 | 3.15 |
| Senegal | 9020.35 | 11894.93 | 7002.47 | 58.14 | 70.25 | 47.50 | 99.00 | 127.80 | 78.90 | 1.82 | 1.96 | 1.69 |
| Serbia | 54866.75 | 67166.31 | 44795.20 | 261.53 | 321.04 | 212.25 | 371.29 | 456.83 | 300.17 | 1.18 | 1.30 | 1.06 |
| Seychelles | 264.12 | 300.87 | 230.93 | 121.66 | 139.72 | 105.18 | 209.79 | 237.54 | 183.84 | 1.91 | 2.19 | 1.63 |
| Sierra Leone | 3692.73 | 4784.95 | 2693.55 | 48.39 | 60.20 | 37.88 | 78.05 | 98.82 | 59.16 | 1.78 | 1.91 | 1.66 |
| Singapore | 26964.53 | 28816.81 | 25113.21 | 239.52 | 267.11 | 217.25 | 311.07 | 332.09 | 289.71 | 1.27 | 1.45 | 1.09 |
| Slovakia | 28239.91 | 33388.29 | 23219.21 | 243.72 | 276.22 | 215.18 | 311.46 | 368.27 | 257.79 | 0.96 | 1.07 | 0.85 |
| Slovenia | 12488.70 | 14252.25 | 10968.66 | 311.30 | 347.18 | 280.66 | 318.56 | 369.50 | 276.24 | 0.21 | 0.42 | -0.01 |
| Solomon Islands | 529.27 | 714.62 | 392.34 | 72.89 | 94.97 | 52.49 | 114.97 | 151.11 | 87.27 | 1.60 | 1.81 | 1.40 |
| Somalia | 5569.24 | 7552.94 | 3868.30 | 62.58 | 79.36 | 47.57 | 69.71 | 90.27 | 51.59 | 0.41 | 0.44 | 0.37 |
| South Africa | 92086.18 | 101782.07 | 83170.08 | 118.50 | 135.50 | 98.80 | 180.12 | 198.20 | 164.00 | 1.80 | 2.04 | 1.57 |
| South Sudan | 4156.65 | 5786.99 | 3067.88 | 59.41 | 78.03 | 46.31 | 82.78 | 111.71 | 62.47 | 1.00 | 1.22 | 0.77 |
| Spain | 319752.73 | 342836.69 | 294488.56 | 398.97 | 438.49 | 364.14 | 379.38 | 407.31 | 350.49 | -0.09 | 0.02 | -0.20 |
| Sri Lanka | 41353.70 | 53682.56 | 29331.42 | 77.87 | 89.67 | 67.55 | 152.52 | 197.59 | 108.17 | 2.64 | 2.79 | 2.49 |
| Sudan | 24561.89 | 37660.00 | 15494.73 | 52.31 | 69.46 | 38.87 | 93.23 | 138.40 | 62.39 | 2.11 | 2.47 | 1.75 |
| Suriname | 1029.75 | 1287.01 | 817.38 | 121.87 | 139.78 | 105.82 | 158.20 | 197.08 | 125.68 | 1.14 | 1.27 | 1.01 |
| Sweden | 72481.84 | 80242.34 | 64042.63 | 447.85 | 493.23 | 410.04 | 381.77 | 421.94 | 337.25 | -0.17 | 0.10 | -0.45 |
| Switzerland | 63738.79 | 68321.75 | 59054.91 | 445.14 | 488.66 | 405.63 | 385.29 | 410.87 | 358.08 | -0.31 | 0.01 | -0.64 |
| Syrian Arab Republic | 32625.95 | 42055.24 | 25313.39 | 111.28 | 135.18 | 90.41 | 221.12 | 282.17 | 173.61 | 2.38 | 2.52 | 2.24 |
| Taiwan (Province of China) | 113702.63 | 122349.92 | 104953.65 | 119.04 | 130.51 | 109.31 | 292.70 | 314.82 | 269.23 | 3.15 | 3.48 | 2.83 |
| Tajikistan | 6603.16 | 9378.81 | 4462.06 | 112.27 | 132.17 | 94.22 | 88.86 | 122.67 | 63.17 | -0.95 | -0.82 | -1.08 |
| Thailand | 234126.78 | 290881.09 | 187856.23 | 96.21 | 112.80 | 81.10 | 229.44 | 285.29 | 183.05 | 3.05 | 3.25 | 2.85 |
| Timor-Leste | 758.15 | 982.58 | 557.23 | 49.37 | 67.63 | 36.79 | 81.83 | 105.90 | 60.39 | 1.96 | 2.23 | 1.69 |
| Togo | 5197.44 | 6918.71 | 3763.87 | 67.98 | 82.60 | 56.24 | 106.13 | 138.54 | 79.85 | 1.44 | 1.59 | 1.28 |
| Tokelau | 3.34 | 4.28 | 2.63 | 166.93 | 220.43 | 123.03 | 234.11 | 300.31 | 184.75 | 0.95 | 1.02 | 0.88 |
| Tonga | 234.05 | 301.62 | 181.02 | 224.89 | 272.91 | 185.47 | 279.24 | 357.54 | 217.46 | 0.40 | 0.51 | 0.30 |
| Trinidad and Tobago | 5451.96 | 6832.23 | 4244.71 | 209.63 | 227.23 | 193.17 | 290.15 | 364.51 | 225.33 | 1.23 | 1.41 | 1.06 |
| Tunisia | 33568.33 | 44411.11 | 25356.16 | 122.25 | 145.07 | 104.03 | 241.74 | 319.50 | 183.32 | 2.37 | 2.42 | 2.31 |
| Turkmenistan | 6199.50 | 8008.65 | 4844.09 | 115.26 | 129.69 | 101.93 | 131.45 | 167.03 | 104.72 | 5.86 | 6.66 | 5.06 |
| Tuvalu | 19.13 | 25.29 | 14.71 | 138.24 | 184.61 | 102.91 | 174.57 | 228.85 | 135.44 | 0.82 | 1.21 | 0.44 |
| Turkey | 274996.08 | 331761.98 | 223935.24 | 74.22 | 86.53 | 61.86 | 284.20 | 341.68 | 231.87 | 0.74 | 0.78 | 0.70 |
| Uganda | 29531.27 | 38519.72 | 22184.36 | 84.97 | 108.39 | 67.17 | 159.24 | 203.21 | 123.32 | 1.76 | 1.90 | 1.62 |
| Ukraine | 151032.21 | 201313.15 | 112877.73 | 260.80 | 289.61 | 239.18 | 207.75 | 281.93 | 152.12 | -1.03 | -0.89 | -1.17 |
| United Arab Emirates | 13042.93 | 17174.83 | 9651.28 | 166.61 | 205.17 | 133.88 | 258.38 | 330.32 | 200.42 | 2.69 | 3.12 | 2.25 |
| United Kingdom | 547657.04 | 574764.98 | 517661.79 | 565.71 | 609.07 | 528.12 | 476.17 | 496.97 | 454.08 | -0.52 | -0.40 | -0.63 |
| United Republic of Tanzania | 39620.33 | 51048.26 | 30099.25 | 83.68 | 99.19 | 70.36 | 123.22 | 155.60 | 95.42 | 1.26 | 1.39 | 1.13 |
| United States of America | 2999127.34 | 3167566.55 | 2813936.87 | 695.03 | 741.45 | 653.53 | 555.99 | 584.68 | 525.23 | -0.88 | -0.79 | -0.97 |
| United States Virgin Islands | 433.02 | 583.20 | 323.86 | 291.94 | 342.95 | 248.58 | 293.32 | 400.67 | 215.61 | 0.23 | 0.36 | 0.11 |
| Uruguay | 19350.19 | 20730.55 | 17846.70 | 331.66 | 359.62 | 307.64 | 390.72 | 418.93 | 359.85 | 0.46 | 0.56 | 0.36 |
| Uzbekistan | 37283.55 | 43318.98 | 31743.45 | 120.53 | 134.20 | 107.82 | 117.31 | 135.61 | 100.94 | 0.06 | 0.26 | -0.13 |
| Vanuatu | 234.21 | 298.25 | 178.55 | 79.08 | 101.71 | 61.49 | 108.99 | 136.08 | 85.50 | 0.93 | 1.07 | 0.79 |
| Venezuela (Bolivarian Republic of) | 101444.90 | 128500.02 | 78339.68 | 152.90 | 163.62 | 142.75 | 325.01 | 410.07 | 252.02 | 2.37 | 2.52 | 2.22 |
| Viet Nam | 143002.69 | 183329.03 | 113862.20 | 70.76 | 86.62 | 58.39 | 130.00 | 164.85 | 104.96 | 2.21 | 2.31 | 2.12 |
| Yemen | 14700.21 | 20085.00 | 10608.88 | 48.22 | 59.90 | 36.71 | 79.95 | 106.73 | 59.75 | 2.08 | 2.43 | 1.73 |
| Zambia | 17207.81 | 26978.74 | 9849.28 | 77.47 | 100.72 | 59.83 | 169.37 | 251.29 | 105.96 | 2.76 | 3.19 | 2.33 |
| Zimbabwe | 14433.42 | 19373.03 | 10773.72 | 90.45 | 110.27 | 74.20 | 168.36 | 218.32 | 130.21 | 2.76 | 3.21 | 2.30 |

EAPC: estimated annual percentage change; UI: uncertainty interval; CI: confidence interval.

Table S5. The all-ages numbers and the age-standardized rates of DALYs, and its corresponding EAPC of breast cancer among 204 countries in 1990 and 2021

| Characteristics | Counts (2021) | | | Age-standardized DALYs rate (per 100,000) (95% UI) | | | | | | EAPC (95% CI)1990–2021 | | |
| --- | --- | --- | --- | --- | --- | --- | --- | --- | --- | --- | --- | --- |
|  |  |  |  | 1990 | | | 2021 | | |  |  |  |
|  | val | upper | lower | val | upper | lower | val | upper | lower | val | upper | lower |
| Afghanistan | 37533.18 | 69699.41 | 18285.33 | 163.01 | 284.37 | 81.22 | 244.68 | 424.56 | 129.61 | 1.43 | 1.51 | 1.35 |
| Albania | 6215.53 | 8277.61 | 4457.64 | 144.83 | 181.83 | 113.67 | 162.85 | 216.60 | 116.70 | 0.79 | 1.00 | 0.59 |
| Algeria | 53601.19 | 70802.28 | 40143.50 | 114.92 | 149.57 | 87.10 | 128.90 | 167.85 | 97.51 | 0.47 | 0.56 | 0.38 |
| American Samoa | 314.69 | 390.47 | 245.43 | 393.82 | 472.69 | 324.64 | 609.92 | 757.12 | 478.41 | 1.72 | 1.84 | 1.61 |
| Andorra | 387.20 | 536.72 | 267.98 | 354.60 | 512.70 | 250.23 | 262.22 | 365.29 | 179.79 | -0.57 | -0.40 | -0.74 |
| Angola | 51271.47 | 69771.13 | 34998.56 | 209.99 | 289.17 | 151.16 | 314.47 | 423.52 | 216.30 | 1.40 | 1.49 | 1.31 |
| Antigua and Barbuda | 515.18 | 556.15 | 479.08 | 420.19 | 466.36 | 378.20 | 462.49 | 498.36 | 431.37 | 0.54 | 0.73 | 0.34 |
| Argentina | 193925.43 | 209369.31 | 177486.81 | 480.95 | 505.92 | 452.93 | 361.85 | 391.22 | 331.19 | -0.88 | -0.75 | -1.01 |
| Armenia | 13337.58 | 15279.17 | 11714.61 | 515.00 | 548.82 | 480.23 | 315.28 | 360.89 | 277.25 | -1.63 | -1.30 | -1.96 |
| Australia | 92252.79 | 102679.65 | 81488.28 | 400.67 | 424.74 | 378.41 | 235.61 | 262.33 | 210.62 | -1.84 | -1.78 | -1.90 |
| Austria | 38263.98 | 42276.68 | 34050.84 | 451.99 | 480.68 | 422.42 | 239.20 | 262.79 | 216.30 | -1.95 | -1.87 | -2.02 |
| Azerbaijan | 29616.08 | 38131.18 | 21917.48 | 316.32 | 361.17 | 261.41 | 245.56 | 313.18 | 183.10 | -0.80 | -0.69 | -0.91 |
| Bahamas | 2775.07 | 3514.12 | 2221.04 | 606.57 | 664.82 | 552.69 | 626.90 | 788.56 | 503.82 | 0.30 | 0.42 | 0.17 |
| Bahrain | 3651.41 | 4731.19 | 2843.92 | 321.72 | 378.46 | 270.25 | 287.41 | 369.13 | 225.40 | -0.71 | -0.51 | -0.91 |
| Bangladesh | 235506.46 | 303371.24 | 175731.55 | 108.23 | 147.24 | 81.04 | 148.11 | 190.54 | 110.90 | 0.84 | 1.00 | 0.69 |
| Barbados | 2504.26 | 3118.80 | 2002.46 | 522.77 | 564.56 | 478.24 | 538.31 | 675.66 | 427.68 | 0.52 | 0.71 | 0.33 |
| Belarus | 38587.00 | 48625.65 | 30134.09 | 319.93 | 353.68 | 289.09 | 259.86 | 330.19 | 201.20 | -1.56 | -1.28 | -1.84 |
| Belgium | 58649.99 | 64479.06 | 51955.00 | 560.52 | 595.85 | 525.45 | 291.99 | 318.76 | 263.30 | -2.16 | -2.01 | -2.31 |
| Belize | 754.85 | 858.30 | 661.92 | 148.10 | 161.79 | 134.46 | 212.24 | 241.11 | 186.27 | 1.17 | 1.45 | 0.89 |
| Benin | 13802.90 | 18705.80 | 9800.89 | 181.90 | 217.56 | 147.17 | 209.70 | 278.83 | 153.45 | 0.38 | 0.47 | 0.29 |
| Bermuda | 357.20 | 448.93 | 296.41 | 551.09 | 614.66 | 494.09 | 303.99 | 380.31 | 250.35 | -2.35 | -2.13 | -2.57 |
| Bhutan | 917.52 | 1273.63 | 627.92 | 119.82 | 157.88 | 83.40 | 133.03 | 182.68 | 91.81 | 0.23 | 0.35 | 0.11 |
| Bolivia (Plurinational State of) | 28457.59 | 40802.74 | 18811.07 | 254.17 | 367.32 | 166.62 | 280.54 | 402.71 | 185.70 | 0.17 | 0.22 | 0.12 |
| Bosnia and Herzegovina | 14668.41 | 18274.10 | 11358.48 | 232.37 | 261.69 | 200.36 | 257.46 | 322.94 | 197.18 | 0.72 | 0.88 | 0.56 |
| Botswana | 6051.43 | 9038.32 | 4034.08 | 275.50 | 390.13 | 189.38 | 334.16 | 478.95 | 236.38 | 1.09 | 1.43 | 0.75 |
| Brazil | 741656.19 | 782333.98 | 698069.43 | 270.17 | 279.94 | 259.98 | 288.09 | 303.88 | 271.00 | -0.03 | 0.04 | -0.10 |
| Brunei Darussalam | 1433.15 | 1759.62 | 1106.42 | 274.34 | 364.58 | 202.83 | 304.96 | 373.65 | 236.84 | 0.92 | 1.16 | 0.68 |
| Bulgaria | 45217.61 | 54039.42 | 37047.09 | 361.28 | 408.42 | 322.48 | 376.36 | 450.51 | 308.55 | 0.30 | 0.42 | 0.18 |
| Burkina Faso | 40441.57 | 55503.92 | 27884.85 | 315.43 | 399.77 | 241.45 | 342.57 | 457.35 | 246.61 | 0.29 | 0.40 | 0.17 |
| Burundi | 15748.89 | 21862.49 | 11246.73 | 294.15 | 418.31 | 208.34 | 238.19 | 327.47 | 173.75 | -1.06 | -0.88 | -1.25 |
| Cabo Verde | 1092.00 | 1393.02 | 838.25 | 260.94 | 324.20 | 206.21 | 220.83 | 281.35 | 170.50 | -0.30 | -0.01 | -0.58 |
| Cambodia | 51430.41 | 69586.03 | 36711.21 | 244.73 | 371.15 | 154.77 | 354.38 | 474.95 | 253.90 | 1.16 | 1.22 | 1.10 |
| Cameroon | 47109.90 | 65922.81 | 33288.98 | 234.39 | 290.66 | 186.95 | 281.72 | 387.95 | 201.36 | 0.53 | 0.60 | 0.46 |
| Canada | 149682.43 | 165918.57 | 133578.90 | 431.00 | 459.33 | 401.03 | 241.05 | 266.90 | 217.57 | -1.98 | -1.91 | -2.05 |
| Central African Republic | 9321.62 | 13197.44 | 6266.60 | 268.20 | 361.45 | 192.77 | 304.50 | 416.92 | 213.57 | 0.33 | 0.39 | 0.27 |
| Chad | 14116.58 | 19076.09 | 9812.26 | 150.15 | 197.84 | 108.48 | 184.80 | 245.57 | 131.98 | 0.71 | 0.75 | 0.68 |
| Chile | 47591.77 | 52111.07 | 43040.72 | 274.68 | 292.79 | 257.50 | 194.62 | 213.10 | 176.43 | -0.91 | -0.81 | -1.00 |
| China | 3029404.67 | 3844035.90 | 2360641.16 | 151.48 | 184.39 | 123.45 | 146.26 | 185.51 | 113.82 | -0.36 | -0.26 | -0.45 |
| Colombia | 134544.72 | 162240.38 | 111840.74 | 231.25 | 247.52 | 216.06 | 244.68 | 295.31 | 203.56 | 0.15 | 0.38 | -0.08 |
| Comoros | 2200.33 | 2972.91 | 1635.62 | 285.21 | 387.58 | 204.04 | 379.87 | 507.24 | 283.11 | 0.65 | 0.80 | 0.50 |
| Congo | 17199.57 | 27460.59 | 10155.46 | 377.56 | 604.81 | 224.01 | 458.64 | 717.55 | 280.40 | 0.51 | 0.64 | 0.39 |
| Cook Islands | 135.49 | 175.64 | 102.05 | 553.51 | 712.56 | 428.47 | 579.78 | 753.32 | 432.74 | 0.31 | 0.50 | 0.13 |
| Costa Rica | 15215.36 | 17335.83 | 13342.27 | 199.83 | 215.05 | 184.81 | 276.95 | 315.46 | 243.04 | 1.10 | 1.25 | 0.94 |
| Croatia | 21819.97 | 25160.21 | 18665.38 | 381.56 | 426.74 | 336.72 | 279.12 | 319.01 | 234.91 | -1.00 | -0.83 | -1.18 |
| Cuba | 47806.59 | 56815.34 | 40481.96 | 296.14 | 315.48 | 275.85 | 262.42 | 310.63 | 222.17 | -0.36 | -0.26 | -0.45 |
| Cyprus | 5671.68 | 6892.63 | 4658.33 | 369.89 | 445.59 | 308.61 | 297.45 | 358.45 | 243.99 | -0.56 | -0.42 | -0.71 |
| Czechia | 45754.57 | 53808.55 | 38353.45 | 388.62 | 432.02 | 350.40 | 239.84 | 282.07 | 200.45 | -1.89 | -1.72 | -2.06 |
| Côte d'Ivoire | 48141.92 | 66487.46 | 34213.36 | 243.27 | 309.83 | 188.76 | 320.92 | 431.14 | 233.59 | 1.01 | 1.11 | 0.91 |
| Democratic People's Republic of Korea | 69130.54 | 94059.48 | 46575.17 | 183.69 | 269.72 | 121.35 | 201.05 | 273.25 | 137.29 | 0.45 | 0.52 | 0.38 |
| Democratic Republic of the Congo | 139923.92 | 192789.48 | 103412.25 | 226.15 | 306.15 | 159.86 | 292.11 | 402.56 | 215.35 | 0.86 | 1.08 | 0.64 |
| Denmark | 28197.41 | 31150.01 | 25309.16 | 631.38 | 665.65 | 598.24 | 269.29 | 297.00 | 244.05 | -2.76 | -2.65 | -2.88 |
| Djibouti | 2951.70 | 4419.93 | 1917.47 | 283.20 | 376.78 | 209.91 | 335.50 | 486.40 | 226.47 | 0.48 | 0.54 | 0.42 |
| Dominica | 369.54 | 466.34 | 279.20 | 445.50 | 511.68 | 382.82 | 451.22 | 566.26 | 341.34 | 0.11 | 0.19 | 0.03 |
| Dominican Republic | 23353.13 | 30695.28 | 17764.07 | 187.72 | 219.73 | 158.30 | 220.29 | 289.02 | 167.81 | 0.83 | 0.99 | 0.66 |
| Ecuador | 34030.25 | 43430.34 | 25920.79 | 144.53 | 153.57 | 135.07 | 200.31 | 255.25 | 152.87 | 1.20 | 1.41 | 0.98 |
| Egypt | 212977.01 | 266182.80 | 166696.73 | 148.87 | 177.67 | 128.69 | 268.94 | 332.65 | 214.09 | 2.31 | 2.66 | 1.97 |
| El Salvador | 13547.19 | 17267.28 | 10736.16 | 137.71 | 157.51 | 119.56 | 223.05 | 284.36 | 176.79 | 1.61 | 1.72 | 1.50 |
| Equatorial Guinea | 2975.00 | 4808.97 | 1702.52 | 252.84 | 361.26 | 169.99 | 415.73 | 652.07 | 246.95 | 1.69 | 1.80 | 1.59 |
| Eritrea | 15708.00 | 21822.48 | 10842.84 | 322.68 | 434.60 | 238.34 | 417.15 | 569.82 | 294.00 | 0.83 | 0.92 | 0.73 |
| Estonia | 5606.32 | 6639.23 | 4583.35 | 373.68 | 407.06 | 342.86 | 239.89 | 283.58 | 197.20 | -1.73 | -1.54 | -1.91 |
| Eswatini | 3049.56 | 4837.88 | 1706.39 | 272.75 | 348.07 | 207.33 | 442.19 | 689.62 | 254.81 | 1.81 | 2.26 | 1.37 |
| Ethiopia | 167188.43 | 206546.87 | 134337.33 | 297.71 | 437.64 | 199.00 | 292.48 | 360.26 | 238.37 | -0.27 | -0.10 | -0.44 |
| Fiji | 5107.42 | 6810.95 | 3727.08 | 507.73 | 641.80 | 402.97 | 595.24 | 786.12 | 440.44 | 0.63 | 0.77 | 0.49 |
| Finland | 23913.93 | 26695.45 | 21100.17 | 382.24 | 407.59 | 358.90 | 233.14 | 257.65 | 209.24 | -1.58 | -1.50 | -1.65 |
| France | 348170.61 | 384297.65 | 305783.88 | 448.48 | 474.64 | 424.62 | 301.62 | 332.02 | 269.35 | -1.30 | -1.17 | -1.43 |
| Gabon | 5295.14 | 7468.20 | 3545.11 | 342.87 | 460.33 | 243.86 | 418.01 | 576.46 | 285.35 | 0.45 | 0.57 | 0.33 |
| Gambia | 1896.02 | 2587.90 | 1356.32 | 91.61 | 122.03 | 67.83 | 155.22 | 208.64 | 112.36 | 1.50 | 1.72 | 1.28 |
| Georgia | 25290.20 | 29331.58 | 21672.66 | 504.47 | 559.20 | 450.91 | 467.68 | 543.74 | 400.21 | 0.03 | 0.27 | -0.22 |
| Germany | 472913.52 | 515359.75 | 424476.38 | 471.15 | 499.25 | 443.26 | 294.22 | 319.36 | 269.04 | -1.57 | -1.49 | -1.66 |
| Ghana | 71724.70 | 97129.69 | 52776.50 | 280.26 | 355.27 | 217.20 | 330.70 | 440.01 | 246.75 | 0.40 | 0.47 | 0.33 |
| Greece | 63052.29 | 68732.87 | 56959.92 | 406.34 | 429.57 | 382.63 | 318.23 | 344.85 | 292.94 | -1.03 | -0.90 | -1.16 |
| Greenland | 187.32 | 248.99 | 135.89 | 466.53 | 581.22 | 374.38 | 254.22 | 333.49 | 187.57 | -2.24 | -2.10 | -2.37 |
| Grenada | 548.86 | 629.44 | 472.76 | 452.36 | 504.53 | 405.78 | 471.93 | 537.48 | 407.87 | 0.45 | 0.73 | 0.16 |
| Guam | 473.83 | 566.47 | 397.02 | 243.60 | 283.36 | 209.22 | 244.76 | 289.91 | 204.70 | 0.54 | 0.74 | 0.34 |
| Guatemala | 18647.26 | 21800.20 | 15528.57 | 94.71 | 100.60 | 88.58 | 151.84 | 177.36 | 126.48 | 1.56 | 1.82 | 1.30 |
| Guinea | 18375.06 | 26183.74 | 12577.06 | 197.22 | 247.91 | 148.17 | 263.47 | 368.77 | 184.52 | 0.90 | 0.93 | 0.87 |
| Guinea-Bissau | 3566.31 | 4930.94 | 2474.08 | 256.00 | 374.89 | 177.03 | 338.53 | 463.19 | 233.87 | 0.93 | 0.95 | 0.91 |
| Guyana | 3039.46 | 4008.80 | 2251.17 | 328.90 | 377.53 | 286.57 | 422.24 | 554.32 | 314.83 | 1.22 | 1.49 | 0.96 |
| Haiti | 38514.63 | 60450.49 | 23627.39 | 356.06 | 551.80 | 216.13 | 411.00 | 638.03 | 254.25 | 0.58 | 0.66 | 0.50 |
| Honduras | 15618.80 | 21647.03 | 10948.33 | 131.73 | 169.39 | 95.68 | 214.87 | 296.61 | 153.02 | 1.58 | 1.72 | 1.44 |
| Hungary | 53979.75 | 62982.23 | 46089.26 | 439.87 | 494.77 | 390.07 | 315.73 | 370.47 | 267.70 | -1.40 | -1.26 | -1.55 |
| Iceland | 1305.46 | 1467.56 | 1137.98 | 422.23 | 454.56 | 387.65 | 253.13 | 283.82 | 223.12 | -1.73 | -1.63 | -1.84 |
| India | 2758796.68 | 3257229.70 | 2342795.84 | 140.62 | 164.26 | 119.84 | 206.15 | 243.90 | 175.14 | 1.26 | 1.42 | 1.11 |
| Indonesia | 909231.27 | 1311906.72 | 610335.36 | 233.19 | 329.15 | 161.37 | 309.78 | 445.38 | 208.64 | 0.82 | 0.93 | 0.71 |
| Iran (Islamic Republic of) | 154475.35 | 172531.77 | 138491.76 | 111.00 | 128.11 | 95.43 | 164.51 | 183.65 | 147.59 | 1.76 | 2.06 | 1.47 |
| Iraq | 86961.19 | 117459.79 | 61387.31 | 205.85 | 271.49 | 154.15 | 275.73 | 367.24 | 195.30 | 0.89 | 0.96 | 0.82 |
| Ireland | 19244.58 | 21409.26 | 17125.37 | 525.31 | 558.85 | 496.46 | 266.34 | 296.32 | 239.09 | -2.05 | -1.95 | -2.15 |
| Israel | 30656.03 | 34010.28 | 26927.70 | 486.18 | 517.29 | 456.18 | 270.00 | 297.73 | 240.65 | -2.19 | -2.00 | -2.38 |
| Italy | 329328.57 | 357262.57 | 291495.47 | 459.78 | 477.95 | 438.51 | 275.53 | 296.32 | 251.07 | -1.73 | -1.66 | -1.79 |
| Jamaica | 13812.56 | 17683.74 | 10380.69 | 315.50 | 341.59 | 292.84 | 448.16 | 574.30 | 336.96 | 1.12 | 1.43 | 0.81 |
| Japan | 427448.54 | 464798.76 | 378949.91 | 142.51 | 148.32 | 136.16 | 180.43 | 192.08 | 166.43 | 0.73 | 0.90 | 0.56 |
| Jordan | 22572.48 | 30510.69 | 15933.13 | 230.68 | 296.62 | 176.31 | 233.81 | 315.46 | 167.04 | 0.07 | 0.44 | -0.30 |
| Kazakhstan | 44284.51 | 51755.89 | 37044.75 | 389.41 | 434.47 | 345.62 | 225.38 | 263.05 | 188.78 | -1.32 | -0.98 | -1.65 |
| Kenya | 97013.48 | 134641.19 | 66223.21 | 195.48 | 265.36 | 140.52 | 328.57 | 451.21 | 230.60 | 1.87 | 1.94 | 1.79 |
| Kiribati | 481.58 | 656.47 | 355.59 | 393.67 | 502.38 | 303.82 | 540.01 | 727.17 | 405.35 | 1.13 | 1.20 | 1.06 |
| Kuwait | 6754.68 | 7962.17 | 5665.98 | 140.82 | 155.82 | 125.48 | 141.24 | 163.50 | 117.07 | -0.27 | 0.18 | -0.73 |
| Kyrgyzstan | 11538.25 | 14030.87 | 9359.00 | 321.70 | 361.25 | 286.13 | 200.73 | 242.36 | 164.12 | -1.69 | -1.52 | -1.85 |
| Lao People's Democratic Republic | 17425.38 | 23996.24 | 12257.71 | 237.33 | 384.09 | 138.70 | 297.10 | 409.11 | 209.68 | 0.80 | 0.86 | 0.74 |
| Latvia | 9989.28 | 11847.28 | 8153.04 | 369.23 | 419.20 | 326.63 | 295.15 | 351.04 | 239.16 | -0.84 | -0.63 | -1.06 |
| Lebanon | 19770.21 | 24146.75 | 15847.13 | 299.15 | 402.44 | 214.77 | 334.77 | 409.54 | 268.30 | 0.64 | 0.86 | 0.41 |
| Lesotho | 5415.10 | 7953.64 | 3376.50 | 223.64 | 314.89 | 154.91 | 443.89 | 646.60 | 280.75 | 3.04 | 3.48 | 2.59 |
| Liberia | 7177.23 | 10687.87 | 4851.01 | 168.08 | 209.43 | 130.77 | 235.42 | 335.42 | 163.49 | 1.16 | 1.29 | 1.02 |
| Libya | 14626.72 | 20609.36 | 10657.54 | 128.66 | 166.66 | 101.45 | 209.28 | 288.56 | 155.37 | 1.69 | 1.91 | 1.47 |
| Lithuania | 13805.68 | 16141.80 | 11519.14 | 333.27 | 358.46 | 310.61 | 286.01 | 333.10 | 239.71 | -0.55 | -0.33 | -0.77 |
| Luxembourg | 2484.18 | 2761.41 | 2225.19 | 520.74 | 553.76 | 487.51 | 247.20 | 273.98 | 221.19 | -2.07 | -1.91 | -2.23 |
| Madagascar | 47835.63 | 65381.63 | 33731.51 | 269.04 | 336.34 | 213.53 | 295.88 | 393.62 | 211.19 | 0.22 | 0.42 | 0.02 |
| Malawi | 31516.22 | 41999.62 | 22450.68 | 218.95 | 270.06 | 170.92 | 325.15 | 432.30 | 235.21 | 1.29 | 1.38 | 1.19 |
| Malaysia | 131316.56 | 155133.07 | 110627.43 | 331.25 | 387.62 | 278.61 | 417.90 | 491.61 | 352.90 | 0.73 | 0.87 | 0.60 |
| Maldives | 490.50 | 638.82 | 365.70 | 133.30 | 227.31 | 70.24 | 108.53 | 138.44 | 82.39 | -0.95 | -0.64 | -1.27 |
| Mali | 28998.60 | 40529.38 | 20330.16 | 229.77 | 277.60 | 187.33 | 258.40 | 354.17 | 183.44 | 0.29 | 0.38 | 0.21 |
| Malta | 2391.84 | 2699.68 | 2119.42 | 536.96 | 581.47 | 498.57 | 304.63 | 340.61 | 271.30 | -2.07 | -1.97 | -2.18 |
| Marshall Islands | 224.63 | 356.19 | 132.65 | 331.96 | 470.36 | 233.02 | 480.25 | 744.75 | 289.81 | 1.13 | 1.25 | 1.01 |
| Mauritania | 6878.38 | 9246.28 | 5164.47 | 224.69 | 305.30 | 162.30 | 272.34 | 364.46 | 206.03 | 0.45 | 0.57 | 0.33 |
| Mauritius | 6811.57 | 7307.24 | 6143.18 | 194.31 | 209.89 | 181.20 | 381.66 | 410.67 | 343.08 | 1.60 | 1.92 | 1.28 |
| Mexico | 314959.79 | 372870.42 | 261636.40 | 190.69 | 195.99 | 184.97 | 231.70 | 273.65 | 193.05 | 0.36 | 0.45 | 0.26 |
| Micronesia (Federated States of) | 461.32 | 621.26 | 327.54 | 410.56 | 565.57 | 277.47 | 526.63 | 702.31 | 378.88 | 0.78 | 0.84 | 0.72 |
| Monaco | 412.15 | 545.06 | 309.18 | 537.30 | 709.12 | 402.21 | 557.21 | 749.52 | 408.78 | 0.20 | 0.31 | 0.10 |
| Mongolia | 3033.22 | 3717.61 | 2381.69 | 94.64 | 118.83 | 73.90 | 101.85 | 124.98 | 79.07 | -0.01 | 0.17 | -0.19 |
| Montenegro | 3627.40 | 4573.41 | 2849.77 | 380.85 | 492.02 | 295.57 | 397.28 | 498.36 | 311.56 | 0.36 | 0.55 | 0.17 |
| Morocco | 63083.56 | 93314.17 | 41712.28 | 98.71 | 129.23 | 75.98 | 163.89 | 239.90 | 109.56 | 1.86 | 1.95 | 1.76 |
| Mozambique | 50787.19 | 69749.99 | 34807.15 | 230.08 | 281.94 | 189.52 | 347.37 | 464.13 | 244.15 | 1.64 | 1.77 | 1.50 |
| Myanmar | 186705.97 | 251573.72 | 139264.57 | 321.03 | 455.34 | 218.60 | 336.25 | 451.79 | 252.92 | -0.05 | 0.07 | -0.17 |
| Namibia | 8937.70 | 12877.12 | 5679.48 | 302.83 | 373.22 | 250.18 | 535.92 | 752.21 | 346.90 | 2.16 | 2.30 | 2.02 |
| Nauru | 47.94 | 75.32 | 28.14 | 452.00 | 700.86 | 270.52 | 640.76 | 994.52 | 381.85 | 1.17 | 1.26 | 1.08 |
| Nepal | 40334.20 | 55812.78 | 28781.00 | 122.77 | 160.37 | 90.94 | 154.01 | 212.41 | 110.43 | 0.79 | 1.06 | 0.52 |
| Netherlands | 89475.81 | 98665.57 | 80925.65 | 532.45 | 566.83 | 499.53 | 299.25 | 327.24 | 274.57 | -2.05 | -1.92 | -2.18 |
| New Zealand | 21158.08 | 23163.70 | 19083.11 | 535.27 | 574.92 | 500.56 | 290.54 | 317.34 | 264.96 | -1.88 | -1.81 | -1.95 |
| Nicaragua | 8541.25 | 10748.94 | 6615.71 | 106.63 | 123.76 | 90.94 | 153.18 | 191.96 | 118.96 | 1.39 | 1.57 | 1.20 |
| Niger | 15275.62 | 21376.47 | 10383.13 | 129.52 | 174.02 | 97.33 | 147.90 | 205.06 | 101.78 | 0.35 | 0.44 | 0.26 |
| Nigeria | 522743.35 | 762662.03 | 346862.62 | 261.20 | 342.57 | 195.47 | 452.99 | 647.29 | 310.10 | 1.98 | 2.20 | 1.76 |
| Niue | 10.81 | 14.27 | 8.05 | 433.81 | 564.69 | 326.52 | 534.47 | 705.76 | 402.21 | 0.38 | 0.47 | 0.29 |
| North Macedonia | 11548.37 | 14624.44 | 8926.81 | 386.08 | 463.17 | 316.28 | 361.43 | 459.11 | 279.07 | -0.26 | -0.06 | -0.45 |
| Northern Mariana Islands | 234.46 | 275.46 | 191.49 | 338.01 | 446.11 | 249.52 | 404.33 | 470.23 | 330.50 | 0.52 | 0.66 | 0.39 |
| Norway | 17177.59 | 18587.32 | 15544.85 | 372.62 | 390.11 | 355.64 | 194.65 | 210.02 | 178.37 | -2.34 | -2.16 | -2.52 |
| Oman | 1753.04 | 2319.70 | 1318.59 | 56.16 | 75.66 | 40.95 | 61.61 | 78.21 | 47.30 | 0.56 | 0.94 | 0.17 |
| Pakistan | 745586.17 | 999586.60 | 529953.34 | 297.68 | 368.71 | 235.91 | 457.47 | 608.28 | 328.88 | 0.99 | 1.21 | 0.77 |
| Palau | 129.88 | 169.75 | 98.26 | 615.33 | 802.38 | 470.14 | 564.73 | 730.35 | 433.81 | -0.19 | -0.08 | -0.29 |
| Palestine | 10603.74 | 13013.95 | 8604.39 | 304.68 | 423.93 | 217.73 | 331.61 | 405.54 | 267.80 | 0.46 | 0.58 | 0.35 |
| Panama | 10698.41 | 12982.85 | 8396.36 | 179.36 | 193.10 | 166.09 | 242.02 | 293.73 | 190.07 | 1.14 | 1.25 | 1.03 |
| Papua New Guinea | 23480.68 | 32385.55 | 16719.95 | 264.65 | 375.67 | 181.93 | 305.24 | 416.39 | 221.00 | 0.36 | 0.47 | 0.25 |
| Paraguay | 17597.57 | 23395.96 | 13135.57 | 199.10 | 240.76 | 160.04 | 278.11 | 370.58 | 208.54 | 1.08 | 1.27 | 0.89 |
| Peru | 67060.98 | 89411.10 | 48574.18 | 201.81 | 240.04 | 165.94 | 188.98 | 250.81 | 137.17 | -0.64 | -0.43 | -0.85 |
| Philippines | 393980.87 | 495793.45 | 306468.66 | 305.36 | 337.28 | 271.13 | 403.67 | 504.63 | 316.37 | 0.92 | 0.99 | 0.86 |
| Poland | 199630.98 | 222995.64 | 175028.37 | 346.57 | 358.61 | 334.72 | 307.94 | 344.24 | 270.15 | -0.57 | -0.46 | -0.68 |
| Portugal | 49836.58 | 54124.80 | 44452.74 | 421.43 | 449.61 | 396.31 | 255.84 | 278.33 | 231.92 | -1.70 | -1.61 | -1.80 |
| Puerto Rico | 14791.98 | 17404.99 | 12303.21 | 319.63 | 340.37 | 296.68 | 264.40 | 312.56 | 218.43 | -0.71 | -0.59 | -0.84 |
| Qatar | 3397.63 | 4710.65 | 2400.32 | 232.06 | 282.59 | 185.41 | 216.91 | 290.74 | 160.29 | -0.23 | -0.02 | -0.43 |
| Republic of Korea | 98164.71 | 116771.31 | 79587.24 | 103.64 | 122.63 | 91.74 | 118.46 | 140.39 | 96.40 | 0.51 | 0.60 | 0.43 |
| Republic of Moldova | 16991.69 | 20252.32 | 14388.19 | 390.78 | 434.42 | 347.05 | 298.84 | 355.97 | 252.66 | -0.60 | -0.38 | -0.81 |
| Romania | 104227.50 | 117872.91 | 90649.58 | 309.89 | 332.80 | 289.77 | 320.88 | 361.89 | 279.31 | -0.03 | 0.09 | -0.14 |
| Russian Federation | 691770.59 | 765873.39 | 615179.66 | 310.72 | 319.47 | 301.97 | 309.59 | 342.83 | 274.25 | -0.45 | -0.18 | -0.72 |
| Rwanda | 30214.01 | 41758.84 | 20706.89 | 405.86 | 575.00 | 283.10 | 381.38 | 522.62 | 267.13 | -0.69 | -0.45 | -0.92 |
| Saint Kitts and Nevis | 311.48 | 377.26 | 253.19 | 681.20 | 747.99 | 621.32 | 420.46 | 506.04 | 343.26 | -1.14 | -0.90 | -1.37 |
| Saint Lucia | 861.69 | 1049.84 | 713.79 | 488.74 | 529.16 | 450.90 | 362.31 | 441.63 | 300.29 | -1.03 | -0.76 | -1.31 |
| Saint Vincent and the Grenadines | 634.37 | 732.40 | 547.04 | 481.50 | 527.42 | 431.57 | 456.80 | 527.82 | 393.85 | -0.20 | 0.01 | -0.42 |
| Samoa | 520.60 | 698.27 | 377.18 | 265.19 | 342.01 | 198.13 | 324.94 | 432.85 | 235.96 | 0.64 | 0.71 | 0.57 |
| San Marino | 106.64 | 156.46 | 64.39 | 302.03 | 380.55 | 238.54 | 176.48 | 262.59 | 104.27 | -0.95 | -0.69 | -1.22 |
| Sao Tome and Principe | 377.20 | 500.04 | 274.30 | 174.41 | 213.79 | 140.71 | 263.46 | 344.97 | 197.39 | 1.24 | 1.38 | 1.11 |
| Saudi Arabia | 53438.65 | 74891.22 | 37359.08 | 92.38 | 124.89 | 67.36 | 139.83 | 189.19 | 103.40 | 1.38 | 1.66 | 1.09 |
| Senegal | 24364.58 | 33232.57 | 18143.64 | 180.02 | 225.44 | 140.83 | 261.27 | 350.96 | 196.57 | 1.25 | 1.38 | 1.12 |
| Serbia | 58777.73 | 75051.25 | 44548.19 | 437.08 | 554.49 | 331.38 | 398.74 | 508.71 | 302.37 | -0.58 | -0.42 | -0.75 |
| Seychelles | 429.09 | 500.88 | 362.88 | 269.54 | 311.33 | 230.12 | 338.27 | 396.28 | 287.11 | 0.74 | 1.05 | 0.44 |
| Sierra Leone | 11042.52 | 15009.81 | 7593.09 | 152.71 | 205.84 | 106.90 | 225.99 | 303.11 | 160.33 | 1.51 | 1.61 | 1.42 |
| Singapore | 15128.92 | 16531.60 | 13912.83 | 258.08 | 275.19 | 241.63 | 174.43 | 190.41 | 160.11 | -1.04 | -0.86 | -1.21 |
| Slovakia | 26473.36 | 33172.68 | 20440.38 | 356.10 | 413.41 | 310.69 | 297.89 | 369.79 | 232.66 | -0.66 | -0.57 | -0.75 |
| Slovenia | 8961.08 | 10769.04 | 7390.14 | 366.04 | 400.44 | 334.89 | 221.85 | 267.41 | 182.47 | -1.84 | -1.61 | -2.07 |
| Solomon Islands | 1679.86 | 2335.13 | 1196.73 | 203.68 | 291.90 | 125.77 | 352.28 | 480.75 | 250.13 | 1.84 | 1.96 | 1.73 |
| Somalia | 21235.05 | 30171.84 | 13965.25 | 230.82 | 316.52 | 158.24 | 244.64 | 336.37 | 166.38 | 0.16 | 0.20 | 0.11 |
| South Africa | 202893.17 | 227019.59 | 181347.97 | 297.10 | 354.39 | 240.46 | 393.55 | 436.65 | 354.78 | 1.40 | 1.63 | 1.16 |
| South Sudan | 13234.67 | 19269.28 | 9149.03 | 202.36 | 287.90 | 143.38 | 252.97 | 361.36 | 177.75 | 0.63 | 0.88 | 0.39 |
| Spain | 175311.55 | 193174.70 | 155267.93 | 397.61 | 422.77 | 373.02 | 213.27 | 234.20 | 193.22 | -2.08 | -2.00 | -2.15 |
| Sri Lanka | 53726.79 | 73543.02 | 34763.25 | 168.40 | 200.65 | 140.55 | 199.43 | 271.79 | 129.11 | 0.81 | 0.97 | 0.64 |
| Sudan | 37457.49 | 60428.56 | 21812.97 | 91.75 | 141.54 | 57.19 | 134.36 | 213.94 | 82.28 | 1.35 | 1.53 | 1.18 |
| Suriname | 1891.06 | 2416.82 | 1444.07 | 267.56 | 312.83 | 225.93 | 290.23 | 369.97 | 221.73 | 0.43 | 0.56 | 0.30 |
| Sweden | 35510.51 | 40850.31 | 29843.82 | 355.15 | 381.18 | 331.22 | 194.67 | 224.13 | 164.16 | -1.61 | -1.35 | -1.86 |
| Switzerland | 33560.62 | 37020.35 | 29193.69 | 396.80 | 422.09 | 370.36 | 207.68 | 227.78 | 184.83 | -2.10 | -1.89 | -2.30 |
| Syrian Arab Republic | 27922.92 | 37752.85 | 20234.49 | 147.06 | 186.00 | 110.11 | 185.74 | 249.18 | 136.84 | 0.59 | 0.74 | 0.43 |
| Taiwan (Province of China) | 89175.59 | 96635.72 | 81447.71 | 160.17 | 169.46 | 150.60 | 232.79 | 251.59 | 212.88 | 1.23 | 1.44 | 1.02 |
| Tajikistan | 14005.28 | 21176.89 | 8450.90 | 248.50 | 300.48 | 199.24 | 180.03 | 265.73 | 113.24 | -1.17 | -1.05 | -1.28 |
| Thailand | 287075.62 | 366179.25 | 214697.32 | 194.54 | 230.91 | 158.05 | 283.69 | 363.38 | 213.67 | 1.23 | 1.46 | 1.00 |
| Timor-Leste | 1999.61 | 2727.51 | 1364.43 | 148.00 | 225.64 | 94.05 | 214.18 | 292.13 | 145.84 | 1.29 | 1.51 | 1.06 |
| Togo | 14675.60 | 20135.77 | 10193.97 | 215.46 | 271.17 | 175.22 | 289.15 | 396.49 | 206.97 | 0.95 | 1.03 | 0.86 |
| Tokelau | 7.29 | 9.70 | 5.42 | 445.91 | 645.00 | 288.19 | 513.92 | 681.33 | 383.94 | 0.32 | 0.38 | 0.26 |
| Tonga | 497.28 | 662.56 | 358.91 | 539.30 | 681.91 | 429.77 | 587.99 | 782.05 | 427.54 | 0.14 | 0.21 | 0.07 |
| Trinidad and Tobago | 7703.85 | 9889.18 | 5851.20 | 422.05 | 457.25 | 390.56 | 414.51 | 534.53 | 315.01 | -0.08 | 0.10 | -0.25 |
| Tunisia | 26088.50 | 35813.57 | 18416.84 | 150.19 | 181.51 | 123.09 | 186.90 | 255.47 | 132.40 | 0.60 | 0.69 | 0.51 |
| Turkey | 207685.00 | 257735.35 | 163346.36 | 96.20 | 117.79 | 77.16 | 214.12 | 265.30 | 168.27 | 3.81 | 4.54 | 3.09 |
| Turkmenistan | 11018.85 | 14845.17 | 8171.68 | 248.66 | 282.31 | 217.60 | 227.23 | 304.57 | 169.10 | 0.21 | 0.65 | -0.23 |
| Tuvalu | 48.83 | 67.36 | 34.96 | 400.72 | 584.65 | 262.37 | 444.59 | 611.15 | 320.23 | 0.28 | 0.35 | 0.20 |
| Uganda | 86321.45 | 116772.60 | 61475.23 | 286.27 | 385.33 | 207.53 | 448.64 | 600.76 | 330.13 | 0.87 | 1.16 | 0.57 |
| Ukraine | 213682.10 | 315056.61 | 136483.51 | 445.87 | 477.92 | 415.39 | 302.46 | 453.01 | 190.23 | -1.79 | -1.55 | -2.03 |
| United Arab Emirates | 13218.88 | 17680.37 | 9609.54 | 235.52 | 304.55 | 178.68 | 220.40 | 288.58 | 161.36 | 1.16 | 1.66 | 0.67 |
| United Kingdom | 319901.42 | 338483.62 | 295864.30 | 577.81 | 594.41 | 559.41 | 286.31 | 301.53 | 269.20 | -2.38 | -2.31 | -2.44 |
| United Republic of Tanzania | 113525.10 | 151629.26 | 82850.04 | 278.14 | 338.67 | 223.99 | 345.07 | 459.68 | 255.00 | 0.65 | 0.72 | 0.58 |
| United States of America | 1423645.90 | 1521530.05 | 1324855.59 | 485.06 | 507.01 | 462.94 | 277.36 | 294.85 | 260.13 | -1.93 | -1.86 | -2.00 |
| United States Virgin Islands | 489.99 | 692.19 | 340.10 | 480.64 | 575.58 | 403.91 | 350.65 | 494.91 | 243.36 | -0.68 | -0.55 | -0.82 |
| Uruguay | 22140.88 | 23979.21 | 20096.25 | 579.11 | 610.37 | 547.36 | 456.35 | 492.29 | 417.54 | -0.89 | -0.81 | -0.97 |
| Uzbekistan | 67134.87 | 80476.34 | 55522.23 | 243.63 | 268.17 | 218.56 | 204.96 | 246.01 | 170.45 | -0.48 | -0.28 | -0.68 |
| Vanuatu | 726.72 | 961.57 | 518.87 | 207.51 | 294.32 | 142.49 | 329.76 | 429.69 | 240.14 | 1.29 | 1.41 | 1.17 |
| Venezuela (Bolivarian Republic of) | 104482.43 | 134683.78 | 79091.74 | 235.83 | 250.24 | 222.18 | 335.75 | 432.71 | 254.60 | 0.96 | 1.13 | 0.80 |
| Viet Nam | 212025.60 | 286747.91 | 159236.53 | 157.03 | 205.17 | 120.26 | 191.05 | 255.17 | 143.96 | 0.69 | 0.73 | 0.65 |
| Yemen | 25236.63 | 36674.70 | 16954.79 | 76.96 | 108.29 | 48.15 | 128.86 | 184.19 | 89.71 | 1.83 | 1.96 | 1.70 |
| Zambia | 53708.55 | 86960.78 | 29184.59 | 281.02 | 387.81 | 202.78 | 513.96 | 792.11 | 301.90 | 2.11 | 2.44 | 1.79 |
| Zimbabwe | 45238.12 | 62052.96 | 32071.08 | 246.32 | 319.16 | 190.39 | 504.99 | 681.47 | 363.63 | 3.29 | 4.05 | 2.53 |

DALYs: EAPC: estimated annual percentage change; UI: uncertainty interval; CI: confidence interval.

Table S6. Age structure of global male and female breast cancer incidence in 2021

| sex | age | metric | val | upper | lower | sex | age | metric | val | upper | lower |
| --- | --- | --- | --- | --- | --- | --- | --- | --- | --- | --- | --- |
| Male | <20 years | Number | 68.83 | 98.01 | 36.27 | Female | <20 years | Number | 18237.69 | 21789.17 | 14897.66 |
| Male | <20 years | Percent | 0.00 | 0.00 | 0.00 | Female | <20 years | Percent | 0.00 | 0.00 | 0.00 |
| Male | <20 years | Rate | 0.01 | 0.01 | 0.00 | Female | <20 years | Rate | 1.43 | 1.71 | 1.17 |
| Male | 20-24 years | Number | 141.10 | 184.80 | 71.02 | Female | 20-24 years | Number | 46557.76 | 53629.12 | 40213.50 |
| Male | 20-24 years | Percent | 0.00 | 0.00 | 0.00 | Female | 20-24 years | Percent | 0.00 | 0.00 | 0.00 |
| Male | 20-24 years | Rate | 0.05 | 0.06 | 0.02 | Female | 20-24 years | Rate | 15.85 | 18.26 | 13.69 |
| Male | 25-29 years | Number | 311.42 | 401.66 | 168.81 | Female | 25-29 years | Number | 170008.16 | 189234.92 | 153410.60 |
| Male | 25-29 years | Percent | 0.00 | 0.00 | 0.00 | Female | 25-29 years | Percent | 0.00 | 0.00 | 0.00 |
| Male | 25-29 years | Rate | 0.10 | 0.14 | 0.06 | Female | 25-29 years | Rate | 58.42 | 65.03 | 52.72 |
| Male | 30-34 years | Number | 656.11 | 834.19 | 341.75 | Female | 30-34 years | Number | 443259.92 | 486415.36 | 405972.39 |
| Male | 30-34 years | Percent | 0.00 | 0.00 | 0.00 | Female | 30-34 years | Percent | 0.00 | 0.00 | 0.00 |
| Male | 30-34 years | Rate | 0.21 | 0.27 | 0.11 | Female | 30-34 years | Rate | 148.28 | 162.72 | 135.81 |
| Male | 35-39 years | Number | 900.57 | 1162.83 | 554.62 | Female | 35-39 years | Number | 825173.22 | 890752.91 | 770668.17 |
| Male | 35-39 years | Percent | 0.00 | 0.00 | 0.00 | Female | 35-39 years | Percent | 0.00 | 0.00 | 0.00 |
| Male | 35-39 years | Rate | 0.32 | 0.41 | 0.20 | Female | 35-39 years | Rate | 297.04 | 320.64 | 277.42 |
| Male | 40-44 years | Number | 1563.71 | 2001.35 | 917.71 | Female | 40-44 years | Number | 1380356.89 | 1482509.52 | 1276427.23 |
| Male | 40-44 years | Percent | 0.00 | 0.00 | 0.00 | Female | 40-44 years | Percent | 0.01 | 0.01 | 0.01 |
| Male | 40-44 years | Rate | 0.62 | 0.79 | 0.36 | Female | 40-44 years | Rate | 556.39 | 597.57 | 514.50 |
| Male | 45-49 years | Number | 2823.26 | 3727.00 | 1603.27 | Female | 45-49 years | Number | 1921275.03 | 2061969.68 | 1802730.72 |
| Male | 45-49 years | Percent | 0.00 | 0.00 | 0.00 | Female | 45-49 years | Percent | 0.01 | 0.01 | 0.01 |
| Male | 45-49 years | Rate | 1.19 | 1.57 | 0.67 | Female | 45-49 years | Rate | 815.33 | 875.04 | 765.03 |
| Male | 50-54 years | Number | 3955.43 | 5240.55 | 2279.85 | Female | 50-54 years | Number | 2307333.28 | 2502973.69 | 2153706.45 |
| Male | 50-54 years | Percent | 0.00 | 0.00 | 0.00 | Female | 50-54 years | Percent | 0.01 | 0.01 | 0.01 |
| Male | 50-54 years | Rate | 1.78 | 2.36 | 1.03 | Female | 50-54 years | Rate | 1034.95 | 1122.70 | 966.04 |
| Male | 55-59 years | Number | 4764.25 | 6061.42 | 2773.67 | Female | 55-59 years | Number | 2538054.34 | 2722501.06 | 2366381.98 |
| Male | 55-59 years | Percent | 0.00 | 0.00 | 0.00 | Female | 55-59 years | Percent | 0.01 | 0.01 | 0.01 |
| Male | 55-59 years | Rate | 2.45 | 3.11 | 1.42 | Female | 55-59 years | Rate | 1262.69 | 1354.45 | 1177.28 |
| Male | 60-64 years | Number | 5564.53 | 6969.19 | 3550.98 | Female | 60-64 years | Number | 2516427.25 | 2670352.56 | 2362970.56 |
| Male | 60-64 years | Percent | 0.00 | 0.00 | 0.00 | Female | 60-64 years | Percent | 0.02 | 0.02 | 0.01 |
| Male | 60-64 years | Rate | 3.58 | 4.48 | 2.28 | Female | 60-64 years | Rate | 1529.64 | 1623.21 | 1436.36 |
| Male | 65-69 years | Number | 6491.68 | 8144.69 | 4059.76 | Female | 65-69 years | Number | 2518239.95 | 2675123.75 | 2357327.17 |
| Male | 65-69 years | Percent | 0.00 | 0.00 | 0.00 | Female | 65-69 years | Percent | 0.02 | 0.02 | 0.02 |
| Male | 65-69 years | Rate | 4.92 | 6.18 | 3.08 | Female | 65-69 years | Rate | 1748.67 | 1857.61 | 1636.93 |
| Male | 70-74 years | Number | 5644.40 | 7040.29 | 3601.23 | Female | 70-74 years | Number | 2082015.12 | 2233321.67 | 1932328.78 |
| Male | 70-74 years | Percent | 0.00 | 0.00 | 0.00 | Female | 70-74 years | Percent | 0.02 | 0.02 | 0.02 |
| Male | 70-74 years | Rate | 5.86 | 7.30 | 3.74 | Female | 70-74 years | Rate | 1902.29 | 2040.54 | 1765.53 |
| Male | 75-79 years | Number | 2603.90 | 3039.12 | 1989.97 | Female | 75-79 years | Number | 1440515.21 | 1552565.42 | 1315245.78 |
| Male | 75-79 years | Percent | 0.00 | 0.00 | 0.00 | Female | 75-79 years | Percent | 0.02 | 0.02 | 0.02 |
| Male | 75-79 years | Rate | 4.36 | 5.08 | 3.33 | Female | 75-79 years | Rate | 1998.00 | 2153.42 | 1824.26 |
| Male | 80-84 years | Number | 1786.20 | 2070.11 | 1368.28 | Female | 80-84 years | Number | 1085292.22 | 1202373.82 | 942676.46 |
| Male | 80-84 years | Percent | 0.00 | 0.00 | 0.00 | Female | 80-84 years | Percent | 0.02 | 0.02 | 0.02 |
| Male | 80-84 years | Rate | 4.87 | 5.65 | 3.73 | Female | 80-84 years | Rate | 2130.90 | 2360.78 | 1850.88 |
| Male | 85-89 years | Number | 1165.98 | 1329.59 | 948.66 | Female | 85-89 years | Number | 670436.35 | 756277.04 | 579931.33 |
| Male | 85-89 years | Percent | 0.00 | 0.00 | 0.00 | Female | 85-89 years | Percent | 0.02 | 0.03 | 0.02 |
| Male | 85-89 years | Rate | 6.76 | 7.71 | 5.50 | Female | 85-89 years | Rate | 2354.96 | 2656.48 | 2037.05 |
| Male | 90-94 years | Number | 314.40 | 359.52 | 256.69 | Female | 90-94 years | Number | 264325.62 | 308704.83 | 225786.07 |
| Male | 90-94 years | Percent | 0.00 | 0.00 | 0.00 | Female | 90-94 years | Percent | 0.02 | 0.03 | 0.02 |
| Male | 90-94 years | Rate | 5.39 | 6.17 | 4.40 | Female | 90-94 years | Rate | 2191.60 | 2559.56 | 1872.05 |
| Male | 95+ years | Number | 71.53 | 83.08 | 56.90 | Female | 95+ years | Number | 95671.28 | 115031.76 | 80242.88 |
| Male | 95+ years | Percent | 0.00 | 0.00 | 0.00 | Female | 95+ years | Percent | 0.02 | 0.03 | 0.02 |
| Male | 95+ years | Rate | 4.73 | 5.49 | 3.76 | Female | 95+ years | Rate | 2429.27 | 2920.87 | 2037.52 |
| Male | Age-standardized | Percent | 0.00 | 0.00 | 0.00 | Female | Age-standardized | Percent | 0.00 | 0.00 | 0.00 |
| Male | Age-standardized | Rate | 0.94 | 1.15 | 0.60 | Female | Age-standardized | Rate | 450.64 | 475.96 | 427.02 |
| Male | All ages | Number | 38827.30 | 47845.89 | 24650.48 | Female | All ages | Number | 20323179.27 | 21451411.54 | 19248043.55 |
| Male | All ages | Percent | 0.00 | 0.00 | 0.00 | Female | All ages | Percent | 0.01 | 0.01 | 0.01 |
| Male | All ages | Rate | 0.98 | 1.21 | 0.62 | Female | All ages | Rate | 516.87 | 545.57 | 489.53 |

Table S7. Age structure of global male and female breast cancer mortality in 2021

| sex | age | metric | val | upper | lower | sex | age | metric | val | upper | lower |
| --- | --- | --- | --- | --- | --- | --- | --- | --- | --- | --- | --- |
| Male | <20 years | Number | 17.36 | 26.34 | 10.38 | Female | <20 years | Number | 621.79 | 782.94 | 484.68 |
| Male | <20 years | Percent | 0.00 | 0.00 | 0.00 | Female | <20 years | Percent | 0.00 | 0.00 | 0.00 |
| Male | <20 years | Rate | 0.00 | 0.00 | 0.00 | Female | <20 years | Rate | 0.05 | 0.06 | 0.04 |
| Male | 20-24 years | Number | 32.72 | 49.18 | 19.39 | Female | 20-24 years | Number | 1562.61 | 1899.60 | 1282.45 |
| Male | 20-24 years | Percent | 0.00 | 0.00 | 0.00 | Female | 20-24 years | Percent | 0.01 | 0.01 | 0.00 |
| Male | 20-24 years | Rate | 0.01 | 0.02 | 0.01 | Female | 20-24 years | Rate | 0.53 | 0.65 | 0.44 |
| Male | 25-29 years | Number | 56.70 | 78.33 | 34.53 | Female | 25-29 years | Number | 4288.67 | 4912.19 | 3733.89 |
| Male | 25-29 years | Percent | 0.00 | 0.00 | 0.00 | Female | 25-29 years | Percent | 0.01 | 0.02 | 0.01 |
| Male | 25-29 years | Rate | 0.02 | 0.03 | 0.01 | Female | 25-29 years | Rate | 1.47 | 1.69 | 1.28 |
| Male | 30-34 years | Number | 145.64 | 196.98 | 89.60 | Female | 30-34 years | Number | 11871.53 | 13159.71 | 10729.00 |
| Male | 30-34 years | Percent | 0.00 | 0.00 | 0.00 | Female | 30-34 years | Percent | 0.03 | 0.03 | 0.03 |
| Male | 30-34 years | Rate | 0.05 | 0.06 | 0.03 | Female | 30-34 years | Rate | 3.97 | 4.40 | 3.59 |
| Male | 35-39 years | Number | 255.58 | 361.12 | 167.47 | Female | 35-39 years | Number | 23202.83 | 25098.97 | 21499.06 |
| Male | 35-39 years | Percent | 0.00 | 0.00 | 0.00 | Female | 35-39 years | Percent | 0.05 | 0.05 | 0.04 |
| Male | 35-39 years | Rate | 0.09 | 0.13 | 0.06 | Female | 35-39 years | Rate | 8.35 | 9.03 | 7.74 |
| Male | 40-44 years | Number | 417.31 | 566.99 | 275.03 | Female | 40-44 years | Number | 36340.28 | 39161.14 | 33563.14 |
| Male | 40-44 years | Percent | 0.00 | 0.00 | 0.00 | Female | 40-44 years | Percent | 0.06 | 0.06 | 0.05 |
| Male | 40-44 years | Rate | 0.17 | 0.22 | 0.11 | Female | 40-44 years | Rate | 14.65 | 15.79 | 13.53 |
| Male | 45-49 years | Number | 693.08 | 915.31 | 446.93 | Female | 45-49 years | Number | 51517.88 | 55393.86 | 47903.00 |
| Male | 45-49 years | Percent | 0.00 | 0.00 | 0.00 | Female | 45-49 years | Percent | 0.06 | 0.07 | 0.06 |
| Male | 45-49 years | Rate | 0.29 | 0.38 | 0.19 | Female | 45-49 years | Rate | 21.86 | 23.51 | 20.33 |
| Male | 50-54 years | Rate | 0.49 | 0.64 | 0.31 | Female | 50-54 years | Number | 30.78 | 33.29 | 28.38 |
| Male | 50-54 years | Number | 1081.42 | 1419.31 | 677.87 | Female | 50-54 years | Percent | 68617.46 | 74224.88 | 63262.08 |
| Male | 50-54 years | Percent | 0.00 | 0.00 | 0.00 | Female | 50-54 years | Rate | 0.06 | 0.07 | 0.06 |
| Male | 55-59 years | Number | 1490.57 | 1874.14 | 916.56 | Female | 55-59 years | Number | 79159.17 | 86645.18 | 72299.67 |
| Male | 55-59 years | Percent | 0.00 | 0.00 | 0.00 | Female | 55-59 years | Percent | 0.05 | 0.05 | 0.05 |
| Male | 55-59 years | Rate | 0.77 | 0.96 | 0.47 | Female | 55-59 years | Rate | 39.38 | 43.11 | 35.97 |
| Male | 60-64 years | Number | 1741.20 | 2152.62 | 1166.42 | Female | 60-64 years | Number | 71376.87 | 76271.71 | 66772.33 |
| Male | 60-64 years | Percent | 0.00 | 0.00 | 0.00 | Female | 60-64 years | Percent | 0.04 | 0.04 | 0.03 |
| Male | 60-64 years | Rate | 1.12 | 1.38 | 0.75 | Female | 60-64 years | Rate | 43.39 | 46.36 | 40.59 |
| Male | 65-69 years | Number | 2018.16 | 2489.45 | 1352.51 | Female | 65-69 years | Number | 72320.64 | 77349.69 | 67409.99 |
| Male | 65-69 years | Percent | 0.00 | 0.00 | 0.00 | Female | 65-69 years | Percent | 0.03 | 0.03 | 0.03 |
| Male | 65-69 years | Rate | 1.53 | 1.89 | 1.03 | Female | 65-69 years | Rate | 50.22 | 53.71 | 46.81 |
| Male | 70-74 years | Number | 1987.53 | 2416.15 | 1322.83 | Female | 70-74 years | Number | 66897.86 | 71142.16 | 61584.97 |
| Male | 70-74 years | Percent | 0.00 | 0.00 | 0.00 | Female | 70-74 years | Percent | 0.02 | 0.02 | 0.02 |
| Male | 70-74 years | Rate | 2.06 | 2.51 | 1.37 | Female | 70-74 years | Rate | 61.12 | 65.00 | 56.27 |
| Male | 75-79 years | Number | 1219.11 | 1516.66 | 911.68 | Female | 75-79 years | Number | 52540.10 | 56242.16 | 47026.99 |
| Male | 75-79 years | Percent | 0.00 | 0.00 | 0.00 | Female | 75-79 years | Percent | 0.02 | 0.02 | 0.01 |
| Male | 75-79 years | Rate | 2.04 | 2.54 | 1.52 | Female | 75-79 years | Rate | 72.87 | 78.01 | 65.23 |
| Male | 80-84 years | Number | 992.56 | 1183.49 | 735.79 | Female | 80-84 years | Number | 49377.54 | 54040.76 | 41184.92 |
| Male | 80-84 years | Percent | 0.00 | 0.00 | 0.00 | Female | 80-84 years | Percent | 0.01 | 0.01 | 0.01 |
| Male | 80-84 years | Rate | 2.71 | 3.23 | 2.01 | Female | 80-84 years | Rate | 96.95 | 106.11 | 80.86 |
| Male | 85-89 years | Number | 708.79 | 822.35 | 553.55 | Female | 85-89 years | Number | 36911.33 | 41201.47 | 29100.31 |
| Male | 85-89 years | Percent | 0.00 | 0.00 | 0.00 | Female | 85-89 years | Percent | 0.01 | 0.01 | 0.01 |
| Male | 85-89 years | Rate | 4.11 | 4.77 | 3.21 | Female | 85-89 years | Rate | 129.65 | 144.72 | 102.22 |
| Male | 90-94 years | Number | 317.22 | 364.42 | 252.96 | Female | 90-94 years | Number | 23589.40 | 26663.78 | 17763.61 |
| Male | 90-94 years | Percent | 0.00 | 0.00 | 0.00 | Female | 90-94 years | Percent | 0.01 | 0.01 | 0.01 |
| Male | 90-94 years | Rate | 5.44 | 6.25 | 4.34 | Female | 90-94 years | Rate | 195.59 | 221.08 | 147.28 |
| Male | 95+ years | Number | 99.16 | 115.34 | 78.56 | Female | 95+ years | Number | 10729.33 | 12296.41 | 7650.73 |
| Male | 95+ years | Percent | 0.00 | 0.00 | 0.00 | Female | 95+ years | Percent | 0.01 | 0.01 | 0.01 |
| Male | 95+ years | Rate | 6.56 | 7.63 | 5.20 | Female | 95+ years | Rate | 272.44 | 312.23 | 194.27 |
| Male | Age-standardized | Rate | 0.34 | 0.41 | 0.23 | Female | Age-standardized | Rate | 14.55 | 15.56 | 13.45 |
| Male | All ages | Number | 13274.11 | 16240.06 | 9074.30 | Female | All ages | Number | 660925.30 | 707181.86 | 609171.34 |
| Male | All ages | Percent | 0.00 | 0.00 | 0.00 | Female | All ages | Percent | 0.02 | 0.02 | 0.02 |
| Male | All ages | Rate | 0.34 | 0.41 | 0.23 | Female | All ages | Rate | 16.81 | 17.99 | 15.49 |
| sex | age | metric | val | upper | lower | sex | age | metric | val | upper | lower |

Table S8. Age structure of global male and female breast cancer prevalence in 2021

| sex | age | metric | val | upper | lower | sex | age | metric | val | upper | lower |
| --- | --- | --- | --- | --- | --- | --- | --- | --- | --- | --- | --- |
| Male | <20 years | Number | 17.36 | 26.34 | 10.38 | Female | <20 years | Number | 621.79 | 782.94 | 484.68 |
| Male | <20 years | Percent | 0.00 | 0.00 | 0.00 | Female | <20 years | Percent | 0.00 | 0.00 | 0.00 |
| Male | <20 years | Rate | 0.00 | 0.00 | 0.00 | Female | <20 years | Rate | 0.05 | 0.06 | 0.04 |
| Male | 20-24 years | Number | 32.72 | 49.18 | 19.39 | Female | 20-24 years | Number | 1562.61 | 1899.60 | 1282.45 |
| Male | 20-24 years | Percent | 0.00 | 0.00 | 0.00 | Female | 20-24 years | Percent | 0.01 | 0.01 | 0.00 |
| Male | 20-24 years | Rate | 0.01 | 0.02 | 0.01 | Female | 20-24 years | Rate | 0.53 | 0.65 | 0.44 |
| Male | 25-29 years | Number | 56.70 | 78.33 | 34.53 | Female | 25-29 years | Number | 4288.67 | 4912.19 | 3733.89 |
| Male | 25-29 years | Percent | 0.00 | 0.00 | 0.00 | Female | 25-29 years | Percent | 0.01 | 0.02 | 0.01 |
| Male | 25-29 years | Rate | 0.02 | 0.03 | 0.01 | Female | 25-29 years | Rate | 1.47 | 1.69 | 1.28 |
| Male | 30-34 years | Number | 145.64 | 196.98 | 89.60 | Female | 30-34 years | Number | 11871.53 | 13159.71 | 10729.00 |
| Male | 30-34 years | Percent | 0.00 | 0.00 | 0.00 | Female | 30-34 years | Percent | 0.03 | 0.03 | 0.03 |
| Male | 30-34 years | Rate | 0.05 | 0.06 | 0.03 | Female | 30-34 years | Rate | 3.97 | 4.40 | 3.59 |
| Male | 35-39 years | Number | 255.58 | 361.12 | 167.47 | Female | 35-39 years | Number | 23202.83 | 25098.97 | 21499.06 |
| Male | 35-39 years | Percent | 0.00 | 0.00 | 0.00 | Female | 35-39 years | Percent | 0.05 | 0.05 | 0.04 |
| Male | 35-39 years | Rate | 0.09 | 0.13 | 0.06 | Female | 35-39 years | Rate | 8.35 | 9.03 | 7.74 |
| Male | 40-44 years | Number | 417.31 | 566.99 | 275.03 | Female | 40-44 years | Number | 36340.28 | 39161.14 | 33563.14 |
| Male | 40-44 years | Percent | 0.00 | 0.00 | 0.00 | Female | 40-44 years | Percent | 0.06 | 0.06 | 0.05 |
| Male | 40-44 years | Rate | 0.17 | 0.22 | 0.11 | Female | 40-44 years | Rate | 14.65 | 15.79 | 13.53 |
| Male | 45-49 years | Number | 693.08 | 915.31 | 446.93 | Female | 45-49 years | Number | 51517.88 | 55393.86 | 47903.00 |
| Male | 45-49 years | Percent | 0.00 | 0.00 | 0.00 | Female | 45-49 years | Percent | 0.06 | 0.07 | 0.06 |
| Male | 45-49 years | Rate | 0.29 | 0.38 | 0.19 | Female | 45-49 years | Rate | 21.86 | 23.51 | 20.33 |
| Male | 50-54 years | Rate | 0.49 | 0.64 | 0.31 | Female | 50-54 years | Number | 30.78 | 33.29 | 28.38 |
| Male | 50-54 years | Number | 1081.42 | 1419.31 | 677.87 | Female | 50-54 years | Percent | 68617.46 | 74224.88 | 63262.08 |
| Male | 50-54 years | Percent | 0.00 | 0.00 | 0.00 | Female | 50-54 years | Rate | 0.06 | 0.07 | 0.06 |
| Male | 55-59 years | Number | 1490.57 | 1874.14 | 916.56 | Female | 55-59 years | Number | 79159.17 | 86645.18 | 72299.67 |
| Male | 55-59 years | Percent | 0.00 | 0.00 | 0.00 | Female | 55-59 years | Percent | 0.05 | 0.05 | 0.05 |
| Male | 55-59 years | Rate | 0.77 | 0.96 | 0.47 | Female | 55-59 years | Rate | 39.38 | 43.11 | 35.97 |
| Male | 60-64 years | Number | 1741.20 | 2152.62 | 1166.42 | Female | 60-64 years | Number | 71376.87 | 76271.71 | 66772.33 |
| Male | 60-64 years | Percent | 0.00 | 0.00 | 0.00 | Female | 60-64 years | Percent | 0.04 | 0.04 | 0.03 |
| Male | 60-64 years | Rate | 1.12 | 1.38 | 0.75 | Female | 60-64 years | Rate | 43.39 | 46.36 | 40.59 |
| Male | 65-69 years | Number | 2018.16 | 2489.45 | 1352.51 | Female | 65-69 years | Number | 72320.64 | 77349.69 | 67409.99 |
| Male | 65-69 years | Percent | 0.00 | 0.00 | 0.00 | Female | 65-69 years | Percent | 0.03 | 0.03 | 0.03 |
| Male | 65-69 years | Rate | 1.53 | 1.89 | 1.03 | Female | 65-69 years | Rate | 50.22 | 53.71 | 46.81 |
| Male | 70-74 years | Number | 1987.53 | 2416.15 | 1322.83 | Female | 70-74 years | Number | 66897.86 | 71142.16 | 61584.97 |
| Male | 70-74 years | Percent | 0.00 | 0.00 | 0.00 | Female | 70-74 years | Percent | 0.02 | 0.02 | 0.02 |
| Male | 70-74 years | Rate | 2.06 | 2.51 | 1.37 | Female | 70-74 years | Rate | 61.12 | 65.00 | 56.27 |
| Male | 75-79 years | Number | 1219.11 | 1516.66 | 911.68 | Female | 75-79 years | Number | 52540.10 | 56242.16 | 47026.99 |
| Male | 75-79 years | Percent | 0.00 | 0.00 | 0.00 | Female | 75-79 years | Percent | 0.02 | 0.02 | 0.01 |
| Male | 75-79 years | Rate | 2.04 | 2.54 | 1.52 | Female | 75-79 years | Rate | 72.87 | 78.01 | 65.23 |
| Male | 80-84 years | Number | 992.56 | 1183.49 | 735.79 | Female | 80-84 years | Number | 49377.54 | 54040.76 | 41184.92 |
| Male | 80-84 years | Percent | 0.00 | 0.00 | 0.00 | Female | 80-84 years | Percent | 0.01 | 0.01 | 0.01 |
| Male | 80-84 years | Rate | 2.71 | 3.23 | 2.01 | Female | 80-84 years | Rate | 96.95 | 106.11 | 80.86 |
| Male | 85-89 years | Number | 708.79 | 822.35 | 553.55 | Female | 85-89 years | Number | 36911.33 | 41201.47 | 29100.31 |
| Male | 85-89 years | Percent | 0.00 | 0.00 | 0.00 | Female | 85-89 years | Percent | 0.01 | 0.01 | 0.01 |
| Male | 85-89 years | Rate | 4.11 | 4.77 | 3.21 | Female | 85-89 years | Rate | 129.65 | 144.72 | 102.22 |
| Male | 90-94 years | Number | 317.22 | 364.42 | 252.96 | Female | 90-94 years | Number | 23589.40 | 26663.78 | 17763.61 |
| Male | 90-94 years | Percent | 0.00 | 0.00 | 0.00 | Female | 90-94 years | Percent | 0.01 | 0.01 | 0.01 |
| Male | 90-94 years | Rate | 5.44 | 6.25 | 4.34 | Female | 90-94 years | Rate | 195.59 | 221.08 | 147.28 |
| Male | 95+ years | Number | 99.16 | 115.34 | 78.56 | Female | 95+ years | Number | 10729.33 | 12296.41 | 7650.73 |
| Male | 95+ years | Percent | 0.00 | 0.00 | 0.00 | Female | 95+ years | Percent | 0.01 | 0.01 | 0.01 |
| Male | 95+ years | Rate | 6.56 | 7.63 | 5.20 | Female | 95+ years | Rate | 272.44 | 312.23 | 194.27 |
| Male | Age-standardized | Rate | 0.34 | 0.41 | 0.23 | Female | Age-standardized | Rate | 14.55 | 15.56 | 13.45 |
| Male | All ages | Number | 13274.11 | 16240.06 | 9074.30 | Female | All ages | Number | 660925.30 | 707181.86 | 609171.34 |
| Male | All ages | Percent | 0.00 | 0.00 | 0.00 | Female | All ages | Percent | 0.02 | 0.02 | 0.02 |
| Male | All ages | Rate | 0.34 | 0.41 | 0.23 | Female | All ages | Rate | 16.81 | 17.99 | 15.49 |

Table S9. Age structure of global male and female breast cancer DALYs in 2021

| sex | age | metric | val | upper | lower | sex | age | metric | val | upper | lower |
| --- | --- | --- | --- | --- | --- | --- | --- | --- | --- | --- | --- |
| Male | <20 years | Number | 1310.63 | 1984.47 | 777.33 | Female | <20 years | Number | 46613.58 | 58510.00 | 36527.42 |
| Male | <20 years | Percent | 0.00 | 0.00 | 0.00 | Female | <20 years | Percent | 0.00 | 0.00 | 0.00 |
| Male | <20 years | Rate | 0.10 | 0.15 | 0.06 | Female | <20 years | Rate | 3.65 | 4.58 | 2.86 |
| Male | 20-24 years | Number | 2319.74 | 3477.42 | 1360.96 | Female | 20-24 years | Number | 109763.79 | 132497.32 | 90209.54 |
| Male | 20-24 years | Percent | 0.00 | 0.00 | 0.00 | Female | 20-24 years | Percent | 0.00 | 0.00 | 0.00 |
| Male | 20-24 years | Rate | 0.76 | 1.15 | 0.45 | Female | 20-24 years | Rate | 37.37 | 45.11 | 30.71 |
| Male | 25-29 years | Number | 3796.09 | 5233.78 | 2297.75 | Female | 25-29 years | Number | 283586.48 | 323808.69 | 247713.13 |
| Male | 25-29 years | Percent | 0.00 | 0.00 | 0.00 | Female | 25-29 years | Percent | 0.01 | 0.01 | 0.00 |
| Male | 25-29 years | Rate | 1.28 | 1.76 | 0.77 | Female | 25-29 years | Rate | 97.46 | 111.28 | 85.13 |
| Male | 30-34 years | Number | 8915.41 | 12028.88 | 5442.79 | Female | 30-34 years | Number | 722415.76 | 799881.57 | 651881.90 |
| Male | 30-34 years | Percent | 0.00 | 0.00 | 0.00 | Female | 30-34 years | Percent | 0.01 | 0.01 | 0.01 |
| Male | 30-34 years | Rate | 2.92 | 3.94 | 1.78 | Female | 30-34 years | Rate | 241.67 | 267.58 | 218.07 |
| Male | 35-39 years | Number | 14194.03 | 19996.23 | 9310.47 | Female | 35-39 years | Number | 1291789.89 | 1397030.07 | 1189540.30 |
| Male | 35-39 years | Percent | 0.00 | 0.00 | 0.00 | Female | 35-39 years | Percent | 0.02 | 0.02 | 0.02 |
| Male | 35-39 years | Rate | 5.01 | 7.06 | 3.29 | Female | 35-39 years | Rate | 465.00 | 502.89 | 428.20 |
| Male | 40-44 years | Number | 21191.18 | 28718.39 | 13872.07 | Female | 40-44 years | Number | 1846706.57 | 1991387.57 | 1702678.34 |
| Male | 40-44 years | Percent | 0.00 | 0.00 | 0.00 | Female | 40-44 years | Percent | 0.03 | 0.03 | 0.02 |
| Male | 40-44 years | Rate | 8.40 | 11.39 | 5.50 | Female | 40-44 years | Rate | 744.37 | 802.69 | 686.32 |
| Male | 45-49 years | Number | 31883.27 | 42323.82 | 20405.86 | Female | 45-49 years | Number | 2358583.84 | 2542572.45 | 2193237.10 |
| Male | 45-49 years | Percent | 0.00 | 0.00 | 0.00 | Female | 45-49 years | Percent | 0.03 | 0.04 | 0.03 |
| Male | 45-49 years | Rate | 13.40 | 17.79 | 8.58 | Female | 45-49 years | Rate | 1000.92 | 1079.00 | 930.75 |
| Male | 50-54 years | Number | 44310.95 | 58359.25 | 27749.49 | Female | 50-54 years | Number | 2790199.16 | 3013480.21 | 2572067.10 |
| Male | 50-54 years | Percent | 0.00 | 0.00 | 0.00 | Female | 50-54 years | Percent | 0.04 | 0.04 | 0.03 |
| Male | 50-54 years | Rate | 19.96 | 26.29 | 12.50 | Female | 50-54 years | Rate | 1251.53 | 1351.68 | 1153.69 |
| Male | 55-59 years | Number | 53400.95 | 67463.17 | 32642.43 | Female | 55-59 years | Number | 2833829.34 | 3124508.84 | 2593516.24 |
| Male | 55-59 years | Percent | 0.00 | 0.00 | 0.00 | Female | 55-59 years | Percent | 0.03 | 0.04 | 0.03 |
| Male | 55-59 years | Rate | 27.42 | 34.65 | 16.76 | Female | 55-59 years | Rate | 1409.84 | 1554.45 | 1290.28 |
| Male | 60-64 years | Number | 54363.37 | 67457.74 | 36272.64 | Female | 60-64 years | Number | 2233379.49 | 2389167.43 | 2089516.91 |
| Male | 60-64 years | Percent | 0.00 | 0.00 | 0.00 | Female | 60-64 years | Percent | 0.02 | 0.03 | 0.02 |
| Male | 60-64 years | Rate | 34.95 | 43.37 | 23.32 | Female | 60-64 years | Rate | 1357.59 | 1452.28 | 1270.14 |
| Male | 65-69 years | Number | 53976.60 | 66879.71 | 36154.71 | Female | 65-69 years | Number | 1927649.56 | 2063070.27 | 1792064.12 |
| Male | 65-69 years | Percent | 0.00 | 0.00 | 0.00 | Female | 65-69 years | Percent | 0.02 | 0.02 | 0.02 |
| Male | 65-69 years | Rate | 40.94 | 50.73 | 27.42 | Female | 65-69 years | Rate | 1338.56 | 1432.60 | 1244.41 |
| Male | 70-74 years | Number | 44140.02 | 53104.31 | 29508.42 | Female | 70-74 years | Number | 1474576.87 | 1574186.44 | 1359966.46 |
| Male | 70-74 years | Percent | 0.00 | 0.00 | 0.00 | Female | 70-74 years | Percent | 0.02 | 0.02 | 0.01 |
| Male | 70-74 years | Rate | 45.79 | 55.09 | 30.61 | Female | 70-74 years | Rate | 1347.29 | 1438.30 | 1242.57 |
| Male | 75-79 years | Number | 21519.00 | 26550.66 | 16182.70 | Female | 75-79 years | Number | 930806.97 | 1002380.08 | 834003.16 |
| Male | 75-79 years | Percent | 0.00 | 0.00 | 0.00 | Female | 75-79 years | Percent | 0.01 | 0.01 | 0.01 |
| Male | 75-79 years | Rate | 35.99 | 44.41 | 27.07 | Female | 75-79 years | Rate | 1291.04 | 1390.31 | 1156.77 |
| Male | 80-84 years | Number | 13941.53 | 16519.98 | 10426.87 | Female | 80-84 years | Number | 685614.46 | 752222.29 | 578370.14 |
| Male | 80-84 years | Percent | 0.00 | 0.00 | 0.00 | Female | 80-84 years | Percent | 0.01 | 0.01 | 0.01 |
| Male | 80-84 years | Rate | 38.04 | 45.07 | 28.45 | Female | 80-84 years | Rate | 1346.16 | 1476.94 | 1135.59 |
| Male | 85-89 years | Number | 7885.41 | 9125.89 | 6226.32 | Female | 85-89 years | Number | 409694.34 | 458829.04 | 326434.16 |
| Male | 85-89 years | Percent | 0.00 | 0.00 | 0.00 | Female | 85-89 years | Percent | 0.01 | 0.01 | 0.01 |
| Male | 85-89 years | Rate | 45.71 | 52.90 | 36.09 | Female | 85-89 years | Rate | 1439.08 | 1611.67 | 1146.62 |
| Male | 90-94 years | Number | 2925.76 | 3374.40 | 2337.62 | Female | 90-94 years | Number | 218645.68 | 248046.28 | 166170.49 |
| Male | 90-94 years | Percent | 0.00 | 0.00 | 0.00 | Female | 90-94 years | Percent | 0.01 | 0.01 | 0.01 |
| Male | 90-94 years | Rate | 50.20 | 57.89 | 40.11 | Female | 90-94 years | Rate | 1812.85 | 2056.62 | 1377.76 |
| Male | 95+ years | Number | 842.63 | 979.79 | 668.02 | Female | 95+ years | Number | 90945.82 | 104018.26 | 66161.68 |
| Male | 95+ years | Percent | 0.00 | 0.00 | 0.00 | Female | 95+ years | Percent | 0.01 | 0.01 | 0.01 |
| Male | 95+ years | Rate | 55.73 | 64.80 | 44.18 | Female | 95+ years | Rate | 2309.29 | 2641.22 | 1679.97 |
| Male | Age-standardized | Rate | 9.16 | 11.42 | 6.12 | Female | Age-standardized | Rate | 455.56 | 485.30 | 426.64 |
| Male | All ages | Number | 380916.57 | 476416.75 | 252900.07 | Female | All ages | Number | 20254801.61 | 21574428.57 | 18963375.54 |
| Male | All ages | Percent | 0.00 | 0.00 | 0.00 | Female | All ages | Percent | 0.02 | 0.02 | 0.01 |
| Male | All ages | Rate | 9.62 | 12.03 | 6.39 | Female | All ages | Rate | 515.13 | 548.69 | 482.29 |

table S10. Global deaths and DALYs from breast cancer risk factors in 1990 and 2021, with percentage changes

| Risk factor | Measure | Counts  (1990) | Counts  (2021) | % Change (1990 to 2021) | Age-standardized rate (per 100,000) (95% UI) | | EAPC (95% CI)  1990–2021 |
| --- | --- | --- | --- | --- | --- | --- | --- |
|  |  | (95% UI) | (95% UI) |  | 1990 | 2021 |  |
| **All risk factors** | Deaths | 98153(38827-146980) | 183039(56938-283456) | 86.48 | 2.56(1.00-3.86) | 2.14(0.67-3.31) | -0.69 (-0.73--0.65) |
|  | DALYs | 2930108(1208065 -4399195) | 5199525(1685231-8051007) | 77.45 | 70.63(28.60-106.15) | 59.76(19.42-92.46) | -0.66(-0.70--0.62) |
| **Behavioral risks** | Deaths | 76002(28881-122886) | 125707(40021-210520) | 65.40 | 1.96(0.73-3.16) | 1.47(0.47-2.47) | -1.04(-1.08--0.99) |
|  | DALYs | 2387081(935820-3818885) | 3777380(1170761-6293843) | 58.24 | 56.78(22.19-90.85) | 43.67(13.53-72.82) | -0.97(-1.02--0.93) |
| Alcohol use | Deaths | 14976(10390-20037) | 18761(12932-25749) | 25.27 | 0.38(0.26-0.51) | 0.22(0.15-0.30) | -1.82(-1.87--1.77) |
|  | DALYs | 498133(364716-644544) | 583149(412332-774078) | 17.07 | 11.68(8.50-15.26) | 6.75(4.78-8.95) | -1.88(-1.93--1.83) |
| Diet high in red meat | Deaths | 45074(-13-96485) | 81506(-26-175445) | 80.83 | 1.17(0.00-2.50) | 0.96(0.00-2.06) | -0.77(-0.82--0.73) |
|  | DALYs | 1396840(-436-3004080) | 2451719(-791-5232217) | 75.52 | 33.31(-0.01-71.68) | 28.37(-0.01-60.54) | -0.65(-0.70--0.60) |
| Low physical activity | Deaths | 8178(1663-14355) | 15964(3230-28326) | 95.21 | 0.23(0.05-0.40) | 0.19(0.04-0.34) | -0.66(-0.71--0.61) |
|  | DALYs | 219022(43912-378430) | 415254(83318-729175) | 89.59 | 5.47(1.10-9.50) | 4.81(0.97-8.45) | -0.51(-0.57--0.45) |
| Smoking | Deaths | 8967(6931-11032) | 9916(7481-12405) | 10.58 | 0.22(0.17-0.28) | 0.11(0.09-0.14) | -2.20(-2.25--2.14) |
|  | DALYs | 297373(229374-364980) | 309601(236543-388913) | 4.11 | 7.00(5.40-8.59) | 3.54(2.70-4.45) | -2.24(-2.30--2.18) |
| Secondhand Smoke | Deaths | 4949(-1227-11029) | 7518(-1811-16932) | 51.91 | 0.12(-0.03-0.27) | 0.09(-0.02-0.20) | -1.31(-1.39--1.22) |
|  | DALYs | 173838(-43356-385903) | 256510(-62469-576492) | 47,56 | 4.01(-1.00-8.89) | 2.96(-0.72-6.65) | -1.22(-1.30--1.14) |
| **Metabolic risks** | Deaths | 28660(-3746-59572) | 71960(-10281-148962) | 151.08 | 0.78(-0.10-1.62) | 0.84(-0.12-1.73) | 0.14(0.10-0.18) |
|  | DALYs | 703298(-102434-1459238) | 1783306(-281065-3602206) | 153.56 | 17.94(-2.53-37.25) | 20.18(-3.22-40.84) | 0.30(0.27-0.34) |
| High body mass index | Deaths | 18745(-604-38157) | 44707(-1478-89575) | 138.50 | 0.52(-0.02-1.06) | 0.52(-0.02-1.03) | -0.10(-0.13--0.06) |
|  | DALYs | 429066(-20024-868901) | 1041309(-40216-2029537) | 142.69 | 11.19(-0.41-22.71) | 11.63(-0.49-22.68) | 0.04(0.00  0.08) |
| High fasting plasma glucose | Deaths | 10865(-3147-25620) | 30568(-8780-72388) | 181.34 | 0.29(-0.08-0.68) | 0.36(-0.10-0.85) | 0.62(0.58  0.67) |
|  | DALYs | 296488(-86541-696925) | 819555(-240753-1938464) | 176.42 | 7.33(-2.13-17.25) | 9.43(-2.77-22.26) | 0.75(0.71-0.79) |

DALYs: disability-adjusted life years; EAPC: estimated annual percentage change; UI: uncertainty interval; CI: confidence interval.

Table S11. BAPC prediction model predicts the global age-standardized incidence and mortality of breast cancer from 2022 to 2050

| Forecast year | Age-standardized incidence rate (per 100,000) (95% CI) | | | Age-standardized mortality rate (per 100,000)  (95% CI) | | |
| --- | --- | --- | --- | --- | --- | --- |
|  | Female | Male | Both | Female | Male | Both |
| 2022 | 46.16(45.43-46.9) | 0.94(0.9-0.98) | 24.48(24.07-24.89) | 20.25(19.94-20.56) | 0.47(0.45-0.49) | 11.02(10.84-11.20) |
| 2023 | 46.12(44.9-47.33) | 0.94(0.88-1) | 24.45(23.79-25.11) | 20.15(19.64-20.66) | 0.47(0.45-0.49) | 10.96(10.68-11.25) |
| 2024 | 46.14(4.28-47.93) | 0.94(0.85-1.02) | 24.45(23.47-25.43) | 20.06(19.3-20.82) | 0.46(0.44-0.49) | 10.91(10.49-11.34) |
| 2025 | 46.14(3.58-48.63) | 0.94(0.82-1.05) | 24.45(23.10-25.80) | 19.98(18.93-21.03) | 0.46(0.43-0.5) | 10.87(10.29-11.45) |
| 2026 | 46.12(42.81-49.43) | 0.94(0.79-1.09) | 24.46(22.70-26.22) | 19.9(18.53-21.26) | 0.46(0.42-0.5) | 10.82(10.07-11.57) |
| 2027 | 46.15(41.97-50.32) | 0.94(0.75-1.12) | 24.47(22.26-26.68) | 19.82(18.11-21.54) | 0.46(0.4-0.51) | 10.78(9.84-11.72) |
| 2028 | 46.22(41.1-51.33) | 0.94(0.71-1.16) | 24.51(21.80-27.21) | 19.77(17.68-21.86) | 0.45(0.39-0.52) | 10.75(9.60-11.90) |
| 2029 | 46.32(40.2-52.43) | 0.93(0.67-1.2) | 24.56(21.33-27.79) | 19.73(17.24-22.21) | 0.45(0.38-0.53) | 10.72(9.36-12.09) |
| 2030 | 46.44(39.26-53.62) | 0.93(0.62-1.25) | 24.62(20.83-28.40) | 19.69(16.79-22.59) | 0.45(0.37-0.53) | 10.70(9.12-12.29) |
| 2031 | 46.59(38.28-54.9) | 0.93(0.58-1.29) | 24.69(20.31-29.06) | 19.66(16.31-23) | 0.45(0.35-0.54) | 10.68(8.86-12.51) |
| 2032 | 46.75(37.24-56.26) | 0.93(0.53-1.34) | 24.77(19.76-29.77) | 19.63(15.83-23.44) | 0.45(0.34-0.55) | 10.67(8.59-12.74) |
| 2033 | 46.96(36.18-57.73) | 0.93(0.48-1.39) | 24.87(19.20-30.53) | 19.63(15.34-23.92) | 0.44(0.32-0.56) | 10.66(8.32-13.00) |
| 2034 | 47.2(35.09-59.3) | 0.93(0.42-1.44) | 24.98(18.63-31.34) | 19.63(14.84-24.43) | 0.44(0.31-0.57) | 10.66(8.05-13.27) |
| 2035 | 47.46(33.96-60.96) | 0.93(0.37-1.5) | 25.11(18.03-32.19) | 19.64(14.32-24.96) | 0.44(0.29-0.59) | 10.66(7.77-13.55) |
| 2036 | 47.75(32.79-62.71) | 0.93(0.31-1.56) | 25.25(17.41-33.09) | 19.66(13.8-25.53) | 0.44(0.28-0.6) | 10.66(7.48-13.85) |
| 2037 | 48.07(31.6-64.54) | 1.30(0.35-2.26) | 25.4(16.74-34.07) | 19.69(13.26-26.13) | 0.44(0.26-0.61) | 10.67(7.18-14.17) |
| 2038 | 48.43(30.34-66.51) | 1.30(0.27-2.34) | 25.57(16.07-35.08) | 19.74(12.71-26.76) | 0.43(0.25-0.62) | 10.69(6.88(-14.5) |
| 2039 | 48.82(29.04-68.59) | 1.30(0.18-2.43) | 25.76(15.38-36.15) | 19.79(12.15-27.43) | 0.43(0.23-0.63) | 10.71(6.57-14.85) |
| 2040 | 49.23(27.69-70.77) | 1.30(0.09-2.51) | 25.96(14.66-37.26) | 19.85(11.57-28.12) | 0.43(0.21-0.65) | 10.74(6.26-15.22) |
| 2041 | 49.67(26.29-73.06) | 1.30(0-2.60) | 26.17(13.92-38.43) | 19.92(10.98-28.85) | 0.43(0.20-0.66) | 10.76(5.93-15.6) |
| 2042 | 50.15(24.83-75.47) | 1.30(-0.09-2.69) | 26.41(3.14-39.66) | 20(10.38-29.62) | 0.43(0.18-0.67) | 10.8(5.6-16) |
| 2043 | 50.67(23.31-78.02) | 1.30(-0.19-2.78) | 26.65(12.34-40.96) | 20.09(9.76-30.43) | 0.42(0.16-0.68) | 10. 84(5.26-16.42) |
| 2044 | 51.21(21.73-80.69) | 1.29(-0.29-2.88) | 26.92(11.51-42.33) | 20.19(9.12-31.27) | 0.42(0.14-0.70) | 10.89(4.92-16.85) |
| 2045 | 51.78(20.08-83.47) | 1.29(-0.38-2.97) | 27.19(10.65-43.74) | 20.3(8.46-32.14) | 0.42(0.13-0.71) | 10.93(4.56-17.31) |
| 2046 | 52.37(18.36-86.38) | 1.29(-0.49-3.07) | 27.48(9.74-45.22) | 20.42(7.79-33.05) | 0.42(0.11-0.72) | 10.99(4.2-17.78) |
| 2047 | 53(16.57-89.43) | 1.29(-0.59-3.17) | 27.79(8.8-46.77) | 20.55(7.1-34.01) | 0.42(0.09-0.74) | 11.05(3.82-18.27) |
| 2048 | 53.67(14.69-92.64) | 1.29(-0.69-3.26) | 28.11(7.82-48.4) | 20.69(6.38-35) | 0.41(0.07-0.75) | 11.11(3.43-18.79) |
| 2049 | 54.35(12.73-95.98) | 1.28(-0.8-3.36) | 28.45(6.8-50.1) | 20.84(5.64-36.03) | 0.41(0.06-0.77) | 11.18(3.04-19.32) |
| 2050 | 55.06(10.68-99.45) | 1.28(-0.9-3.46) | 28.79(5.73-51.86) | 20.99(4.88-37.1) | 0.41(0.04-0.78) | 11.25(2.63-19.87) |

CI: confidence interval.

Table S12. ARIMA prediction model predicts the global age-standardized incidence and mortality of breast cancer from 2022 to 2050

| Forecast year | Age-standardized incidence rate (per 100,000) | | | Age-standardized mortality rate (per 100,000) | | |
| --- | --- | --- | --- | --- | --- | --- |
|  | Female | Male | Both | Female | Male | Both |
| 2022 | 46.67 | 0.94 | 24.70 | 14.53 | 0.33 | 7.88 |
| 2023 | 46.92 | 0.95 | 24.82 | 14.49 | 0.33 | 7.86 |
| 2024 | 47.15 | 0.96 | 24.94 | 14.45 | 0.32 | 7.83 |
| 2025 | 47.37 | 0.97 | 25.05 | 14.40 | 0.32 | 7.80 |
| 2026 | 47.59 | 0.98 | 25.16 | 14.34 | 0.32 | 7.76 |
| 2027 | 47.81 | 1.00 | 25.27 | 14.29 | 0.31 | 7.73 |
| 2028 | 48.03 | 1.01 | 25.37 | 14.23 | 0.31 | 7.69 |
| 2029 | 48.25 | 1.02 | 25.48 | 14.17 | 0.31 | 7.65 |
| 2030 | 48.47 | 1.03 | 25.59 | 14.11 | 0.30 | 7.62 |
| 2031 | 48.68 | 1.04 | 25.70 | 14.06 | 0.30 | 7.58 |
| 2032 | 48.90 | 1.06 | 25.80 | 14.00 | 0.30 | 7.54 |
| 2033 | 49.12 | 1.07 | 25.91 | 13.94 | 0.29 | 7.51 |
| 2034 | 49.34 | 1.08 | 26.02 | 13.88 | 0.29 | 7.47 |
| 2035 | 49.55 | 1.09 | 26.13 | 13.82 | 0.28 | 7.43 |
| 2036 | 49.77 | 1.10 | 26.23 | 13.76 | 0.28 | 7.39 |
| 2037 | 49.99 | 1.11 | 26.34 | 13.70 | 0.28 | 7.36 |
| 2038 | 50.21 | 1.13 | 26.45 | 13.64 | 0.27 | 7.32 |
| 2039 | 50.42 | 1.14 | 26.56 | 13.58 | 0.27 | 7.28 |
| 2040 | 50.64 | 1.15 | 26.66 | 13.52 | 0.27 | 7.25 |
| 2041 | 50.86 | 1.16 | 26.77 | 13.46 | 0.26 | 7.21 |
| 2042 | 51.08 | 1.17 | 26.88 | 13.40 | 0.26 | 7.17 |
| 2043 | 51.29 | 1.19 | 26.99 | 13.34 | 0.26 | 7.13 |
| 2044 | 51.51 | 1.20 | 27.09 | 13.28 | 0.25 | 7.10 |
| 2045 | 51.73 | 1.21 | 27.20 | 13.22 | 0.25 | 7.06 |
| 2046 | 51.94 | 1.22 | 27.31 | 13.16 | 0.25 | 7.02 |
| 2047 | 52.16 | 1.23 | 27.42 | 13.11 | 0.24 | 6.99 |
| 2048 | 52.38 | 1.25 | 27.52 | 13.05 | 0.24 | 6.95 |
| 2049 | 52.60 | 1.26 | 27.63 | 12.99 | 0.23 | 6.91 |
| 2050 | 52.81 | 1.27 | 27.74 | 12.93 | 0.23 | 6.87 |


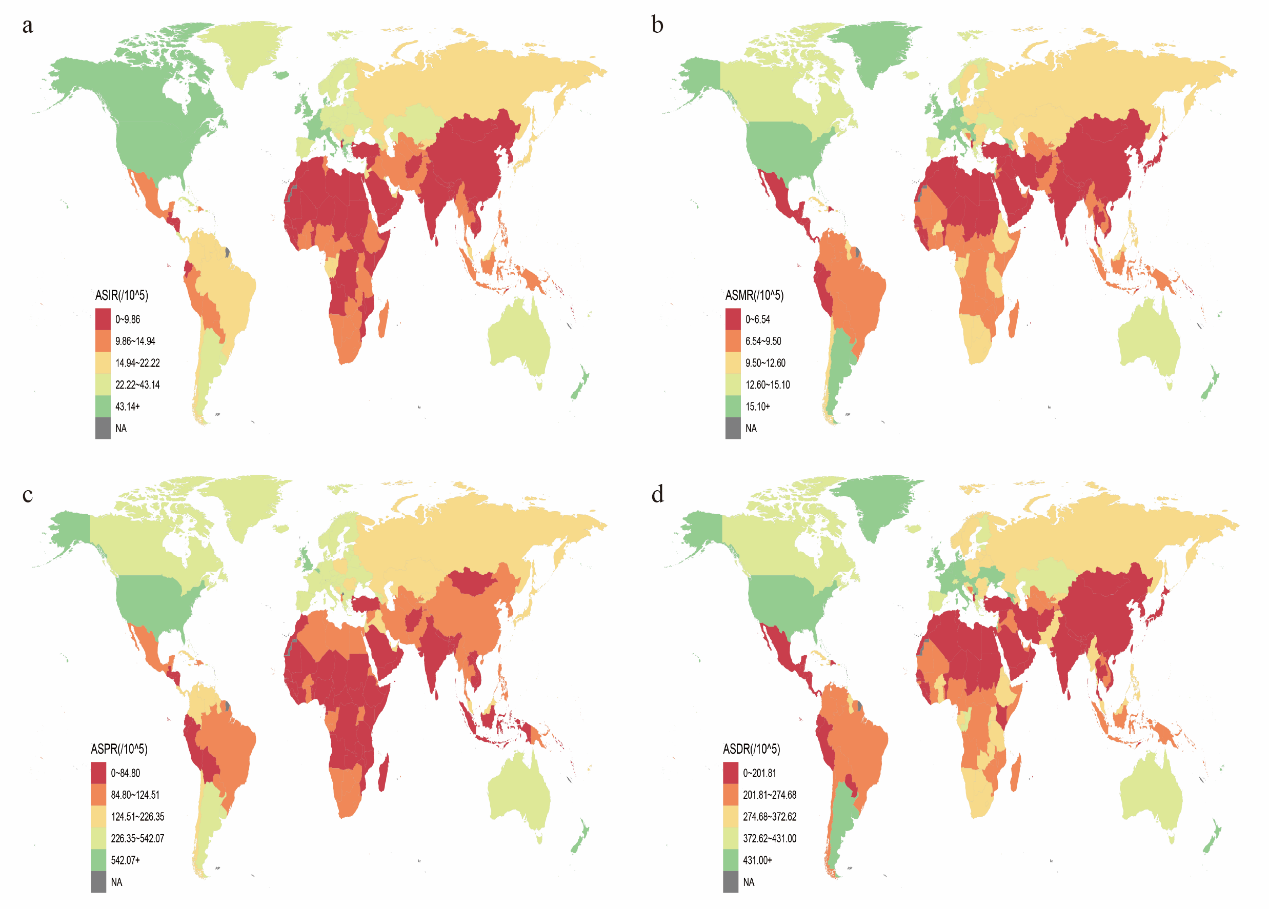


**Figure S1.** Global burden of breast cancer ASIR, ASMR, ASPR, and ASDR across 204 countries or territories in 1990. (a) ASIR; (b) ASMR; (c) ASPR; (d) ASDR. ASIR: age-standardized incidence rate; ASMR: age-standardized mortality rate; ASPR: age-standardized prevalence rate; ASDR: age-standardized DALYs rate; DALYs: disability-adjusted life years.


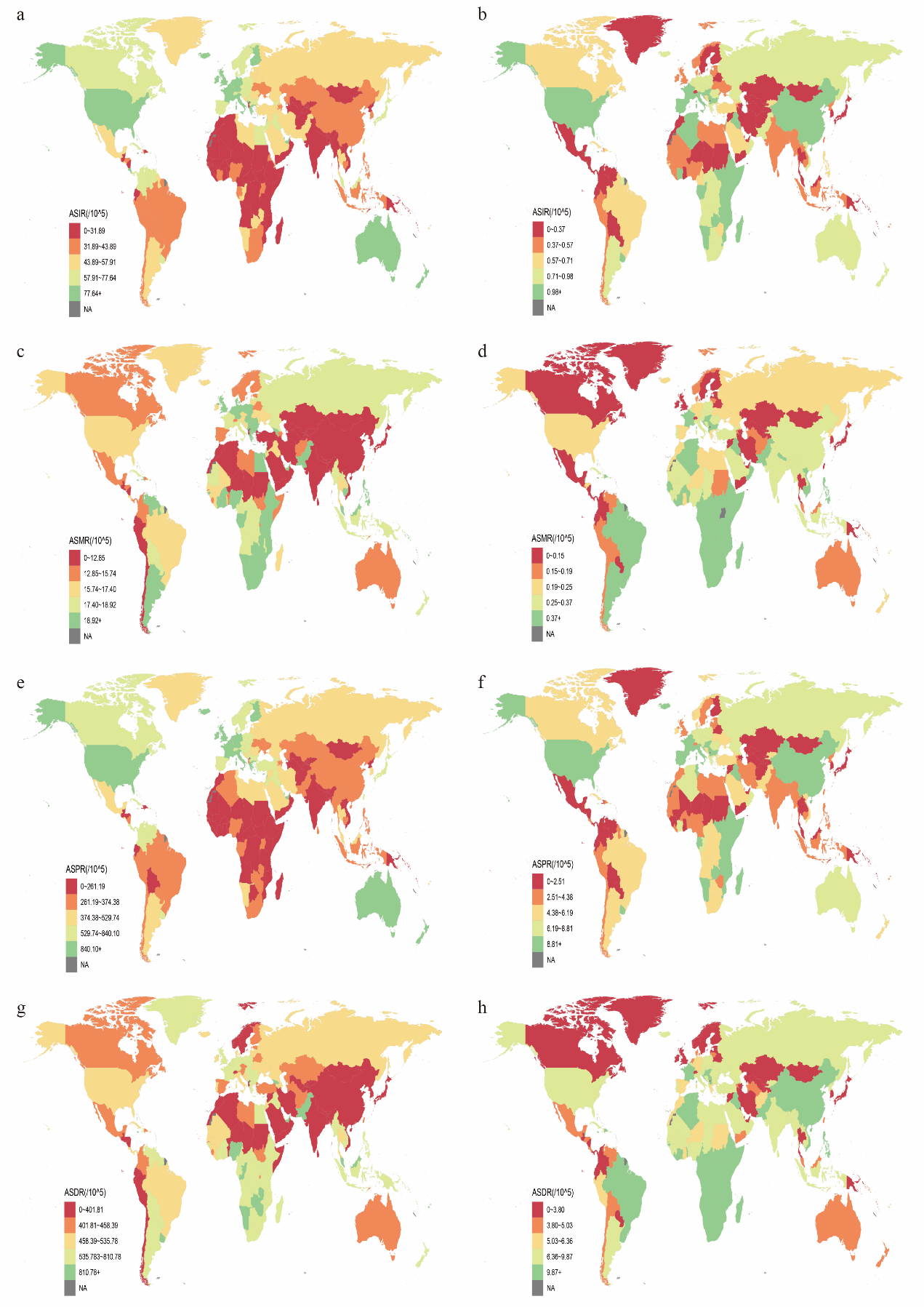


**Figure S2.** Global burden of breast cancer ASIR, ASMR, ASPR, and ASDR across 204 countries or territories in 2021 for female and male. (a) ASIR for female; (b) ASIR for male; (c) ASMR for female; (d) ASMR for male; (e) ASPR for female; (f) ASPR for male; (g) ASDR for female; (h) ASDR for male; ASIR: age-standardized incidence rate; ASMR: age-standardized mortality rate; ASPR: age-standardized prevalence rate; ASDR: age-standardized DALYs rate; DALYs: disability-adjusted life years.


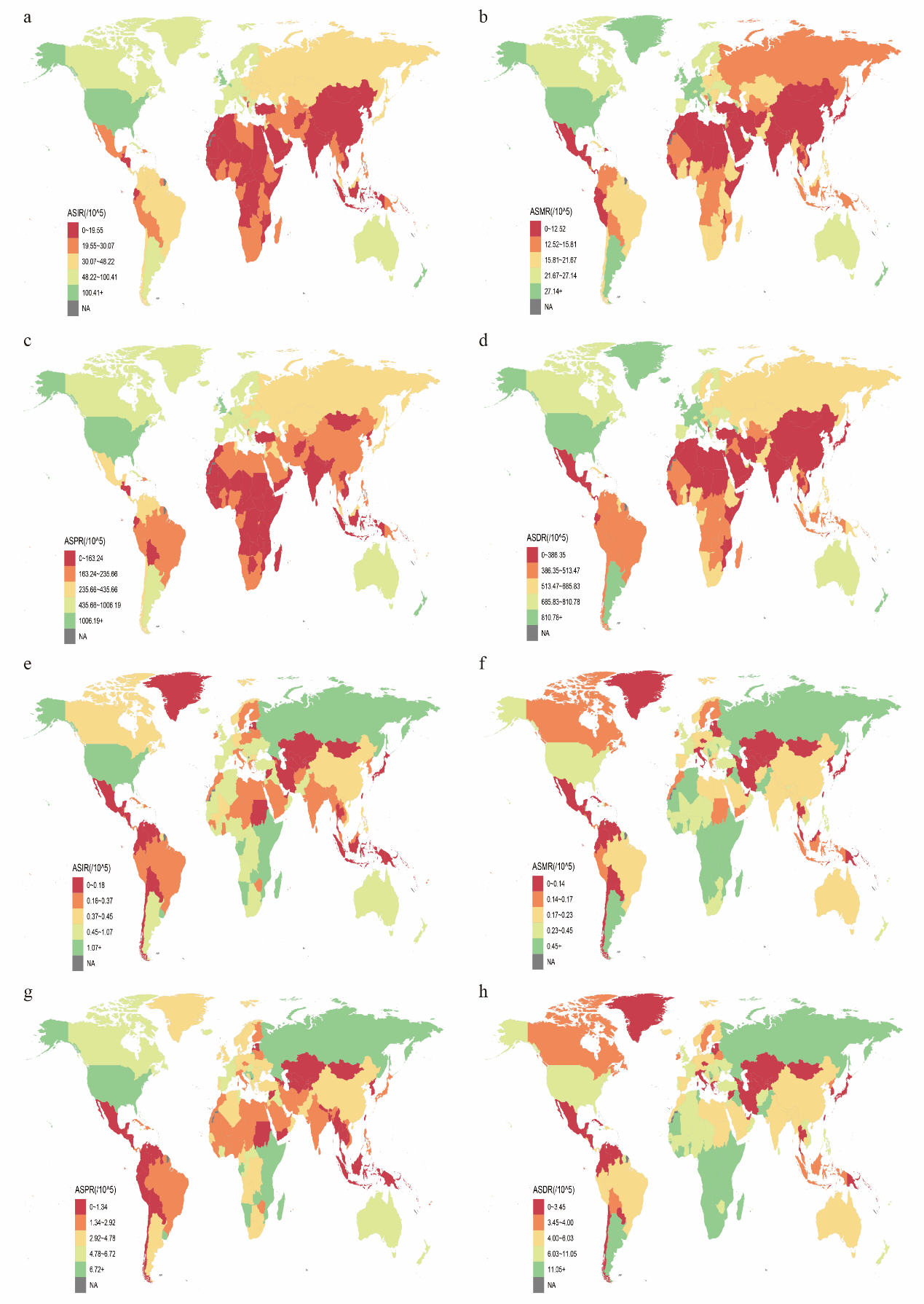


**Figure S3.** Global burden of breast cancer ASIR, ASMR, ASPR, and ASDR across 204 countries or territories in 2021 for female and male. (a) ASIR for female; (b) ASMR for female; (c) ASPR for female; (d) ASDR for female; (e) ASIR for male; (f) ASMR for male; (g) ASPR for male; (h) ASDR for male; ASIR: age-standardized incidence rate; ASMR: age-standardized mortality rate; ASPR: age-standardized prevalence rate; ASDR: age-standardized DALYs rate; DALYs: disability-adjusted life years.


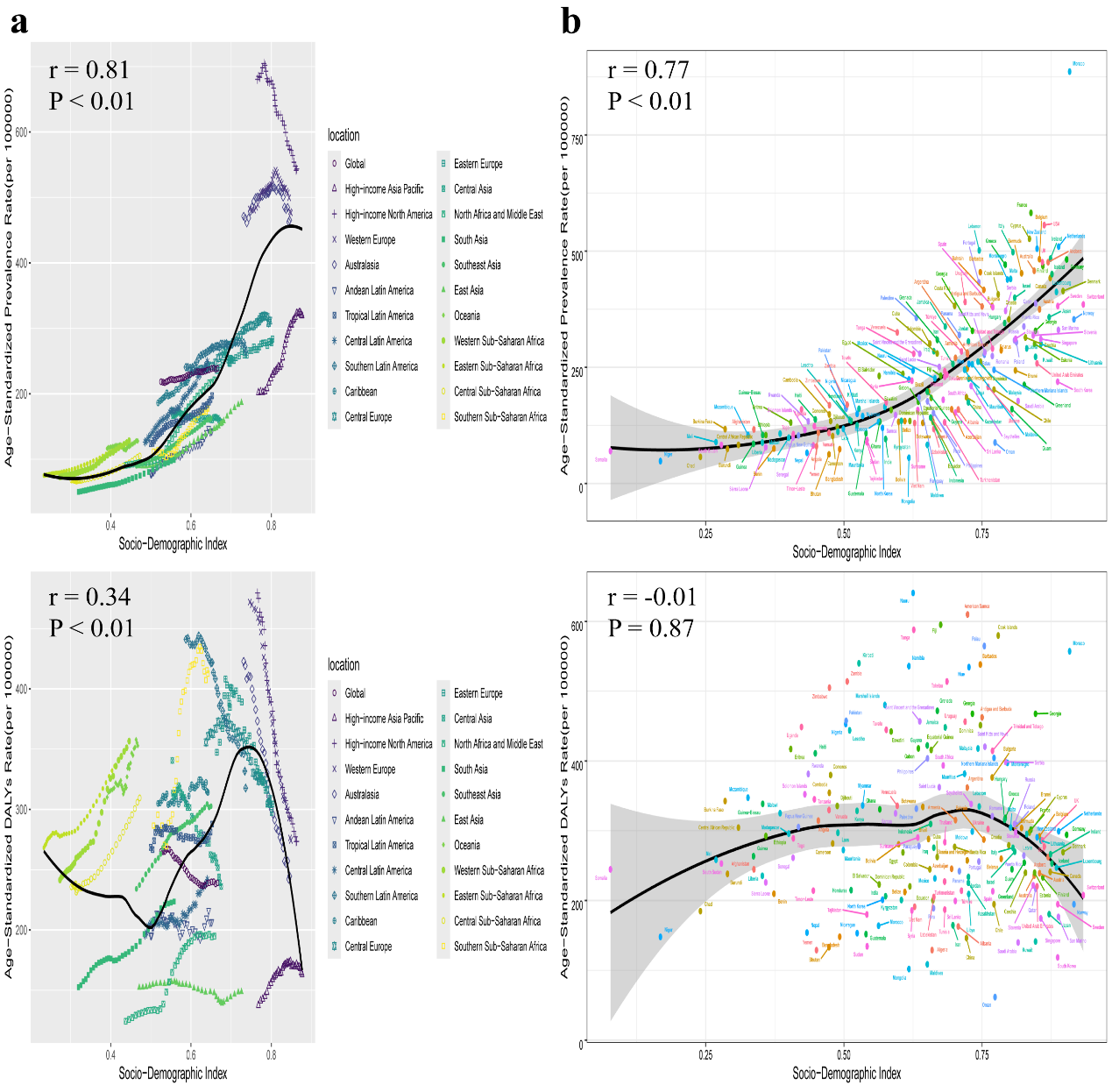


**Figure S4.** Age-standardized incidence and prevalence and DALYs for breast cancer across 21 GBD regions and 204 countries and territories by Socio-demographic Index from 1990 to 2021. (a) ASPR in 21 GBD regions; (b) ASPR in 204 countries; (c) ASDR in 21 GBD regions; (d) ASDR in 204 countries; Each colored line represents annual rates from 1990 to 2021 in a specified region, with expected values based on SDI and disease rates across all locations shown as the black line. SDI: Socio-demographic Index; ASPR: age-standardized prevalence rate; ASDR: age-standardized DALYs rate; DALYs: disability-adjusted life years; GBD: Global Burden of Disease.


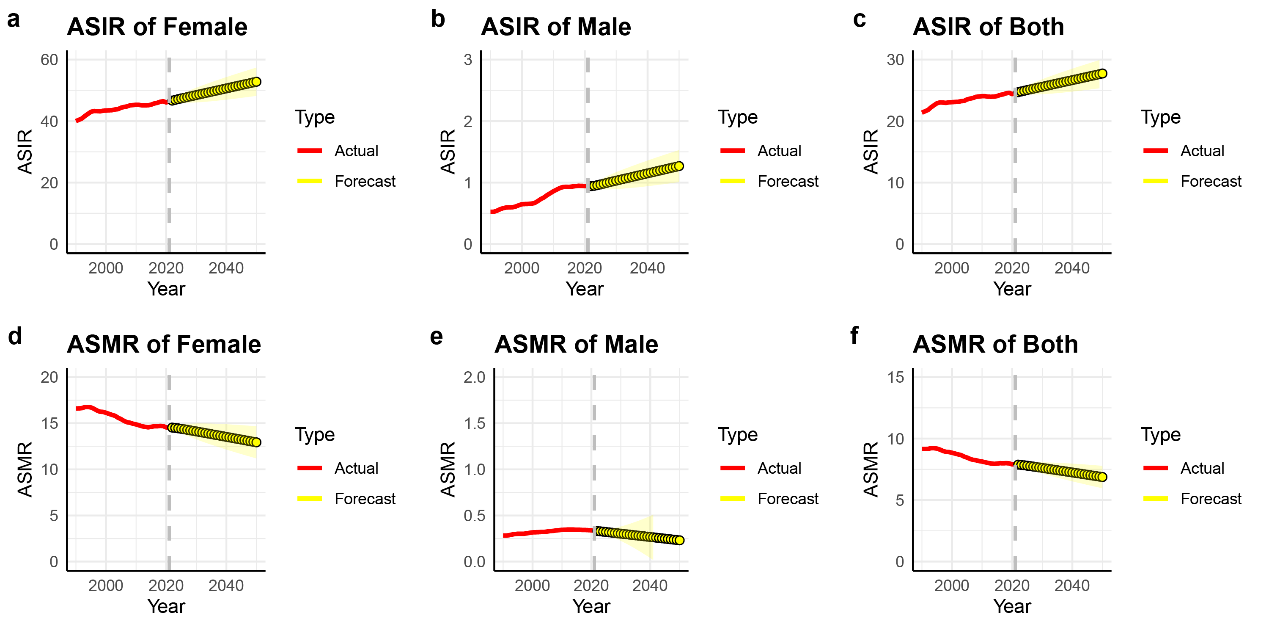


**Figure S5.** Trends of age-standardized incidence and mortality of breast cancer for female and male: actual rates (1990–2021) and forecast rates (2022–2050). (a) ASIR for female; (b) ASIR for male; (c) ASIR for both; (d) ASMR for female; (e) ASMR for male; (f) ASMR for both. The red line represents the actual rates. The yellow dots represent the forecast rates. The yellow region in shows the upper and lower limits of the 95% UI. ASIR: age-standardized incidence rate; ASMR: age-standardized mortality rate; UI: uncertainty interval.
